# A promiscuous mechanism to phase separate eukaryotic carbon fixation in the green lineage

**DOI:** 10.1038/s41477-024-01812-x

**Published:** 2024-10-09

**Authors:** James Barrett, Mihris I. S. Naduthodi, Yuwei Mao, Clément Dégut, Sabina Musiał, Aidan Salter, Mark C. Leake, Michael J. Plevin, Alistair J. McCormick, James N. Blaza, Luke C. M. Mackinder

**Affiliations:** 1 Department of Biology, University of York, York, UK; 2 Centre for Novel Agricultural Products (CNAP), Department of Biology, University of York, York, UK; 3 Institute of Molecular Plant Sciences, School of Biological Sciences, University of Edinburgh, Edinburgh, UK; 4 Centre for Engineering Biology, University of Edinburgh, Edinburgh, UK; 5 School of Physics, Engineering and Technology, University of York, York, UK; 6 York Structural Biology Laboratory, University of York, York, UK; 7 Department of Chemistry, University of York, York, UK

**Keywords:** Rubisco, Plant molecular biology, Cryoelectron microscopy

## Abstract

CO_2_ fixation is commonly limited by inefficiency of the CO_2_-fixing enzyme Rubisco. Eukaryotic algae concentrate and fix CO_2_ in phase-separated condensates called pyrenoids, which complete up to one-third of global CO_2_ fixation. Condensation of Rubisco in pyrenoids is dependent on interaction with disordered linker proteins that show little conservation between species. We developed a sequence-independent bioinformatic pipeline to identify linker proteins in green algae. We report the linker from *Chlorella* and demonstrate that it binds a conserved site on the Rubisco large subunit. We show that the *Chlorella* linker phase separates *Chlamydomonas* Rubisco and that despite their separation by ~800 million years of evolution, the *Chlorella* linker can support the formation of a functional pyrenoid in *Chlamydomonas*. This cross-species reactivity extends to plants, with the *Chlorella* linker able to drive condensation of some native plant Rubiscos in vitro and in planta. Our results represent an exciting frontier for pyrenoid engineering in plants, which is modelled to increase crop yields.

## Abstract (web summary)

Barrett et al. identify a key Rubisco phase-separating protein in the CO_2_-fixing pyrenoid of *Chlorella* algae. This protein’s broad promiscuity for green lineage Rubiscos may aid in engineering CO_2_-supercharging pyrenoids in plants to boost yields.

## Main

**Fig. 1:**
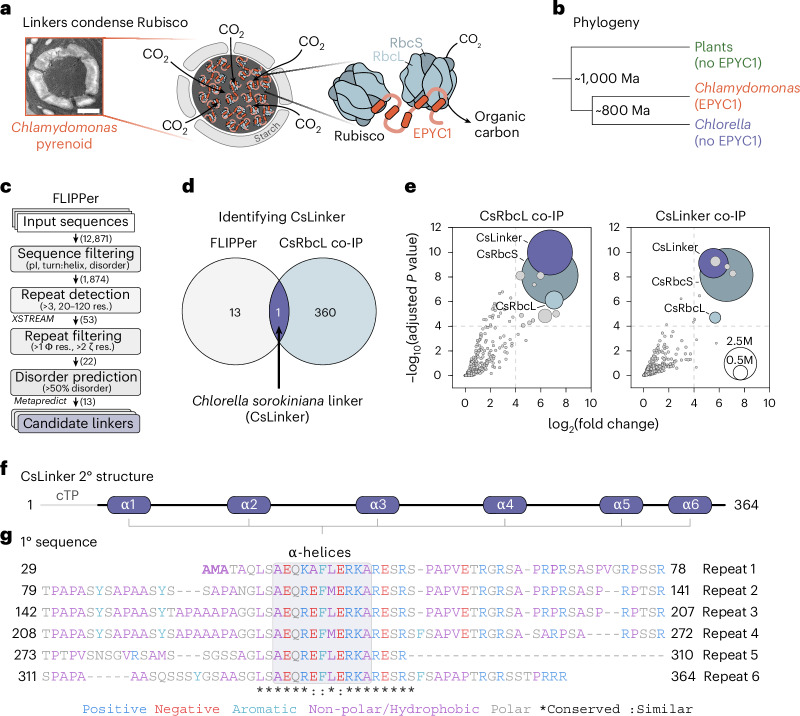
Identification of the *Chlorella sorokiniana* linker protein (CsLinker). **a**, TEM of the *Chlamydomonas reinhardtii* pyrenoid (*n* = 1, single observation), with adjacent schematic of Rubisco condensation in the pyrenoid by interaction of EPYC1 helices with the Rubisco small subunits (RbcSs). Condensed Rubisco fixes CO_2_ to organic carbon. Scale bar, 1 μm. **b**, Phylogeny of *Chlamydomonas*, *Chlorella* and plants. Estimated divergence points from a time-calibrated phylogeny^28^. **c**, Schematic representation of FLIPPer used to identify candidate linkers that share features with EPYC1. Where relevant, the programme used is indicated. The number of sequences remaining after each filtering step of the *Chlorella sorokiniana* UTEX1230 genome is indicated. pI, isoelectric point; res., residue; Φ, hydrophobic; ζ, electrostatic. **d**, Venn diagram demonstrating identification of CsLinker from FLIPPer and CsRbcL co-immunoprecipitation followed by mass spectrometry (co-IP). **e**, Reciprocal co-IP experiments performed using antibodies raised to the Rubisco large subunit (left) and CsLinker (right). Dashed lines indicate arbitrary significance thresholds (−log_10_[adjusted *P* value] > 4, log_2_[fold change] > 4), above which points are sized according to their summed intensity (M, millions) following the inset key, from 3 biological replicates. **f**, Predicted secondary structure of CsLinker from AlphaFold modelling (Supplementary Fig. 1). The predicted chloroplast transit peptide (cTP) and α-helices (α1–6) are indicated. **g**, Primary sequence alignment of the six repeat regions of CsLinker, coloured by residue property.

As the primary gateway between atmospheric carbon dioxide (CO_2_) and organic carbon, ribulose-1,5-bisphosphate carboxylase/oxygenase (Rubisco) fixes ~400 gigatons of CO_2_ annually^1^. Despite this huge global productivity, Rubisco as an enzyme is catalytically slow^2^. Its Archaean origin^3^ and exceptionally slow evolutionary trajectory^4^ has meant that Rubisco has failed to substantially overcome the supposed trade-off between specificity for its substrate (CO_2_ or O_2_) and catalytic rate^4,5^. These shortfalls mean that Rubisco is often limiting for photosynthesis in most plants (that is, C_3_ plants)^6–11^. Accordingly, C_3_ plants compensate by producing and maintaining large amounts of Rubisco^12,13^. By mass, Rubiscos in aquatic phototrophs (algae and cyanobacteria) are ~20 times more efficient^14^. These Rubiscos benefit from operating in biophysical CO_2_-concentrating mechanisms (CCMs) that increase the CO_2_:O_2_ ratio at their active site. A large proportion of aquatic CO_2_ fixation occurs in the pyrenoid^15^, a subcompartment of the chloroplast found in most eukaryotic algae and some basal land plants that is the centrepiece of their biophysical CCMs^16^. Pyrenoid formation is underpinned by biomolecular condensation of Rubisco by disordered linker proteins^15,17^ (Fig. 1a). Engineering pyrenoid-based CCMs in crop plants that operate C_3_ photosynthesis is a promising avenue to increase their primary productivity and reduce nitrogen and water usage^18,19^. While significant progress has been made in the characterization and transfer of pyrenoid components from the model alga *Chlamydomonas reinhardtii* to the model C_3_ plant *Arabidopsis thaliana*^15,20–24^, our knowledge of pyrenoids from other species remains limited. By characterizing pyrenoids from other species, we hope to expand the toolbox available for plant pyrenoid engineering and gain insight into the commonalties and differences in pyrenoid assembly.

Here we identify and characterize CsLinker, a pyrenoid linker protein from the green alga *Chlorella* which is an ancient relative of *Chlamydomonas*. Using biochemical and structural approaches, we demonstrate that CsLinker is functionally analogous to linker proteins from other organisms, despite low sequence identity. Crucially, and in contrast to the *Chlamydomonas* linker EPYC1, we show that CsLinker binds to the Rubisco large subunit (RbcL). The high conservation of the binding interface on the RbcL supports functional cross-reactivity of *Chlamydomonas* Rubisco and CsLinker as well as CsLinker-mediated condensation of native plant Rubiscos both in vitro and in planta. These findings represent a significant advance towards the engineering of synthetic pyrenoids and overcome a major hurdle for the future engineering of pyrenoid-based CCMs in plants.

## Results

### Fast Linker Identification Pipeline for Pyrenoids (FLIPPer) identifies the pyrenoid linker in *Chlorella*

**Extended Data Fig. 1:**
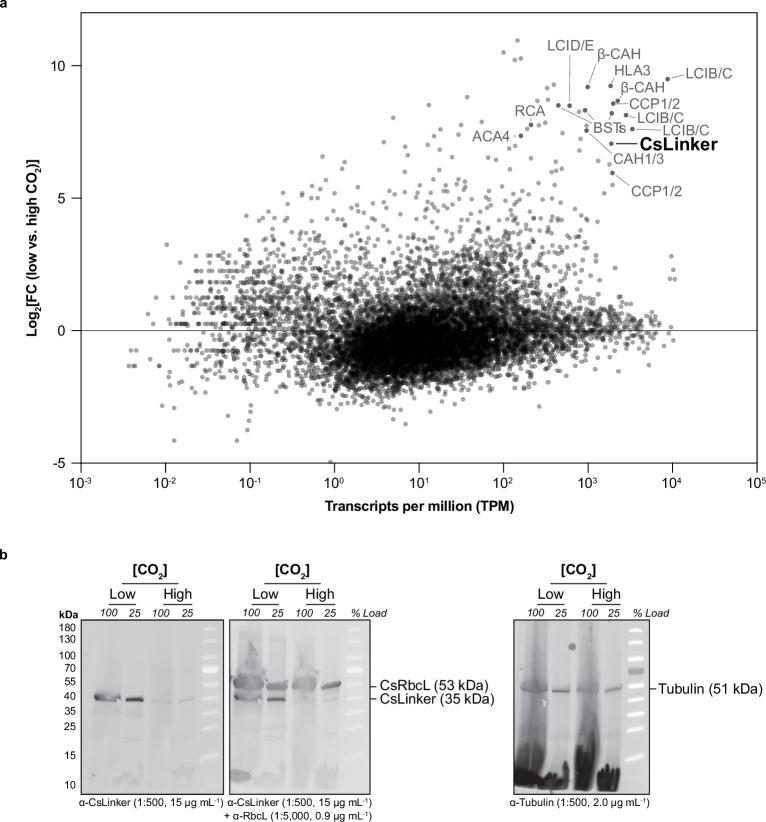
Low CO_2_ inducibility of CsLinker. **a**, Differential gene expression analysis completed using publicly available RNA-seq data (PRJNA343632) comparing transcript abundance at high CO_2_ (5%) and 30 minutes after switching to 0.01% CO_2_ culture conditions. Homologues of low CO_2_ induced CCM genes in *Chlamydomonas* are indicated alongside CsLinker. **b**, Western blot analysis of CsLinker and CsRbcL protein abundance under low (0.04%) and high (3%) CO_2_ conditions. Tubulin was used as a loading control on the same blot, and detected with a separate secondary antibody. This observation was from a single, non-repeated experiment.

The *Chlamydomonas* pyrenoid linker protein EPYC1 is not conserved outside closely related species^15^ (Fig. 1b). To identify functional analogues in other pyrenoid-containing species of the green lineage, we developed a sequence homology-independent Fast Linker Identification Pipeline for Pyrenoids (FLIPPer). FLIPPer searches for proteins with features key to the function of EPYC1, namely: (1) largely disordered, with (2) repeating structured elements that are (3) spaced at a feasible length scale for cross-linking Rubiscos (20–120 residues; ~2–8 nm^25,26^) (Fig. 1c). Among our analyses, we identified 13 proteins with these features in the genome of the unicellular trebouxiophyte *Chlorella sorokiniana* UTEX1230 (ref. ^27^) (Chlorella hereafter) (Supplementary Fig. 1), which diverged from *Chlamydomonas* ~800 million years ago (Ma)^28^ (Fig. 1b). To validate the identity of the Chlorella linker, we completed co-immunoprecipitation followed by mass spectrometry (co-IP) experiments on Chlorella cells grown in low CO_2_, using an antibody specific to the RbcL (Fig. 1e, Supplementary Fig. 2a,b and Table 1). Of the 360 proteins enriched relative to control co-IPs (*Chlamydomonas* BST2 antibody), only 1 was shared among the FLIPPer outputs (Fig. 1d). This protein, CSI2_123000012064, was the most significantly enriched in the RbcL co-IP experiment, alongside both subunits of the Chlorella Rubisco holoenzyme (CsRbcL and CsRbcS) (Fig. 1e, left). We called this protein CsLinker. We subsequently completed reciprocal co-IPs using a CsLinker antibody (Supplementary Fig. 2c,d), which enriched both CsRbcL and CsRbcS, indicating ex vivo complex formation between CsLinker and Chlorella Rubisco (Fig. 1e, right). We confirmed the CsLinker gene sequence, gene model and predicted chloroplast transit peptide (cTP) by PCR and sequencing, and through mapping of peptides identified in mass spectrometry experiments (Supplementary Fig. 3). We further confirmed the low CO_2_ inducibility of CsLinker through analysis of available RNA-seq data and by western blotting (Extended Data Fig. 1 and Supplementary Table 2). While AlphaFold 2 modelling demonstrated that CsLinker and EPYC1 share clear structural analogy, their primary sequences share little similarity (Fig. 1g, 25% identity; Supplementary Fig. 4), reflecting their independent origins. Like EPYC1, BLAST analysis of CsLinker in the NCBI non-redundant database also indicated that homologues of CsLinker are only conserved in closely related species (Supplementary Table 3).

### CsLinker is a bona fide pyrenoid linker protein

**Fig. 2:**
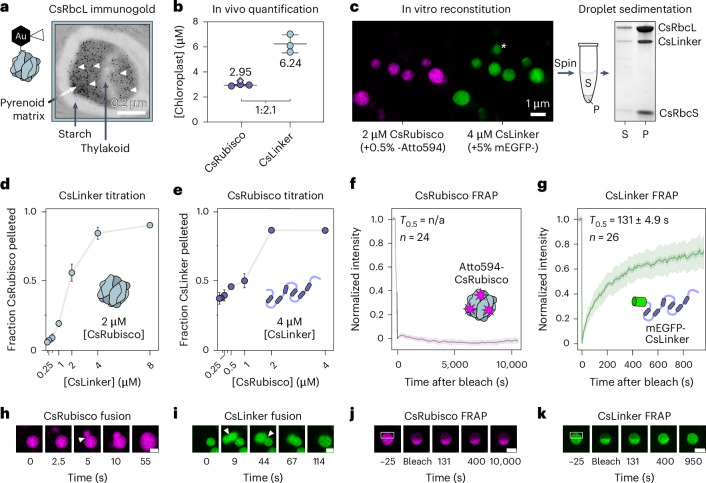
CsLinker phase separates Rubisco at physiological conditions. **a**, Representative immunogold TEM of the Chlorella pyrenoid after primary incubation with the RbcL antibody. A subset of gold nanoparticles are indicated by white arrowheads. **b**, Absolute quantification of CsRubisco holoenzyme (derived from CsRbcL) and CsLinker in vivo (*n* = 3). The ratio between CsRubisco and CsLinker is indicated at the bottom (see Supplementary Fig. 6 for details). Mean ± s.d. of 3 biological replicates. The asterisked point indicates the independent quantification from western blotting (Supplementary Fig. 2b). **c**, Left: confocal fluorescence microscopy image of droplets in the in vitro reconstitution of the Chlorella pyrenoid formed at concentrations of CsRubisco and CsLinker close to that of the chloroplast (2 μM and 4 μM, respectively). Asterisk indicates a droplet that settled between imaging of the two channels. Atto594-CsRubisco and mEGFP-CsLinker were incorporated at 0.5% and 5% molar concentrations, respectively. Single, non-repeated observation. Right: droplets can be sedimented by centrifugation and the composition of the pellet (P) relative to the supernatant (S) analysed by SDS–PAGE (repeated observation; see Supplementary Figs. 8 and 9). This experiment format was used to generate datapoints in **d** and **e**. **d**, Titration droplet sedimentation assays with fixed CsRubisco concentration. **e**, Titration droplet sedimentation assays with fixed CsLinker. Where visible in **d** and **e**, data are mean ± s.d. of 2 technical replicate experiments completed concurrently (Supplementary Fig. 7b,c). **f**, Average full-scale normalized half-FRAP recovery curve of Atto594-CsRubisco in droplets formed as in **c**. **g**, Average full-scale normalized half-FRAP recovery curve of mEGFP-CsLinker in droplets with the same composition. In **f** and **g**, the mean, s.e.m. and s.d. of the indicated number of technical replicates are represented by the line, the smaller shaded region and the larger shaded region, respectively. **h**, Time series of 0.5% (molar ratio) Atto594-CsRubisco-labelled droplets formed as in **c**, undergoing fusion (arrowhead) and relaxation. **i**, 5% mEGFP-CsLinker-labelled droplets undergoing consecutive fusions. **j**, Time series of a representative CsRubisco FRAP experiment, as quantified in **f**. The white box indicates the region bleached. **k**, Representative CsLinker FRAP experiment, as quantified in **g**. Scale bars for **h**–**k**, 1 μm. All in vitro droplet experiments in **c**–**k** were completed in a 50 mM Tris-HCl pH 8.0, 50 mM NaCl buffer. Source data

**Extended Data Fig. 2:**
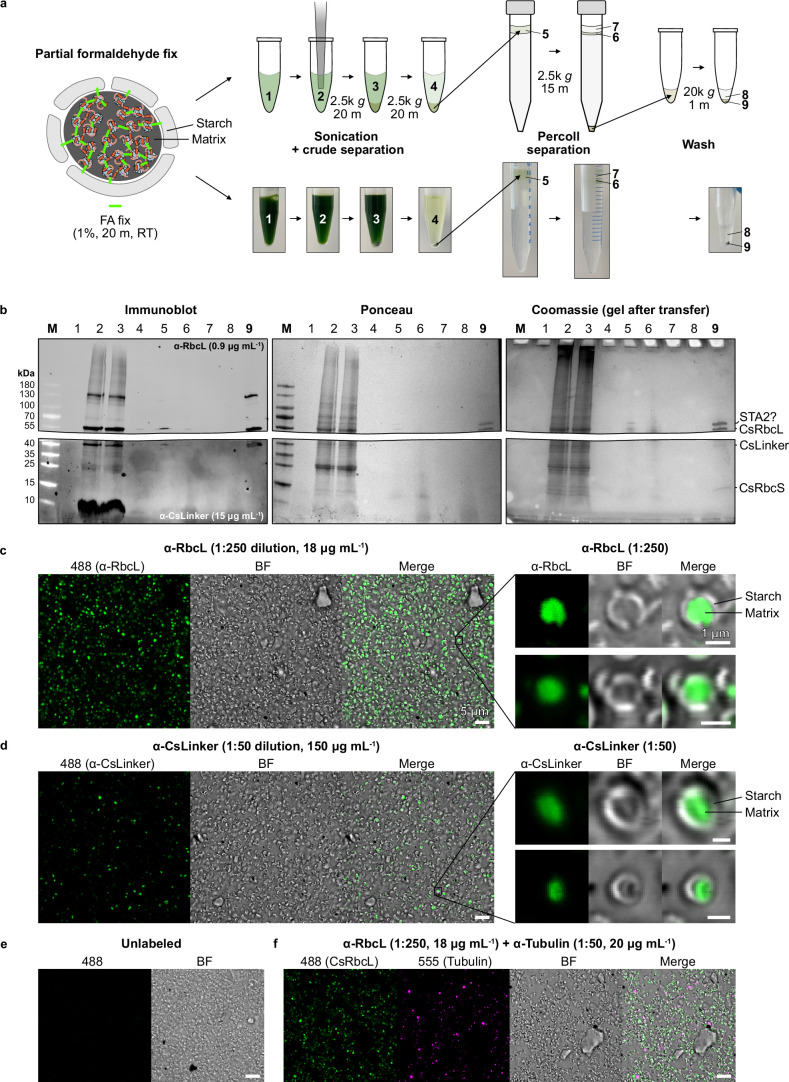
Localization of CsRubisco and CsLinker in the pyrenoid. **a**, Protocol used for the enrichment of pyrenoids from *Chlorella* cells. Fractions are labelled according to their analysis in b. **b**, Western blot analysis of CsRbcL and CsLinker in fractions throughout pyrenoid enrichment. The membrane was cut following Ponceau staining and incubated separately with the indicated primary antibodies. The granule-bound starch synthase (CSI2_123000003711, 59.7 kDa homolog of STA2 from Chlamydomonas) is putatively annotated as a major component of pyrenoid starch, visible in the Coomassie and Ponceau panels. **c**, Immunofluorescence localization of CsRbcL in pyrenoid-enriched fractions. Confocal microscopy images of immunolabelled fraction ‘9’ from panels d/e. The immunofluorescent signal from CsRbcL is present within the surrounding starch sheath, indicating a pyrenoid matrix localization. **d**, Immunofluorescence localization of CsLinker in pyrenoid-enriched fractions. Although the degree of labelling is lower due to the poorer antigenicity of the primary antibody, the localization pattern is again consistent with the pyrenoid matrix. **e**, Confocal microscopy image of the unlabeled pyrenoid-enriched fraction, showing a lack of signal. **f**, Confocal microscopy image of pyrenoid-enriched fractions immunolabelled with both RbcL and Tubulin primary antibodies, detected with separate secondary antibodies. The low level of non-specific fluorescence from the Tubulin antibody does not co-localize with the RbcL signal, indicating specific labelling by the RbcL and CsLinker antibodies in panels g and h. All results were obtained from a single non-repeated pyrenoid-enrichment protocol of a single biological replicate.

Having demonstrated ex vivo interaction between CsLinker and CsRubisco (Fig. 1e), we sought to confirm that both were abundant components of the Chlorella pyrenoid in vivo. Using the RbcL antibody, we completed immunoelectron microscopy and observed CsRubisco almost exclusively localized in the pyrenoid (96.2 ± 3.5% s.d., *n* = 6) (Fig. 2a, Supplementary Fig. 5 and Table 4). While we observed no immunogold labelling using the CsLinker antibody (Supplementary Fig. 6c), we were able to localize both CsLinker and CsRubisco in the matrix of pyrenoid-enriched fractions by immunofluorescence (Extended Data Fig. 2). To determine the abundance of CsLinker and CsRubisco in vivo, we completed absolute quantification mass spectrometry using standard curves of recombinant CsLinker and CsRubisco purified from Chlorella. Accounting for the chloroplast volume^29^, we approximate the concentration of CsLinker and CsRubisco holoenzyme to be 6.24 ± 0.73 μM s.d. and 2.95 ± 0.06 μM s.d., respectively (Fig. 2b, Supplementary Fig. 6 and Tables 5–8), demonstrating that CsLinker is highly abundant in the chloroplast. We validated the Rubisco quantification by western blotting (3.30 μM; asterisk in Fig. 2b, Supplementary Fig. 2b and Table 9). The localization of both CsLinker and CsRubisco in the pyrenoid (Fig. 2a and Extended Data Fig. 2), the twofold abundance of CsLinker over CsRubisco in vivo (Fig. 2b) and their ex vivo interaction (Fig. 1e) indicate that CsLinker probably interacts with CsRubisco as an abundant component of the Chlorella pyrenoid in vivo.

**Extended Data Fig. 3:**
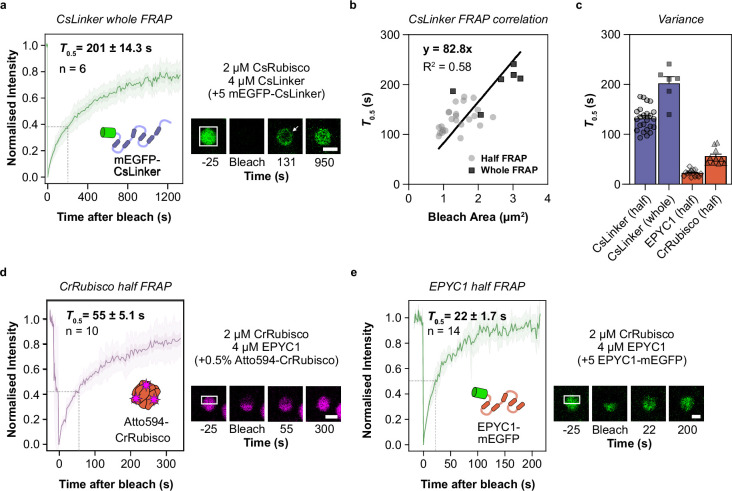
FRAP analysis of Chlorella and *Chlamydomonas in vitro* reconstitutions. **a**, Average FRAP recovery curve from whole FRAP experiments completed according to reference images adjacent where the bleach region is indicated by the box and the scale bar = 1 μm. The arrow highlights recovery of the signal from the periphery of the droplet, indicating external exchange. The standard error of the *T*_0.5_ is indicated in the plot and the dashed lines indicate the *T*_0.5_ on the plot. **b**, Correlation of *T*_0.5_ with the area of the bleached region in whole and half FRAP experiments of CsLinker, explaining the longer *T*_0.5_ in whole FRAP experiments. **c**, Variance of *T*_0.5_ values derived from individual fits of recovery curves. Errors bars represent standard error of the mean for n = 24, 6, 14 and 10 technical replicate measurements in each sample respectively. **d**, Average half FRAP recovery curve of Atto594-CrRubisco in the *Chlamydomonas* reconstitution. **e**, Average half FRAP recovery curve of EPYC1-mEGFP in the *Chlamydomonas* reconstitution In panels **a**, **b**, **d** and **e** the mean, S.E.M and S.D. of the indicated number of technical replicates are represented by the line, the smaller shader region, and the larger shaded region respectively.

The *Chlamydomonas* pyrenoid is a liquid–liquid phase-separated (LLPS) biomolecular condensate in vivo^26^, which is essential for Rubisco packaging and pyrenoid function^15^. In vitro, reconstituted pyrenoids formed by mixing purified linker EPYC1 and *Chlamydomonas* Rubisco demonstrate similar properties to in vivo^30^. Accordingly, we sought to understand whether mixing CsLinker and CsRubisco gives rise to similar emergent properties in vitro. When mixed at concentrations close to those we approximated in the chloroplast (Fig. 2b), we observed demixing into micron-scale droplets that was dependent on and incorporated both CsLinker and CsRubisco (Fig. 2c and Supplementary Fig. 7a,b). To assess the relative occupancy of the components within droplets, we completed reciprocal titration in droplet sedimentation assays (Fig. 2c, right). By separately fixing both components, we observed a requirement for a ~2-fold excess of CsLinker to fully demix both components (Fig. 2d,e and Supplementary Fig. 7c,d). This observation agreed with the ~2-fold greater abundance of CsLinker than CsRubisco we measured in vivo (Fig. 2b) and previous observations in *Chlamydomonas*^30^. Using the same ratio, we observed a critical global concentration (0.3–0.5 μM) and salt dependency for droplet formation (Supplementary Fig. 8); both key indicators of LLPS^31^. To further understand the droplet properties, we completed fluorescence recovery after photobleaching (FRAP) experiments, which allowed us to monitor the mobility of labelled CsLinker and CsRubisco. Strikingly, we observed largely different mobilities for CsRubisco and CsLinker in droplets. While mEGFP-CsLinker exhibited exchange with the dilute phase in whole FRAP experiments, with a half-maximal recovery time (*T*_0.5_) of 201 ± 14.3 s s.e.m. (Extended Data Fig. 3a and Supplementary Table 10), and internal mixing in half-FRAP experiments (*T*_0.5_ = 131 ± 4.89 s s.e.m.; Fig. 2g,k), Atto594-CsRubisco demonstrated no mixing or exchange over hour timescales (Fig. 2f,j). However, both CsLinker and CsRubisco appeared to undergo internal rearrangement over second timescales upon droplet fusion (Fig. 2h,i), suggesting that CsRubisco is not immobilized in droplets, akin to observations of the *Phaeodactylum tricornutum* pyrenoid reconstitution^17^. To ensure that our observations were not set-up specific, we confirmed the mobility of *Chlamydomonas* Rubisco (CrRubisco) and EPYC1 in the *Chlamydomonas* pyrenoid reconstitution using the same strategy. Consistent with previous in vitro^30^ and in vivo^26^ observations, both components were highly mobile (EPYC1-mEGFP half-FRAP *T*_0.5_ = 22 ± 6.3 s s.e.m., Atto594-CrRubisco half-FRAP *T*_0.5_ = 55 ± 5.1 s s.e.m.; Extended Data Fig. 3d,e). Taken together, these results demonstrate the ability of CsLinker to phase separate Chlorella Rubisco in vitro, and alongside our ex vivo and in vivo observations, indicate that this process probably underpins pyrenoid formation in vivo, analogous to EPYC1 in *Chlamydomonas*.

### CsLinker binds to the RbcL

**Fig. 3:**
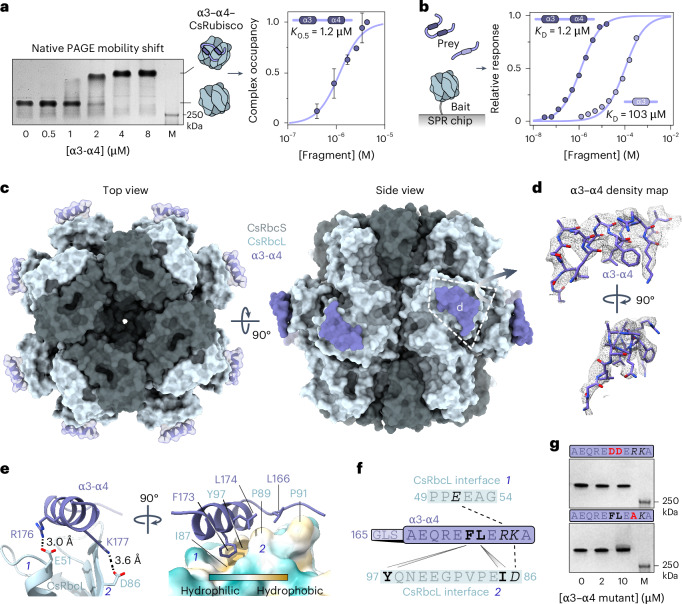
CsLinker binds to the RbcL. **a**, α3–α4 forms a stable complex with CsRubisco. Representative native PAGE gel-shift assay (left) demonstrating formation of higher-order α3–α4–CsRubisco complex in a 50 mM Tris-HCl pH 8.0, 50 mM NaCl buffer. Mean ± s.d. of 2 technical replicate experiments completed independently (right) taken from Supplementary Fig. 10h. **b**, Schematic of SPR experiments (left) used to determine binding affinity of α3 and α3–α4 for CsRubisco (right). SPR response normalized to *B*_max_ value obtained from fit of raw data; *n* = 3; error bars, s.d. **c**, Top (left) and side (right) views of surface representations of cryo-EM determined structure of the α3–α4–CsRubisco complex. The modelled α-helix of α3 is superimposed on each CsRbcL on the basis of the coordinates built into one axis of the C1 map, as shown in **d**. **d**, Density map of the α3–α4 region in the C1 complex map, carved with a radius of 2 from the built coordinates, at a contour level of 0.033. **e**, Molecular interactions at the interface. Shortest range electrostatic interactions highlighted by PDBePISA (left) and residues contributing to the hydrophobic interface (right). The surface of CsRbcL is coloured according to hydrophobicity. Residues are numbered according to their position in the full-length CsLinker. **f**, Map of the interactions between the α-helices of α3–α4 and the two interfaces on CsRbcL. Dashes indicate salt bridges, wedges represent significant contributions to the hydrophobic interface, with italicized and bolded residues contributing the same interfaces, respectively. **g**, Native PAGE gel-shift assays showing that mutation of α3–α4 disrupts binding to CsRubisco. The sequence of the α-helices in each fragment is provided above each image. From a single, non-repeated observation. Source data

Previously characterized Rubisco-condensing linker proteins from algal pyrenoids^17,32^ and bacterial carboxysomes^33,34^ utilize structured regions to bind different regions on Rubisco. We hypothesized that the predicted α-helical regions in CsLinker may bind to Rubisco and that this interaction would involve a previously uncharacterized interface. To characterize the interaction, we produced fragments of CsLinker encompassing entire repeat sequences centred on predicted α-helix 3 (α3) and α-helices 3 and 4 (α3–α4) (Fig. 1f,g and Supplementary Fig. 9a). Using two-dimensional (2D) nuclear magnetic resonance (NMR) spectroscopy, we confirmed the only stable structure in the fragments to be α-helices of ~10 residues, in line with structural predictions (Fig. 1f and Supplementary Figs. 4a and 9). The similarity of the NMR spectra of α3 and α3–α4 indicated that the individual repeat regions have similar overall properties (Supplementary Fig. 9d), consistent with their similar residue composition and the lack of stable tertiary structure. We next tested the ability of the fragments to interact with CsRubisco. In line with the dependency of LLPS on multivalent interactions for cross-linking, the single helix α3 fragment was unable to phase separate CsRubisco, but did demonstrate concentration-dependent mobility shift of CsRubisco in native polyacrylamide gel electrophoresis (PAGE) experiments (Supplementary Fig. 10a–c). Surprisingly, the α3–α4 double repeat fragment was also unable to induce LLPS but did demonstrate formation of a stable higher-order complex with CsRubisco that had a half-occupancy (*K*_0.5_) of ~1.2 μM (95% confidence interval (CI_95_): 0.93–1.42 μM) (Fig. 3a, Supplementary Fig. 10d–h and Table 11). To measure the affinity of the fragments for CsRubisco, we used surface plasmon resonance (SPR) experiments in which CsRubisco was immobilized as bait, and the α3 and α3–α4 fragments were used as prey (Fig. 3b, left). We measured a similar affinity (*K*_D_) of the α3–α4 fragment for CsRubisco (1.21 μM, CI_95_: 1.10–1.33 μM) as we observed for the *K*_0.5_ of the α3–α4–CsRubisco complex by native PAGE (Fig. 3a, Supplementary Fig. 11 and Table 12). The *K*_D_ of the α3 fragment was ~100-fold higher (103 μM, CI_95_: 80–133 μM) than that of α3–α4, consistent with cooperative binding of the two α-helical regions in α3–α4 to the same Rubisco rather than other Rubiscos in solution. This explained the lack of LLPS and indicated that the higher-order complex observed by native PAGE probably consists of single Rubiscos bound by α3–α4.

**Extended Data Fig. 4:**
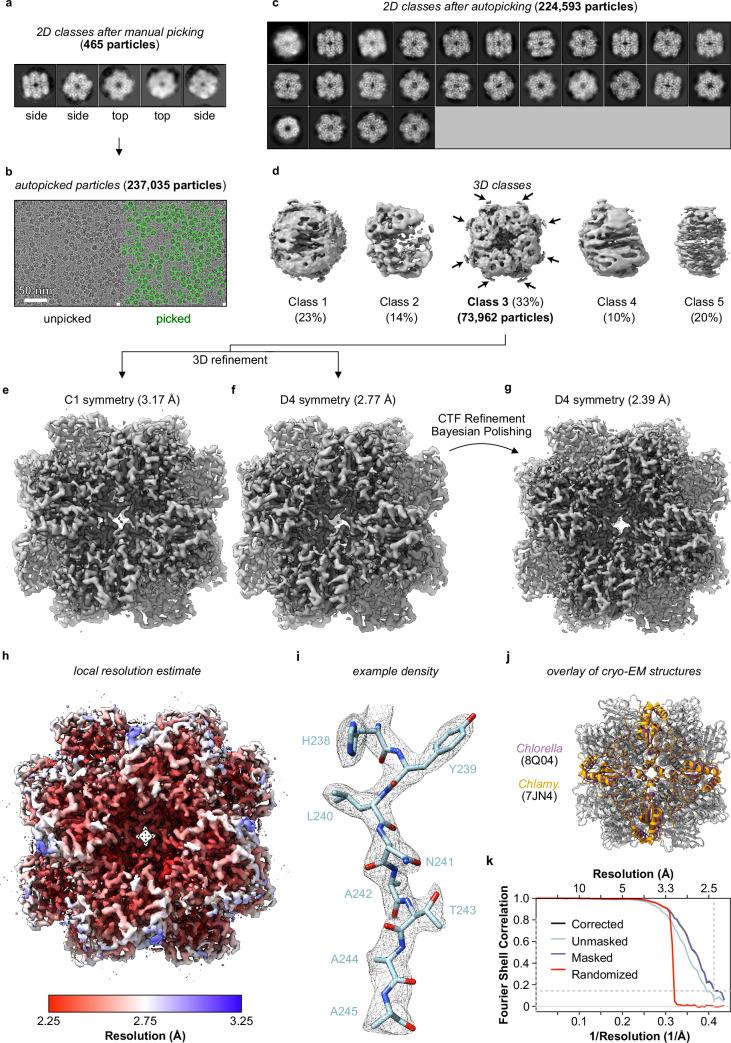
Cryo-EM data processing of the D4 map (PDB: 8Q04). **a**, 2D classes after manual picking of 465 particles, used for autopicking from all grids. **b**, Representative micrograph shown without (left) and with (right) autopicked particles (green circles). Autopicking resulted in 237,035 particles that were used in subsequent 2D classification. **c**, Selected 2D classes after classification, resulting in 224,593 particles that were used for 3D classification. **d**, Top view of the five 3D classes following classification, shown at the same contour level (0.01). Class 3 was used for subsequent refinements to create the final D4 map. Arrows indicate the regions of additional low resolution density at the equator of Rubisco. **e**, Post-processed map following refinement of class 3 with C1 symmetry imposed during refinement. **f**, Post-processed map following refinement with D4 symmetry. Maps in e and f are shown at a contour level of 0.032. **g**, Post-processed map following CTF refinement and Bayesian polishing with D4 symmetry imposed during refinement. The map is displayed at a contour level of 0.0553. **h**, Phenix local resolution estimate for the D4 CsRubisco map. **i**, Example density of residues 238–245 of the CsRbcL with the corresponding model coordinates, carved with a radius of 2 at a contour level of 0.0415. **j**, Overlay of cryo-EM structures of Rubisco from Chlorella solved in this study and from *Chlamydomonas reinhardtii* solved in a previous study^32^. **k**, Fourier shell correlation (FSC) curve showing the resolution estimate for the D4 refined map with FSC cut-off of 0.143 (dashed lines).

**Extended Data Fig. 5:**
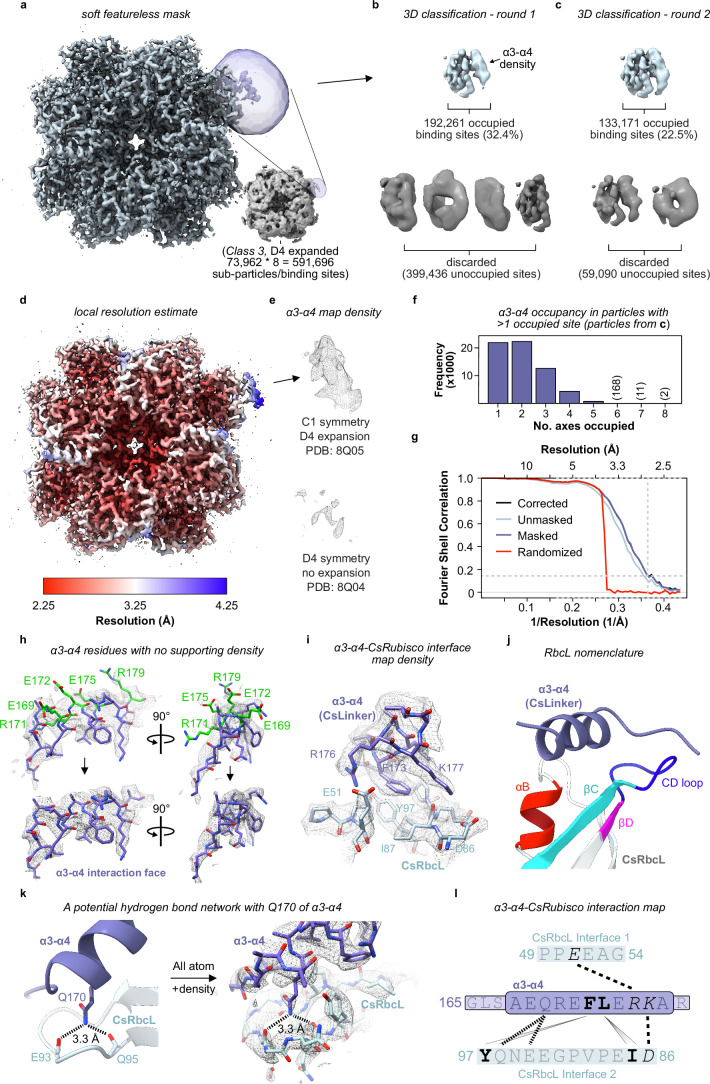
Cryo-EM data processing of the α3-α4-CsRubisco map (PDB: 8Q05). **a**, Sharpened (*B*-factor: 45) post-processed map of the C1 symmetry-refined α3-α4-CsRubisco complex using the D4 symmetry expanded particle dataset. The soft featureless mask used for the classification is shown in purple, over the region of additional density, into which the predicted helix of α3 is built. Shown at a contour level of 0.0496. The 3D class (class 3) from which the particle dataset was D4 symmetry expanded is shown inset, and the mask is schematically represented over a region of additional density. **b**, C1 symmetry 3D classification of sub-particles using the soft featureless mask. The selected class with α3-α4 density is shown in blue, with the discarded classes in grey below. At this resolution, the density shows clearly helical nature. **c**, Second round of 3D classification using the selected sub-particles from the first round. The selected sub-particles from this round were used for reconstruction of the α3-α4-CsRubisco map. **d**, Phenix local resolution estimate of the α3-α4-CsRubisco map, shown at a contour level of 0.0293. **e**, Map density of the α3-α4 region in the unsharpened, post-processed C1, symmetry expanded map shown at contour level 0.0293 (top), and the unsharpened, post-processed D4, non-symmetry expanded map, shown at a contour level of 0.0174. Both maps are carved with a radius of 2 around the modelled helical region. **f**, Histogram showing the distribution of α3-α4 occupancy in the sub-particles of the particles used for the reconstruction of the α3-α4-CsRubisco map. **g**, Fourier shell correlation (FSC) curve showing the resolution estimate for the α3-α4-CsRubisco map with FSC cut-off of 0.143 (dashed lines). **h**, Model of α3-α4 in the density displayed with the side chains of residues with no density support displayed in green (top) and removed in the final coordinates (bottom). **i**, Coordinates at the α3-α4-CsRubisco interface displayed in the map density at a contour level of 0.0304 and carved with a radius of 2. **j**, Nomenclature of RbcL regions at the α3-α4-CsRubisco interface. **k**, A potential hydrogen bond network between Gln170 of α3-α4 and Glu93 and Gln95 of the CsRbcL CD loop shown with and without map density shown at a contour level of 0.0396. **l**, α3-α4-CsRubisco interaction map.

To determine where CsLinker binds CsRubisco, we elucidated the 3D structure of the α3–α4–CsRubisco complex using cryogenic electron microscopy (cryo-EM) (Supplementary Table 13). We prepared samples of α3–α4 and CsRubisco such that the solution concentration of CsLinker repeats was comparable to the value we measured in the chloroplast (32 μM in this experiment, 37.4 μM in vivo; Fig. 2b). This concentration also saturated the α3–α4–CsRubisco complex (Fig. 3a). The 2.4 Å map and model we obtained with D4 reconstruction (PDB: 8Q04) was highly similar to a previously determined cryo-EM structure of *Chlamydomonas* Rubisco^32^ (Cα root mean square deviation (RMSD) of RbcL = 1.106 Å and RbcS = 0.937 Å, Extended Data Fig. 4). During processing, we observed a low-resolution density in addition to the core subunits at the equatorial region of CsRubisco in the RbcL (Extended Data Fig. 4d). We hypothesized that this additional density corresponded to the helical regions of α3–α4 and that substoichiometric binding meant many binding sites were unoccupied, giving rise to poorly resolved density where the linker binds. Using a symmetry expansion approach^35^, with a soft featureless mask around the additional density (Extended Data Fig. 5a), we found ~23% of the 8 CsRbcL sites to be occupied by additional density in the symmetry expanded subparticles (Extended Data Fig. 5c,f). By focusing on the subparticles bound by α3–α4, we obtained a map of the additional density which possessed a clearly helical nature (Fig. 3d and Extended Data Fig. 5e; PDB: 8Q05). We built the helical region of α3 into the density and numbered residues according to their position in the full-length protein (Figs. 1g and 3e and Extended Data Fig. 5h,i). Each CsLinker binding site is contained within the N-terminal region of a single CsRbcL subunit, utilizing two salt bridges on a hydrophobic interface (Fig. 3e,f and Supplementary Table 14). The two salt bridges are between Arg176 of α3–α4 and Glu51 of the CsRbcL, and between Lys177 of α3–α4 and Asp86 of the third beta-sheet (βC) in CsRbcL (Extended Data Fig. 5j). Phe173 of α3–α4 dominates the hydrophobic interaction in a pocket formed by Ile87 and Tyr97 of the CsRbcL. As the α3–α4 fragment contains a single residue difference between α3 and α4 (Fig. 1g), the residue at helix position 7 could be either a Leu (174) or Met (240). The map resolution did not allow distinction between the two, although when Leu174 was built into this density, it was also positioned to contribute to the hydrophobic interaction. We validated the binding interface by site-directed mutagenesis (SDM) of the α3–α4 fragment, selecting substitutions that disrupted the hydrophobic and salt-bridge interfaces. In both cases, disruption of α3–α4–CsRubisco complex formation by native PAGE was observed (Fig. 3g and Supplementary Fig. 12c).

### CsLinker complements pyrenoid formation in *Chlamydomonas*

**Fig. 4:**
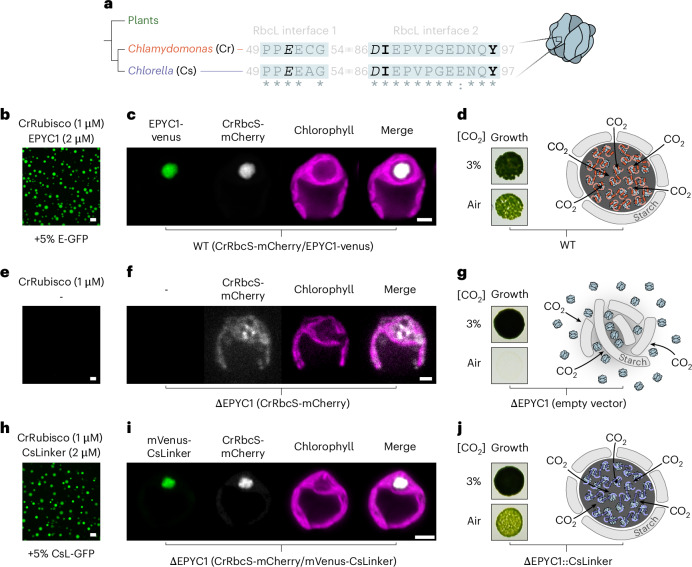
CsLinker can functionally replace EPYC1 in the *Chlamydomonas* pyrenoid. **a**, Alignment of the CsLinker-binding interface sequences from Chlorella (Cs) RbcL and the equivalent region of the *Chlamydomonas* (Cr) RbcL. Interacting residues are shown in black and stylized by interaction type according to Fig. 3f. Conserved residues are indicated by asterisks. **b**, Confocal fluorescence microscopy image of droplets formed with *Chlamydomonas* Rubisco (CrRubisco) and EPYC1, in which 5% (molar ratio) of the EPYC1 was GFP tagged (E-GFP). Scale bar, 5 μm. **c**, Confocal fluorescence microscopy image of WT *Chlamydomonas* (CC-4533) expressing EPYC1-Venus and CrRbcS-mCherry. Scale bar, 2 μm. **d**, Left: growth phenotype of WT *Chlamydomonas* grown on TP minimal media under elevated (3%) and ambient (Air) levels of CO_2_. Right: schematic representation of the pyrenoid. **e**, Confocal fluorescence microscopy image of CrRubisco alone. Scale bar, 5 μm. **f**, Confocal fluorescence microscopy image of ΔEPYC1 *Chlamydomonas* strain expressing CrRbcS-mCherry. Scale bar, 2 μm. **g**, Left: growth phenotype of ΔEPYC1 *Chlamydomonas* strain. Right: schematic representation of pyrenoid region. **h**, Droplets formed with *Chlamydomonas* Rubisco (CrRubisco) and CsLinker, in which 5% (molar ratio) of the CsLinker was GFP tagged (CsL-GFP). Scale bar, 5 μm. **i**, Confocal fluorescence microscopy image of ΔEPYC1 *Chlamydomonas* strain expressing CrRbcS-mCherry and mVenus-CsLinker. Scale bar, 2 μm. **j**, Left: growth phenotype of ΔEPYC1 *Chlamydomonas* strain complemented with untagged CsLinker. Right: schematic representation of pyrenoid. Results in **b** and **h** were observed on multiple independent occasions (see Extended Data Fig. 8), as were results in **c**, **f** and **i** (see Extended Data Fig. 6 and Supplementary Fig. 19). The result in **e** was from a single, non-repeated observation.

The RbcL is highly conserved across the green lineage^36^, in contrast to the small subunit (RbcS) which shows much higher sequence variation^37^ (Supplementary Fig. 13). Given the interaction of CsLinker with the RbcL (Fig. 3c), we wondered whether this affords CsLinker increased cross-reactivity for other Rubiscos relative to EPYC1, which binds the RbcS^32,38^. We first considered cross-reactivity of CsLinker between Chlorella and *Chlamydomonas* Rubiscos, where the CsLinker interaction interfaces are almost totally conserved (Fig. 4a). CsLinker phase separated *Chlamydomonas* Rubisco (CrRubisco) with a similar efficiency as its cognate CsRubisco (Fig. 4h) and with a similar efficiency as EPYC1 for its cognate CrRubisco (Fig. 4b and Extended Data Fig. 8a,d). By contrast, EPYC1 was unable to demix CsRubisco in the reciprocal experiment (Extended Data Fig. 8c,d), in line with the lack of conservation of EPYC1-interacting residues in CsRbcS (Supplementary Fig. 13). SPR experiments with α3 and α3–α4 showed comparable *K*_D_ values for CrRubisco as the cognate interaction (108 μM, CI_95_: 102–115 μM; and 1.30 μM, CI_95_: 1.16–1.46 μM, respectively; Supplementary Fig. 11). A similar α3–α4–CrRubisco complex was also observed by native PAGE, albeit with apparently less stability (Supplementary Fig. 14c). Together, these data are consistent with CsLinker binding the same conserved interface in both Rubiscos.

**Extended Data Fig. 6:**
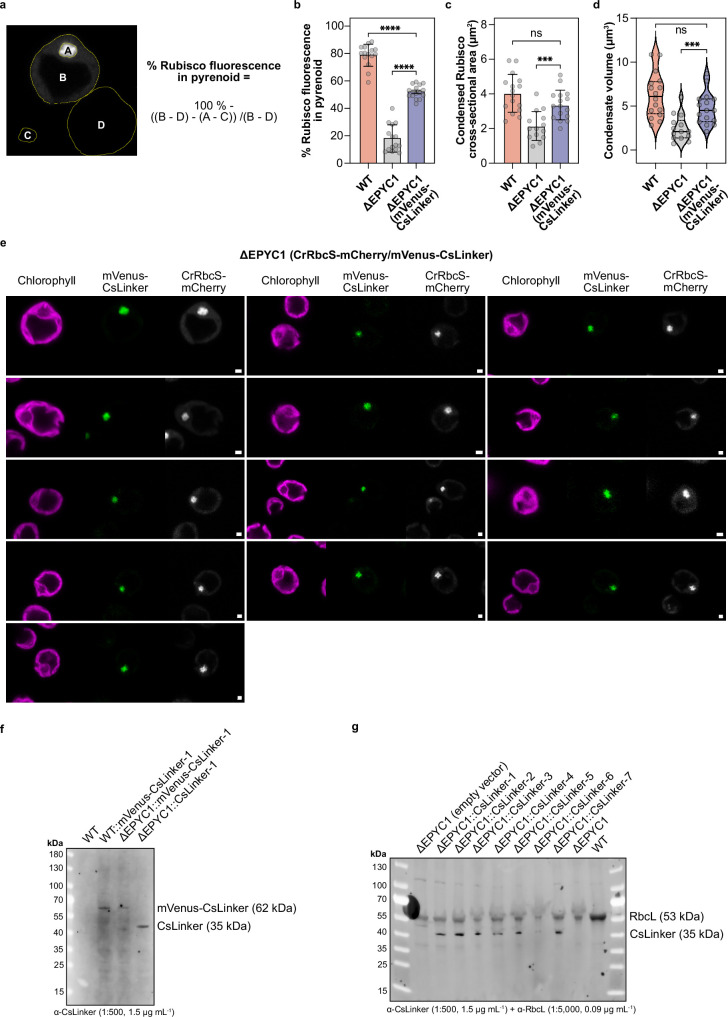
CsLinker expression recovers pyrenoid formation in ΔEPYC1 *Chlamydomonas.* **a**, Example of Rubisco partitioning calculation using integrated density analysis in Fiji, according to ref. ^15^. **b**, Rubisco partitioning in the condensate in the WT (CrRbcS-mCherry/EPYC1-Venus), ΔEPYC1 (CrRbcS-mCherry) and ΔEPYC1 (CrRbcS-mCherry/CsLinker) lines, quantified from the images in e and Supplementary Fig. 15 using the method outlined in a. Statistical significance from unpaired t-tests are indicated; **** = p < 0.0001. **c**, Area of the Rubisco condensate as measured in Fiji, according to region ‘A’ in a. For the ΔEPYC1 (CrRbcS-mCherry), the largest condensed fluorescence signal at the canonical position was measured. *** = p < 0.001. The median and quartile values are represented by the solid and dashed horizontal lines in each plot. **d**, Estimated volume distributions of the Rubisco condensates in the three lines, assuming sphericity of the condensate and calculating from the cross-sectional area in b. *** = p < 0.001. **e**, Confocal fluorescence microscopy images of tagged RbcS and CsLinker in the ΔEPYC1 background. Scale bars = 1 μm. **f**, Western blot confirmation of CsLinker variant expression in WT and ΔEPYC1 background lines. **g**, Western blot confirmation of CsLinker expression in ΔEPYC1 background relative to the empty vector and background strains. RbcL was used as a loading control.

**Extended Data Fig. 7:**
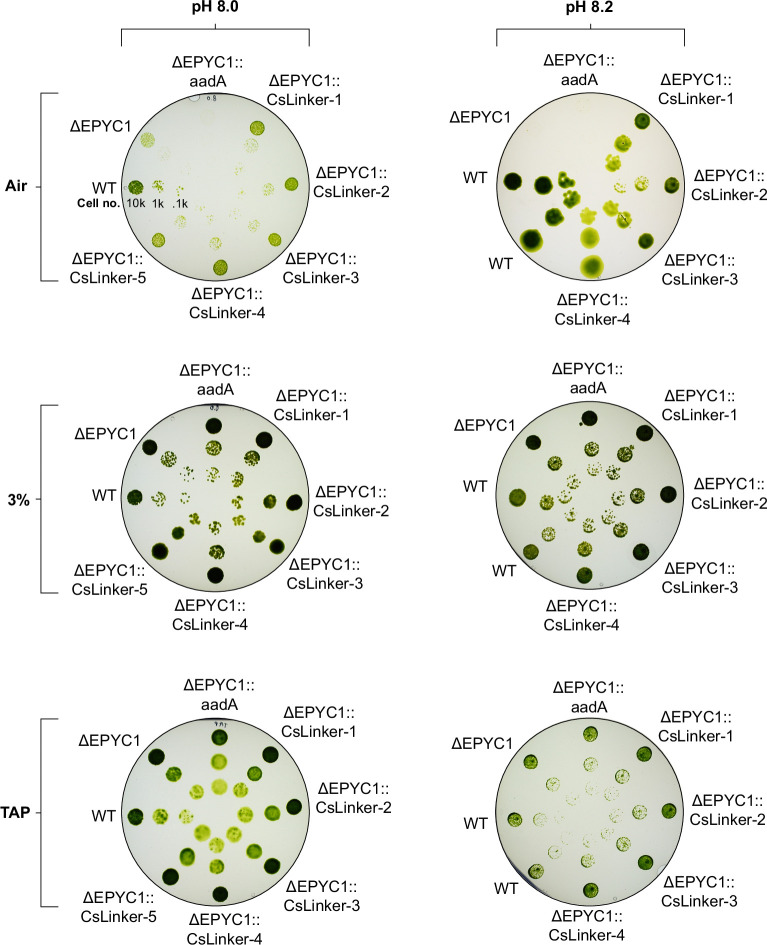
Spot test of CsLinker replacement lines. Images of spot test plates following 5 days of growth at the indicated conditions. Images used in Fig. 4 were taken from the pH 8.0 dataset.

LLPS of Rubisco in the *Chlamydomonas* pyrenoid is essential to its function, allowing growth at atmospheric CO_2_ levels^15^ (Fig. 4d,g). Given the in vitro cross-reactivity of CsLinker and the functional analogy with EPYC1, we next considered whether CsLinker could replace EPYC1 in the pyrenoid of *Chlamydomonas*. We utilized a previously characterized *Chlamydomonas* strain that lacks EPYC1 (ΔEPYC1) and accordingly does not form pyrenoids. This strain has Rubisco distributed throughout the chloroplast and exhibits reduced growth under ambient CO_2_ conditions (air)^15^ (Fig. 4f,g). We expressed mVenus-tagged CsLinker in the chloroplast of ΔEPYC1 and subsequently co-expressed mCherry-tagged CrRbcS in the resulting strain to create a ΔEPYC1 (CrRbcS-mCherry/mVenus-CsLinker) line. In line with our observations in vitro (Fig. 4h), mVenus-CsLinker expression led to the formation of a micron-scale condensate at the canonical pyrenoid position that contained both CrRbcS and CsLinker (Fig. 4i). Although Rubisco partitioning and condensate size was reduced compared with wild type (WT), it was significantly increased over the background ΔEPYC1 strain, suggesting in vivo condensation of CrRubisco by CsLinker (Extended Data Fig. 6). To avoid any phenotypic impact of tagging either CsLinker or CrRubisco, we expressed untagged CsLinker in the ΔEPYC1 background and confirmed expression by western blotting (Extended Data Fig. 6g). In line with the visual recovery of Rubisco condensation by mVenus-CsLinker, introduction of untagged CsLinker restored the growth of the resulting ΔEPYC1::CsLinker strain in air to almost wild-type levels (Fig. 4j and Extended Data Fig. 7). The functional complementation of EPYC1 by CsLinker presents a compelling example of functional LLPS-driven organelle assembly complementation by a protein with little sequence similarity and a different binding interface, across a ~800 Myr evolutionary gap.

### CsLinker condenses plant Rubisco in vitro and in planta

**Fig. 5:**
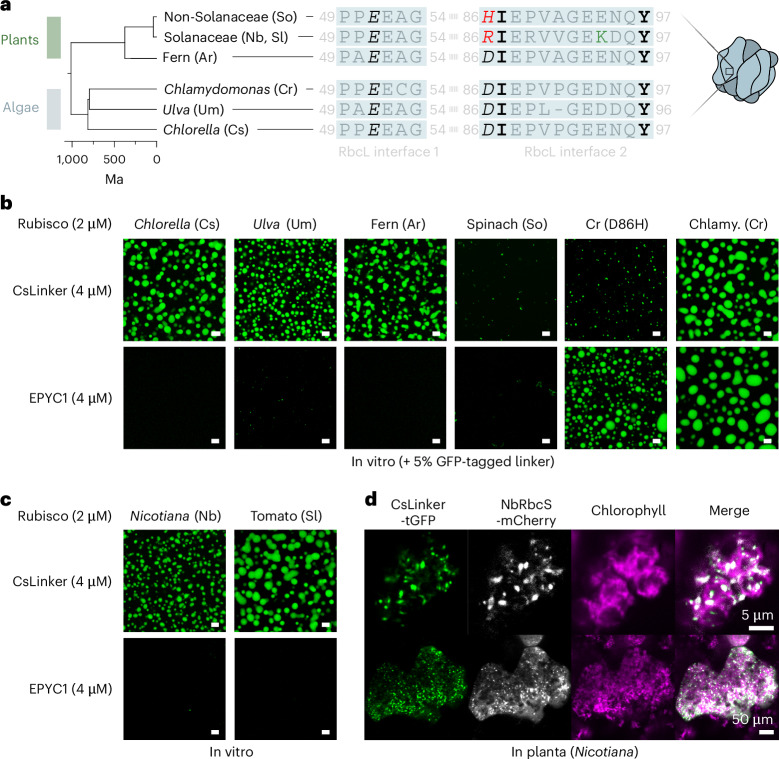
CsLinker condenses native plant Rubisco in vitro and in planta*.* **a**, Alignment of the CsLinker-binding interface sequences from algal and plant RbcLs. Interacting residues are shown in black and stylized by interaction type according to Fig. 3f. Substitutions of interacting residues are shown in red. **b**, Confocal fluorescence microscopy images of droplets formed with different Rubiscos and either CsLinker or EPYC1. **c**, Images of droplets formed with Solanaceae Rubisco and either CsLinker or EPYC1. Scale bars in **b** and **c**, 5 μm. Results in **b** and **c** were from single, non-repeated observations. **d**, Confocal fluorescence microscopy images of CsLinker-tGFP and NbRbcS-mCherry transiently expressed in *N. benthamiana* chloroplasts, condensed into Rubisco puncta in planta. Results in **d** were from multiple repeated observations (see Extended Data Fig. 10).

**Extended Data Fig. 8:**
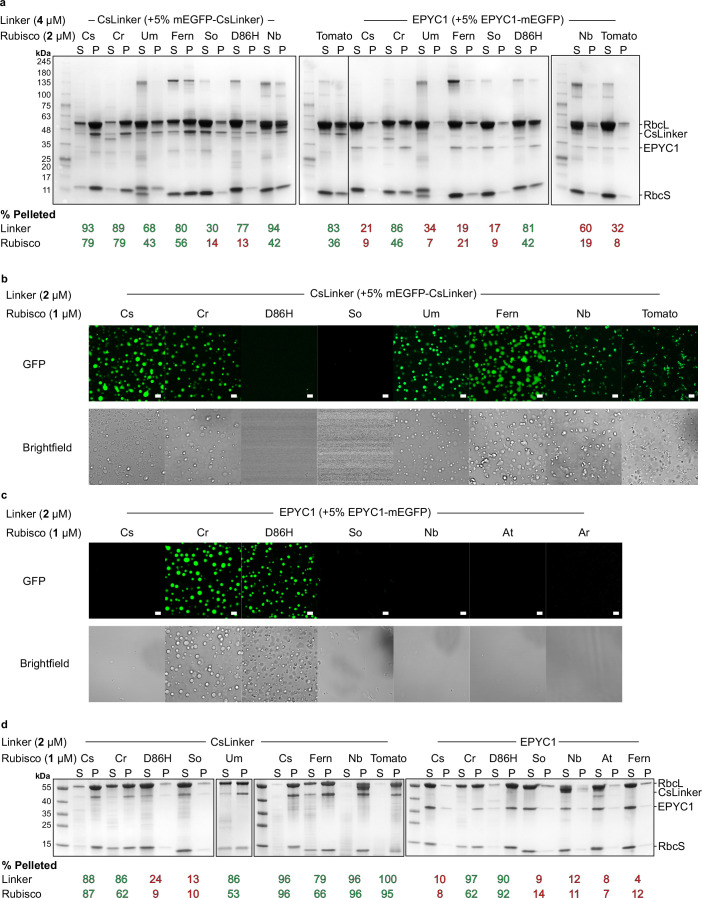
Cross-reactivity of CsLinker with green lineage Rubiscos. **a**, Droplet sedimentation assays comparing the cross-reactivity of CsLinker and EPYC1 fixed at 4 μM and green Rubiscos fixed at 2 μM. In these experiments, tagged linker was also included at 5% molar ratio as completed in the accompanying microscopy experiments in Fig. 5. The amount of Rubisco and Linker pelleted in each reaction is indicated below, with the numbers colored green if droplet formation was observed in the accompanying microscopy experiments in Fig. 5. **b**, Confocal fluorescence microscopy and brightfield images of droplets formed with CsLinker fixed at 2 μM, with Rubiscos from the green lineage fixed at 1 μM. mEGFP-CsLinker was included at a 5% molar ratio. **c**, Images of droplets formed with EPYC1 fixed at 2 μM ( + 5% EPYC1-mEGFP molar ratio), with Rubiscos from the green lineage fixed at 1 μM. Scale bar in b and c is 5 μm. **d**, Droplet sedimentation assays comparing the cross-reactivity of CsLinker and EPYC1 fixed at 2 μM, with Rubiscos from the green lineage fixed at 1 μM. The amount of Rubisco and Linker pelleted in each reaction is indicated below, with the numbers colored green if droplet formation was observed in the accompanying microscopy experiments in b and c. Abbreviations: Cs = *Chlorella sorokiniana*, Cr = *Chlamydomonas reinhardtii*, Um = *Ulva mutabilis*, Ar = *Adiantum raddianum* (Fern), So = *Spinacia oleracea*, Nb = *Nicotiana benthamiana*, D86H = *Chlamydomonas reinhardtii* with D86H mutation made in RbcL.

**Extended Data Fig. 9:**
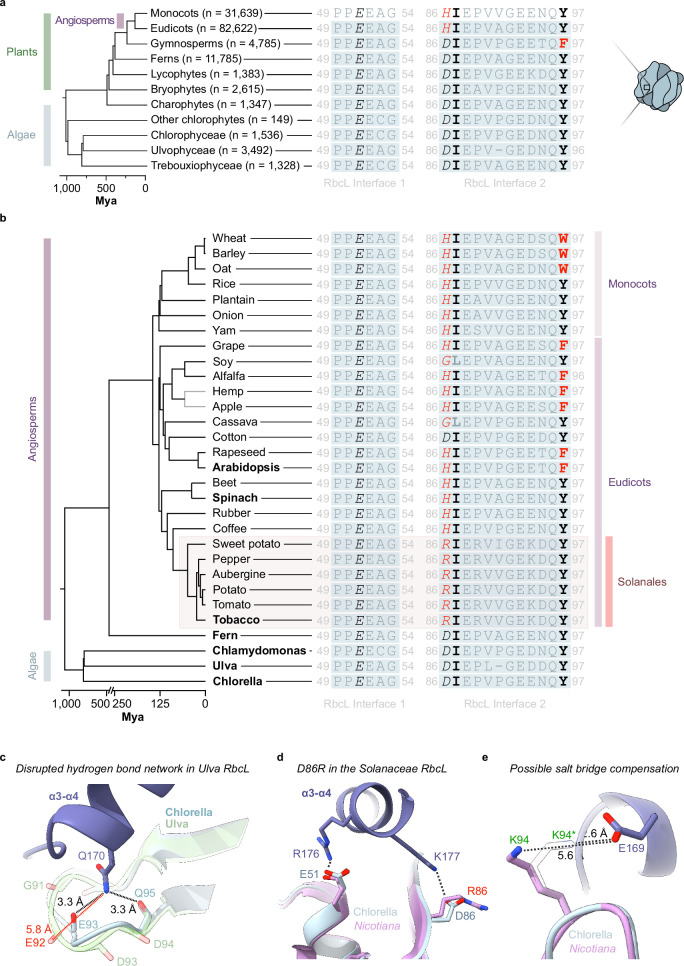
Variation of the CsLinker-interacting interface in plant RbcLs and potential alternative interactions at the interface. **a**, Alignment of the consensus sequences of the CsLinker-binding interface in the RbcLs of major plant groups. Consensus sequences were produced from alignment of the indicated number of sequences in each class from NCBI. **b**, Alignment of the CsLinker-binding interfaces in the RbcLs of the 24 most valuable C_3_ crop plants (FAOSTAT data) alongside the algal and fern Rubiscos tested in this study. **c**, The possibly disrupted hydrogen bond network in the βC-D loop of the *Ulva* RbcL. The AlphaFold 2 structural prediction of the *Ulva* RbcL (green) is shown aligned with the Chlorella RbcL coordinates solved in this study (blue). The disrupted hydrogen bond is annotated in red, with the corresponding lengths of the cognate and disrupted hydrogen bonds shown in red and black respectively. **d**, Demonstration of the disrupted salt bridge in the *Nicotiana* RbcL^40^ due to the D86R substitution. **e**, A possible compensatory salt bridge in the K94 residue of the βC-D loop in the *Nicotiana* RbcL if an alternative residue conformer is occupied (K94*).

Encouraged by the functional cross-reactivity of CsLinker we observed with *Chlamydomonas* Rubisco, we sought to understand the extent of cross-reactivity for Rubiscos in the green lineage. We next demonstrated that CsLinker was able to demix Rubisco from the multicellular ulvophyte seaweed, *Ulva mutabilis* (Um), which retains all four CsLinker-interacting residues (Fig. 5a). Notably, the efficiency of phase separation was lower (Extended Data Fig. 8), SPR experiments demonstrated higher *K*_D_ values of the α3 and α3–α4 fragments for UmRubisco (162 μM, CI_95_: 158–167 μM; and 1.56 μM, CI_95_: 1.40–1.72 μM, respectively; Supplementary Fig. 11), and native PAGE assays showed little gel shift (Supplementary Fig. 14f). We attribute the reduced affinity and concomitant phase separation efficiency to the reduction of the *Ulva* RbcL interface 2 by one residue (Fig. 5a). This change would disrupt a potential hydrogen bond network with the Gln residue of the CsLinker helices, although the resolution in this region of the α3–α4–CsRubisco complex map did not allow distinctive assignment of this network (Extended Data Figs. 5k and 9c).

Motivated by the wider goal of engineering pyrenoids in C_3_ angiosperm crop plants to enhance CO_2_ fixation^18,19^, we next wondered whether the cross-reactivity of CsLinker extends to plant Rubiscos. We analysed RbcL sequences from major plant groups for conservation of CsLinker-interacting residues and found that early diverged, non-flowering ferns were phylogenetically the closest plant group to angiosperms to contain all four key residues (Fig. 5a and Extended Data Fig. 9a). Accordingly, Rubisco from the fern *Adiantum raddianum* (Ar) demonstrated phase separation with CsLinker in vitro (Fig. 5b), with a similar efficiency to the cognate pairing (Extended Data Fig. 8). Most angiosperm crops have a substitution of Asp86 for His86 in their RbcLs (Extended Data Fig. 9b). We tested the effect of this substitution (D86H) using Rubisco purified from spinach (*Spinacia oleracea*; So), which has RbcL interfaces representative of the consensus angiosperm sequence (Extended Data Fig. 9a,b). We observed submicron aggregates of spinach Rubisco at the same concentrations used for the cognate CsLinker interaction, but never observed droplet formation (Fig. 5b and Extended Data Fig. 8). To confirm that this effect was predominated by the D86H substitution, we made the same substitution in the RbcL of CrRubisco that we previously demonstrated phase separates with CsLinker (Figs. 4h and 5b). The D86H mutation disrupted droplet formation of CrRubisco, which instead demonstrated similar behaviour to the spinach Rubisco (Fig. 5b). Notably, the D86H mutated CrRubisco still phase separated with its cognate linker EPYC1 (Fig. 5b). Complex formation of D86H CrRubisco with α3–α4 was also disrupted, as assessed by native PAGE (Supplementary Fig. 14i). These data suggest that substitution of the D86 residue severely impacts the affinity and phase-separation propensity of CsLinker for Rubiscos of most angiosperm plants which possess a His in this position, including most of the key C_3_ crop plants (rice, wheat, soybean) (Extended Data Fig. 9b).

**Extended Data Fig. 10:**
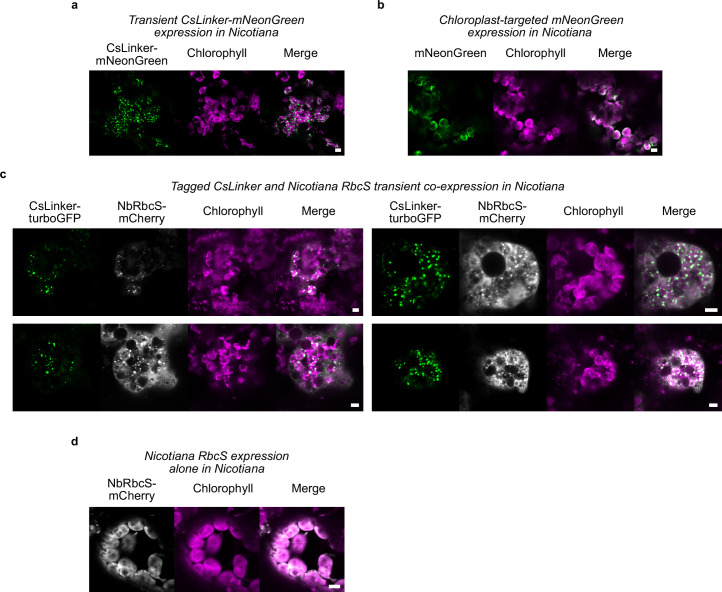
Transient expression of CsLinker in *Nicotiana benthamiana.* **a**, Confocal microscopy image of turboGFP-tagged CsLinker transiently expressed alone in *Nicotiana*. **b**, Image of chloroplast-targeted (fused to *Arabidopsis* RbcS signal peptide; SP1A) turboGFP expressed alone, demonstrating a lack of puncta. **c**, Images of transient co-expression of CsLinker-turboGFP and mCherry-tagged *Nicotiana benthamiana* RbcS. **d**, Image of NbRbcS-mCherry transiently expressed alone in *Nicotiana* chloroplasts. Scale bars in a-d = 5 μm. The results presented in panels **a**, **b** and **d** were from single non-repeated experiments. The results in panel **c** (and Fig. 5d) were from two independent experiments.

Among angiosperm RbcL sequences, there is some variation in the sequences of the CsLinker-interacting interfaces (Extended Data Fig. 9b). RbcLs of the nightshade family (Solanaceae), which contains some of the most widely consumed plants (potato, tomato, eggplant, pepper, tobacco), possess a distinct sequence composition at interface 2 due to co-evolution with their specific Rubisco activase which also binds this region^39^. Solanaceae RbcLs possess an Arg at position 86 while retaining the other three key interacting residues, but also display other unique sequence features in interface 2 (Fig. 5a). Given the charge inversion at position 86 (D86R) and presumed subsequent salt-bridge disruption (Fig. 3e), we predicted that Solanaceae Rubiscos would not phase separate with CsLinker. Surprisingly, Rubisco from the model Solanaceous plant *Nicotiana benthamiana* (NbRubisco; tobacco) was readily phase separated in vitro (Fig. 5c). As this behaviour was specific to CsLinker (and not EPYC1), we hypothesized that the interaction between CsLinker and NbRbcL is based on a similar binding interface as CsRbcL. Analysis of the Rubisco structure from *Nicotiana tabacum* (PDB: 1EJ7 (ref. ^40^)) indicated that Lys94 of the RbcL (green in Fig. 5a) is favourably positioned to form a salt bridge with Glu169 of the helix of CsLinker (Extended Data Fig. 9e), which could compensate for the loss of the D86 salt bridge. Given that all key nightshade plants have conserved CsLinker-interacting interfaces (Extended Data Fig. 9b), we were interested whether CsLinker could phase separate Rubisco from other widely consumed members of this family. In line with the results of *Nicotiana*, tomato Rubisco (*Solanum lycopersicum*) readily demixed with CsLinker in vitro (Fig. 5c and Extended Data Fig. 8). Finally, to explore whether the in vitro observation of Solanaceae Rubisco condensation extended in planta, we transiently co-expressed TurboGFP-tagged CsLinker and mCherry-tagged NbRbcS in *N. benthamiana*. Strikingly, micron-scale condensates were observed in each chloroplast, which contained both Rubisco and CsLinker, representing presumably the first phase separation of native plant Rubisco in planta (Fig. 5d and Extended Data Fig. 10). The condensation of native plant Rubisco represents a significant step forward in the goal of engineering pyrenoids in plants and provides prospect that ongoing engineering of CsLinker could allow phase separation of native non-Solanaceae crop Rubiscos.

## Discussion

Our in-depth characterization of a pyrenoid linker protein from Chlorella has yielded significant insight into LLPS-driven organelle assembly and provided exciting frontiers for future plant pyrenoid engineering approaches to enhance photosynthesis.

Identification that CsLinker binds to the RbcL and structural characterization of the RbcL binding site has allowed us to make three general observations. First, green lineage pyrenoid linker proteins probably evolved separately and convergently, as previously proposed^15,32,41^, and their binding region was not constrained to the small subunit of Rubisco. Their different sequence composition, binding affinity and binding region suggests that the general physical properties of linkers also probably converged at linker-specific optima, which is supported by ongoing modelling efforts^42,43^. Second, given that Chlorella and *Chlamydomonas* diverged ~800 Ma, the capacity to functionally exchange EPYC1 for CsLinker in the *Chlamydomonas* pyrenoid provides a compelling example that the conservation of functionally critical physicochemical properties in intrinsically disordered proteins can be more important than conservation of a specific primary sequence. This example showcases the algal pyrenoid as a tractable model to explore the evolution of biomolecular condensation and LLPS evolution in vitro and in vivo with clear biological fitness readouts—often a limitation in LLPS systems^31^. We anticipate that our work will enable future studies to systematically test how the physical properties of phase-separating proteins (for example, sticker number, sticker binding affinity, spacer length and spacer flexibility^44^) impact biological fitness. Third, the high conservation of the CsLinker binding site in the green lineage RbcLs enabled cross-reactivity of CsLinker with plant Rubiscos and allowed us to demonstrate phase separation of unmodified plant Rubisco presumably for the first time. This finding overcomes a major future barrier for pyrenoid engineering in plants, which is currently dependent on genetic replacement of the multiple host plant RbcS proteins with those of *Chlamydomonas*^23,24,45^.

While our results are encouraging, we acknowledge several areas that will require future work to address. First, we presume that our demonstration of EPYC1 replacement in *Chlamydomonas* is dependent on retention of the native RbcS which is responsible for organizing other essential pyrenoid features through interaction with Rubisco-binding motif (RBM)-containing proteins^21,46^. Future approaches to engineer functional pyrenoids in plants without Rubisco engineering will be dependent on the development of approaches that circumvent RbcS interaction entirely, either by replacing RBMs in *Chlamydomonas* proteins or by identifying and characterizing analogous RbcL-binding parts, possibly from Chlorella. Second, although in vivo expression of CsLinker in both *Chlamydomonas* and *Nicotiana* resulted in Rubisco condensation, the partitioning of Rubisco in the condensate was lower than in WT and multiple condensates were observed in planta. While the level of CsLinker expression probably plays an important role, these observations also suggest a degree of tunability in the phase separation of Rubisco that is dependent on specific features of the interaction proteins when the interface is not fully conserved. Future studies could probably exploit this tunability to expand the cross-functionality of CsLinker with other non-Solanaceae Rubiscos, making use of the molecular details of the interaction outlined here.

Moving forward, as additional pyrenoids and their corresponding assembly proteins from diverse algae are characterized, we expect our understanding of their evolution, the underlying principles of pyrenoid assembly and our ability to model pyrenoid systems to rapidly advance. We envision that this knowledge will provide an expanded parts list for pyrenoids and give us new tools to predict and modulate pyrenoid properties and thereby accelerate future efforts to engineer pyrenoids in plants.

## Methods

### Strains and culture conditions

*Chlorella sorokiniana* UTEX1230 (SAG 211-8k), *Chlamydomonas reinhardtii* WT (CC-4533), ΔEPYC1 (CC-5360) and resulting strains were maintained on 1.5% agar Tris-acetate-phosphate (TAP) medium with revised trace elements^47^ plates in low light (~10 μmol photons m^−2^ s^−1^). For Rubisco extraction, growth, immunoelectron microscopy and confocal microscopy experiments, strains were grown in Erlenmeyer flasks in Tris-phosphate (TP) media in medium light (~50 μmol photons m^−2^ s^−1^) under ambient CO_2_ to a density of ~0.5–1 × 10^7^ cells per ml.

### FLIPPer and bioinformatic analysis

FLIPPer was originally built using IUPred2A^48^ as the disorder prediction software, which was used in the initial identification of CsLinker. For licensing reasons, the pipeline was rebuilt using metapredict V2 (ref. ^49^); the outputs were largely unchanged. FLIPPer is available in GitHub^50^ and was used with default settings thresholded on the basis of EPYC1 sequence analysis. Briefly, input sequences were physicochemically filtered, repeats detected with XSTREAM^51^ and filtered to contain interacting residues before disorder prediction and filtering with metapredict. Output sequence structures were manually examined for repeated helical regions by AlphaFold 2 (ref. ^52^) prediction in ColabFold (v.1.5)^53^. Differential gene expression analysis was completed using Salmon^54^ according to ref. ^55^, using publicly available data (PRJNA343632). BLAST analysis was completed against the non-redundant (nr) sequence database using the NCBI web tool, with an expect threshold of 0.05 without filtering low complexity regions. Statistical analyses were completed using Prism 10. Multiple alignment using fast Fourier transform (MAFFT) was used for sequence alignments^56^.

### Co-immunoprecipitation mass spectrometry

Polyclonal rabbit antibodies were raised to Rubisco (EVWKEIKFEFETIDTL-cooh) and CsLinker (PTPVSNSGVRSAMSSG-amide) peptides (YenZym Antibodies). The control rabbit antibody was raised to *Chlamydomonas* BST2 (PDLDSINAAAPNGNGSHNGN-amide). Chlorella cells (1 × 10^9^) grown in TP medium sparged with 0.01% CO_2_ were lysed by ultrasonication in 10 ml IP buffer (20 mM Tris-HCl pH 8.0, 50 mM NaCl, 0.1 mM EDTA, 12.5% glycerol (w/v), 5 mM dithiothreitol, 1× cOmplete protease inhibitor tablet per 50 ml). A volume of 1 ml of clarified lysate (30 min at 50,000 *g*) was applied to 200 μl of protein A Dynabeads (Invitrogen) loaded with 32 μg of respective antibodies that had been blocked with bovine serum albumin (BSA) (2 mg ml^−1^) for 1 h and washed 3 times with IP buffer. The lysate was incubated for 3 h before washing. Bound protein was trypsin digested from the beads, post reduced with dithioerythritol (DTE) and alkylated with iodoacetamide before UPLC separation by the 60SPD EvoSep One (EvoSep) method and acquisition by PASEF-DIA method using a timsTOF HT mass spectrometer (Bruker). Data were searched using DIA-NN^57^ and filtered to 1% false discovery rate (FDR) with a minimum of two unique peptides. FragPipe-Analyst^58^ was used for differential abundance testing.

### Immunoelectron microscopy

Cells were fixed in 1% glutaraldehyde, 2% formaldehyde in 0.1 M sodium cacodylate (pH 7.4) for 1 h before resuspension in 2% (w/v) agar. Agar blocks were dehydrated using a 10–50% ethanol gradient at 4 °C, followed by 70–100% at −20 °C, then infiltrated with LR White resin containing 0.5% benzoin methyl ether (London Resin) and polymerized in gelatin capsules for 24 h at −20 °C and 24 h at −10 °C under UV light. Sections (70 nm) were cut using a Leica UCT7 ultramicrotome with a Diatome knife and mounted on nickel grids. All imaging was completed using an FEI Tecnai T12 BioTWIN transmission electron microscope (TEM) operating at 120 kV with Ceta CCD camera.

For immunolabelling, grids were blocked with 3% BSA (w/v) in PBS for 30 min before primary incubation with either purified antibody (0.09 μg ml^−1^) or pre-immune serum (1:5,000 dilution) for 1 h at 30 °C in a humidity chamber. Secondary incubation was completed with a 1:40 dilution of goat anti-rabbit IgG 10 nm gold conjugate for a further hour (Merck).

### Absolute quantification mass spectrometry

Chlorella cells (6 × 10^6^) grown in TP medium sparged with 0.01% CO_2_ were boiled in 50 μl of Laemmli buffer in triplicate for the biological samples. The standards were separately boiled in duplicate in the same volume. Both the samples and standards were trypsin digested from SDS–PAGE gels, post reduced with DTE, and alkylated with iodoacetamide before separation by UPLC with a 25 cm PepMap column (ThermoFisher) and 1 h data-dependent acquisition using an Orbitrap Fusion Lumos Tribrid mass spectrometer (ThermoFisher). Technical replicate injections were used for the biological samples, after which the values were averaged. LC–MS chromatograms were aligned using Progenesis QI and the MS2 spectrum was searched using Mascot with a 1% FDR. Matches were mapped to MS1 intensity using Progenesis QI and summed at protein level. The external calibration curve was used to calculate protein abundance in the biological samples (Supplementary Fig. 6).

### Cloning, plasmids and strains

For *E. coli*, the mature CsLinker sequence was predicted by TargetP (2.0)^59^, codon optimized in Geneious Prime using the *E. coli* K-12 codon usage table and synthesized (TWIST Bioscience). The sequence was ligation-independent cloned (LIC) into pET His6 GFP TEV LIC cloning vector (Addgene, 29663) to produce the His-mEGFP-TEV-CsLinker plasmid. The mEGFP-CsLinker fusion was subsequently PCR amplified and LIC cloned into pET MBP His6 LIC cloning vector (Addgene, 37237) to produce His-mEGFP-TEV-CsLinker-TEV-MBP-His plasmid. α3 (residues 142–207) and α3–α4 (residues 142–272) fragments were PCR amplified from this plasmid and Gibson assembled back into the PCR-amplified backbone to produce His-mEGFP-TEV-α3-TEV-MBP-His and His-mEGFP-TEV-α3–α4-TEV-MBP-His, respectively. SDM constructs were constructed by introducing nucleotide exchanges in the primers used to amplify the α3–α4 fragment from His-mEGFP-TEV-α3–α4-TEV-MBP-His. The SDM sequences were Gibson assembled back into the backbone to create His-mEGFP-TEV-α3(F173DL174D)-α4(F239DM240D)-TEV-MBP-His and His-mEGFP-TEV-α3(E169AR176A)-α4(E235AR242A)-TEV-MBP-His constructs.

For *Chlamydomonas* chloroplast expression, the mature CsLinker was codon optimized in Geneious Prime using the *Chlamydomonas* chloroplast codon usage table (Kazusa) and synthesized (TWIST Bioscience). The mVenus sequence was codon optimized using Codon Usage Optimizer v.0.92 (https://github.com/khai-/CUO). Sequences were PCR amplified and golden gate assembled into pME_Cp_2_098 (gift from René Inckemann and Tobias Erb). The ΔRbcL plasmid was created by Gibson assembly of the aadA gene amplified from CC-5168 into plasmid P-67 cpDNA EcoRI 14 (Chlamydomonas Resource Centre) to replace the RbcL CDS in frame. The RbcL_D86H plasmid was created by KLD site-directed mutagenesis (New England Biolabs) of the P-67 cpDNA EcoRI 14 plasmid (Chlamydomonas Resource Centre).

For *N. benthamiana* transient expression, the mature CsLinker sequence was codon optimized and synthesized using GeneArt and the *N. benthamiana* codon usage table (ThermoFisher). The sequence was golden gate assembled into pICH47732 with a 35S promoter, AtRbcS1A transit peptide, C-terminal Turbo-GFP and HSP + NOS double terminator according to ref. ^60^. The NbRbcS was synthesized with AtRbcS1A transit peptide by IDT and assembled into pICH47751 with AtRbcS1A promoter, C-terminal mCherry tag and OCS terminator.

Amino acid sequences of proteins and peptides used are shown in Supplementary Table 15.

### Rubisco extraction

Rubisco was purified from *Chlamydomonas* and Chlorella according to ref. ^61^, with the addition of a 16.5 h, 37,000 r.p.m. 10–30% sucrose gradient ultra-centrifugation step performed in an SW41-Ti rotor before anion exchange using a HiTrap 5 ml Q XL column (Cytiva). The same method was used for *Ulva*, spinach, *Adiantum*, *Nicotiana* and tomato, except that lysis was completed by manual agitation in a blender. Rubisco was labelled using an Atto594 protein labelling kit (Sigma-Aldrich), according to manufacturer instructions.

### *E. coli* protein purification

All constructs were purified from *E. coli* BL21 (DE3) strains harbouring respective plasmids. Cells were grown to optical density at 600 nm (OD_600_) of 0.5–0.8 in Luria Broth or ^15^NH_4_Cl minimal media for the NMR samples, before induction with 1 mM isopropyl *β*-d-1-thiogalactopyranoside (IPTG) for 3 h at 37 °C. Pellets were snap frozen before ultrasonic lysis in high-salt buffer (50 mM Tris-HCl, 500 mM NaCl, 25 mM imidazole, pH 8.0) with 2 mM phenylmethanesulfonylfluoride (PMSF). Soluble protein was applied to an IMAC column (HisTrap FF Crude 5 ml, Cytiva), washed with high-salt buffer and eluted with a linear gradient to 500 mM imidazole in high-salt buffer. For untagged CsLinker, α3, α3–α4, α3(F173DL174D)–α4(F239DM240D) and α3(E169AR176A)–α4(E235AR242A) constructs, the N-terminal mEGFP and C-terminal MBP were cleaved overnight with TEV protease produced according to ref. ^62^. The cleaved solution was passed over an IMAC column equilibrated with high-salt buffer and the untagged flow-through was collected. Size-exclusion chromatography (SEC) was completed on the flow-through using a HiLoad 16/600 Superdex 75 pg column (Cytiva) equilibrated with 50 mM Tris-HCl, 500 mM NaCl, pH 8.0 buffer. For the mEGFP-CsLinker, no TEV cleavage or second IMAC was completed, but protein was exposed to SEC.

### Western blotting

Following separation by SDS–PAGE, proteins were transferred to iBlot mini nitrocellulose membranes using the iBlot 2 system operated with method P0. Membranes were blocked in 5% milk Tris-buffered saline with Tween 20 (TBST) for 1 h at r.t. before incubation with the primary antibody at the indicated concentrations in 1% milk TBST overnight at 4 °C. Detection was completed using a Typhoon 5 scanner (Cytiva) following incubation with goat anti-rabbit Alexa Fluor 488 (A-11008, ThermoFisher) or anti-mouse Alexa Fluor 555 (A-21422, ThermoFisher) secondary antibodies for 1 h at 4 °C.

### Droplet sedimentation assay

Unless otherwise stated, all assays were completed in 5 μl reaction volumes in a buffer of low ionic strength (50 mM Tris-HClpH 8.0, 50 mM NaCl). Rubisco was added first in all cases, followed by linker and subsequent aspiration of the solution. Samples were incubated at room temperature for 15 min, followed by sedimentation at 10,000 *g* for 10 min before analysis by SDS–PAGE. Band intensity was quantified in Fiji^63^.

### Pyrenoid enrichment

Cells (5 × 10^9^) grown to exponential phase under ambient CO_2_ conditions in TP medium were washed in 30 mM HEPES-KOH (pH 8.0) and resuspended in 1 ml of 30 mM HEPES-KOH (pH 8.0) + 1% (w/v) formaldehyde at room temperature for 20 min. Fixing was quenched by addition of Tris-HCl (pH 8.0) to 1 M. Partially fixed cells were washed and resuspended in 1 ml pyrenoid enrichment buffer (50 mM Tris-HCl, 0.2 mM EDTA, 0.5 % (v/v) Triton X-100, pH 8.0) and lysed by sonication (3 min processing time, 3 s pulses at 30% amplitude using a micro-tip and Misonix S-4000 sonicator). Crude pyrenoid fractions were enriched by centrifugation at 2,500 *g* for 20 min, washed once in pyrenoid enrichment buffer and resuspended. The 1 ml crude pyrenoid fraction was centrifuged through a 9 ml Percoll cushion at 2,500 *g* for 15 min. The pelleted pyrenoid fraction was washed once in pyrenoid enrichment buffer.

Immunofluorescence of pyrenoid-enriched fractions was completed overnight in 1% BSA (w/v) TBST with CsLinker (1:50 dilution), RbcL (1:250) or tubulin (1:50; T6074, Sigma-Aldrich) primary antibodies at 4 °C. The fractions were washed with TBST twice before incubation with a 1:1,000 dilution of either goat anti-rabbit Alexa Fluor 488 or anti-mouse Alexa Fluor 555 secondary antibodies at r.t. for 1 h in 1% BSA (w/v) TBST. The fractions were washed before imaging.

### In vitro confocal microscopy and FRAP

All in vitro confocal and FRAP experiments were completed on either a Zeiss LSM880 or Zeiss LSM980 confocal microscope with a ×63, 1.4 numerical aperture (NA) Plan-Apo oil-immersion lens (Carl Zeiss) operated with ZEN blue or black software respectively. Reaction volumes (5 μl) were formed in µ-Slide 15-well 3D coverslips (ibidi). For FRAP experiments, the sample volumes were overlaid with 30 μl of ibidi anti-evaporation oil.

FRAP experiments were completed on droplets with diameter of ~1 μm, where half of the droplet was bleached in half FRAP experiments. Fifteen pre-bleach images were taken before bleaching (100% 488 nm intensity, 1 cycle for mEGFP, 100% 488 nm + 561 nm intensity, 5 cycles for Atto594). Bleach depth was consistently 60–75%. FRAP images were processed in Fiji. Briefly, fluorescence images were translationally stabilized using the Image Stabilizer plugin^64^ (4 pyramid levels, 0.99 template update coefficient) output of the brightfield images, and the mean grey value in the bleached, unbleached and background regions of interest was measured. Background values were subtracted from bleached and unbleached values before photobleach normalization using the unbleached references was completed. Full-scale normalization was completed using the average pre-bleach intensity. An exponential model was fitted to the post-bleach data (*y*(*t*) = A × (1−e^−kt^), where *A* is the plateau, *k* is fitted and *t* is post-bleach time).

### Native PAGE gel-shift assay

Rubisco and fragments were mixed in 5 μl reaction volumes and incubated at room temperature for 30 min in buffers as indicated in the relevant figure legends. Following incubation, 1.6 μl of loading buffer (80 mM Tris-HCl pH 8.0, 200 mM NaCl, 40% glycerol) was added before loading onto 4–20% Mini-PROTEAN TGX gels. Electrophoresis was completed for 4 h at 100 V at 4 °C.

### Surface plasmon resonance

All SPR experiments were completed in triplicate on a Biacore T100 system fitted with a T200 upgrade kit operated using BIACORE T200 control software with a sensor temperature of 25 °C. Immobilization of Rubisco was completed according to ref. ^32^ and experiments were completed with modifications. The analyte was injected at 15 μl min^−1^ for 30 s, followed by 360 s of dissociation. After each replicate set, the chip was washed with 1 M NaCl in running buffer, after which the chip was washed for 360 s with running buffer. Binding to the reference chip was negligible. Fitting of the reference-subtracted curves exported from BIAevaluation was completed using the Hill equation (*y*(*x*) = *B*_max_ × *x* / (*K*_D_ + *x*)), where *B*_max_ is the maximum specific binding, *K*_D_ is the apparent dissociation constant and *x* is the analyte concentration.

### Single-particle cryo-EM data collection and image processing

Chlorella Rubisco and the α3–α4 fragment were mixed in a buffer compatible with cryo-EM experiments (200 mM sorbitol, 50 mM HEPES, 50 mM KOAc, 2 mM Mg(OAc)_2_·4H_2_O and 1 mM CaCl_2_ at pH 6.8) at final concentrations of 0.5 μM and 16 μM, respectively, and incubated at room temperature for 10 min. α3–α4 had the same apparent *K*_0.5_ for CsRubisco in this buffer as the Tris buffer used for native PAGE experiments (Supplementary Fig. 10f). A volume of 2.5 μl of solution was applied to R1.2/1.3 Cu 400-mesh grids (Quantifoil) that had been glow-discharged for 60 s with a current of 15 mA in a PELCO easiGlow system. Using an FEI Mark IV Vitrobot system (ThermoFisher) with chamber at 4 °C and 95% relative humidity, the grids were blotted for 8 s with a blot force of −5 before rapid plunge-freezing in liquid ethane.

Data were collected on a 200 kV Glacios cryo-electron microscope equipped with a Falcon IV direct electron detector at the University of York. Automated data collection was performed using EPU in AFIS mode (ThermoFisher). A nominal magnification of ×240,000 and electron fluence of 50 e^−^ Å^−2^ with a calibrated pixel size of 0.574 Å was used during collection in which each exposure was 6.52 s. The 100 μm objective aperture was inserted and the C2 aperture was 50 μm. A range of defocus values were used (−1.8, −1.6, −1.4, −1.2, −1.0, −0.8 μm).

Relion3 was used for processing and 3D reconstruction^65,66^. Of the 1,568 EER frames, 32 were grouped to give a fluence per frame of 1.02 e^−^ Å^−2^. Relion’s implementation of MotionCor2 was used for motion correction before CTF estimation with CTFFIND4, assuming a spherical aberration of 2.7 mm^67^. Initially, 465 particles were manually picked and reference-free 2D classification was completed. A total of 237,035 particles were autopicked using selected 2D class averages. Particles were extracted with 2× binning and 2D classification was recompleted. 2D classes presenting clear Rubisco structures were selected and a C1 symmetry 3D classification was completed. A single 3D class with clear secondary structures was used for auto-refinement with D4 symmetry (73,962 particles). After CTF refinement and Bayesian polishing in Relion^68^, a 20 Å low-pass filtered mask of the 3D refined map, expanded by 10 pixels with a soft edge of 6 pixels, was used for solvent masking and resolution estimation. This map has D4 symmetry and represented the CsRubisco holoenzyme (EMD-18049).

In the 3D class used for the D4 map, an additional low-resolution density was observed on the equator of Rubisco, suggesting a substoichiometrically bound partner (Extended Data Fig. 4d). The predicted helix of α3 (Fig. 1f) was built into one region of the additional density in UCSF Chimera (Extended Data Fig. 5a)^69^. The soft, featureless mask was created from these coordinates by low-pass filtering to 20 Å, extension by 5 pixels and softening by 6 pixels. This mask was used for two rounds of C1 3D classification, with a D4 symmetry expanded particle dataset created from the 73,962 polished particles used for the D4 map (591,696 effective particles). A total of 133,171 symmetry expanded particles were used for the 3D reconstruction of the C1 CsRubisco–α3–α4 map, which was processed as for the D4 map (EMB-18050).

### Single-particle cryo-EM model building, fitting and refinement

A holoenzyme model of Chlorella Rubisco was built in UCSF Chimera using AlphaFold 2 structural prediction of the CsRbcL and CsRbcS sequences from *Chlorella sorokiniana* UTEX1230. This model was rigid-body fitted into the CsRubisco holoenzyme map using UCSF Chimera. Flexible fitting was performed in COOT (0.9)^70^, using one of each CsRbcL and CsRbcS chain. Real-space refinement was completed in Phenix^71^. The coordinates of this refinement were applied to the other 7 CsRbcL/CsRbcS chains. For α3–α4 model building, the AlphaFold 2 predicted helix of α3 was manually built into the additional density present in the C1 CsRubisco–α3–α4 map in COOT. The side chains of residue E169, R171, E172, E175 and R179 were removed before refinement in Phenix due to a lack of supporting density. Both the structures derived from the C1 and D4 maps were validated using MolProbity^72^. Figures were created in UCSF Chimera and UCSF ChimeraX^73^, and molecular contacts were assessed with PDBePISA^74^.

### NMR spectroscopy

Spectra were recorded at 10 °C on a Bruker Advance Neo 700 Mhz spectrometer equipped with a TCI Prodigy CryoProbe (Bruker). Samples were analysed in buffer containing 15 mM sodium phosphate pH 8.0, 150 mM NaCl and 5% D_2_O. Protein concentrations were 920 µM for α3 and 500 μM for α3–α4. Spectra were processed using TopSpin (Bruker) and analysed using CCPN Analysis^75^.

### *Chlamydomonas* transformation

Chloroplast transformations were completed by particle bombardment with a Biolistic PDS-1000/He particle delivery system (Bio-Rad). Per bombardment, 0.5 mg of 550 nm gold nanoparticles (Seashell Technologies) were incubated with 1 μg of plasmid DNA and prepared according to manufacturer instructions. Cells (1 × 10^7^) were plated in a 4-cm diameter on TAP plates and placed ~9 cm below a 1,100 psi rupture disk. After firing under vacuum conditions, cells were recovered for ~24 h before re-plating to selection conditions. Transformants into the ΔEPYC1 strain for RbcL knockout and CsLinker reintroduction were re-plated to TAP plates containing 100 μg ml^−1^ spectinomycin under low light (~10 μmol photons m^−2^ s^−1^). Transformants from the ΔEPYC1ΔRbcL complementation with D86H mutated CrRbcL were plated on TP plates and recovered in 3% CO_2_–air mix at ~50 μmol photons m^−2^ s^−1^. Sixteen transformants from each transformation were propagated 4 times on selection plates before checking for homoplasmic integration of the genetic material.

Transformation of mCherry-tagged *Chlamydomonas* RbcS was completed with pLM035 (ref. ^15^), using a NEPA21 electroporator according to ref. ^76^.

### *Chlamydomonas* growth assays

Spot test assays were completed according to ref. ^15^.

### *Chlamydomonas* confocal microscopy

All images were captured on a Zeiss LSM880 confocal microscope in Airyscan mode with a ×63, 1.4 numerical aperture (NA) Plan-Apo oil-immersion lens (Carl Zeiss). µ-Slide 18-well chambered coverslips (ibidi) with 10 μl of cell suspension and 30 μl of 1% TP-low-melting-point agarose were used for imaging.

### Transient expression in planta

Overnight cultures of electrocompetent GV3101 *Agrobacterium* harbouring the relevant plasmids were grown overnight in LB. Cultures were resuspended in 10 mM MgCl_2_ to OD_600_ of 0.8 and syringe infiltrated into the youngest fully expanded leaves of 4-week-old *N. benthamiana* plants. Images were captured using a Leica SP8 confocal microscope 48 h after infiltration and incubation of plants at 25 °C, 16 h light 8 h dark, and 170 μM photons m^−2^ s^−1^.

### Reporting summary

Further information on research design is available in the Nature Portfolio Reporting Summary linked to this article.

## Supplementary information

Supplementary Information Supplementary Figs. 1–16 and Tables 3–15.

Reporting Summary

Supplementary Tables 1, 2, 16 and 17 Supplementary Table 1. CsLinker and RbcL co-immunoprecipitation results. Table 2. CO_2_ response differential gene expression analysis. Table 16. Oligonucleotides sequences. Table 17. Reagent catalogue numbers and availability.

Supplementary Table 10 Supplementary Table 10. Fluorescence recovery after photobleaching (FRAP) analysis.

## Source data

Source Data Fig. 2 Unprocessed SDS-PAGE gel for Fig. 2c.

Source Data Fig. 3 Unprocessed native PAGE gel for Fig. 3a,g.

## Data Availability

Proteomics data were deposited in MassIVE, with ProteomeXchange identifier PXD044179. Electron density maps were deposited in EMDB with accession codes EMD-18049 (D4) and EMD-18050 (C1), and their corresponding coordinates in the PDB with accession codes 8Q04 and 8Q05, respectively. Differential gene expression analysis was completed using publicly available dataset PRJNA343632. All other associated source data are available in Zenodo^77^. Source data are provided with this paper.

## Code availability

Code for the Fast Linker Identification Pipeline for Pyrenoids (FLIPPer) is available in GitHub^50^.
