# Supplementary material for: A promiscuous mechanism to phase separate eukaryotic carbon fixation in the green lineage
Source: Nat Plants. 2024 Oct 9;10(11):1801–13. doi: 10.1038/s41477-024-01812-x (PMC11570498; doi:10.1038/s41477-024-01812-x)
Supplement: Supplementary file 1 — Supplementary Figs. 1–16 and Tables 3–15. [file 41477_2024_1812_MOESM1_ESM.pdf]

nature plants

Supplementary information https://doi.org/10.1038/s41477-024-01812-x

A promiscuous mechanism to phase separate eukaryotic carbon fixation in the green lineage

In the format provided by the authors and unedited

Supplementary Information

A promiscuous mechanism to phase separate eukaryotic carbon fixation in the green lineage

James Barrett[1,2], Mihris I.S. Naduthodi[1,2], Yuwei Mao[3,4], Clément Dégut[1], Sabina Musiał[1,2], Aidan Salter[1,2], Mark C. Leake[1,5], Michael J. Plevin[1,6], Alistair J. McCormick[3,4], James N. Blaza[6,7], Luke C.M. Mackinder[1,2]*

[1]Department of Biology, University of York; York, YO10 5DD, UK.

[2]Centre for Novel Agricultural Products (CNAP), Department of Biology, University of York; York, YO10 5DD, UK.

[3]Institute of Molecular Plant Sciences, School of Biological Sciences, University of Edinburgh; Edinburgh, EH9 3BF, UK.

[4]Centre for Engineering Biology, University of Edinburgh; Edinburgh, EH9 3BF, UK.

[5]School of Physics, Engineering and Technology, University of York; York, YO10 5DD, UK.

[6]York Structural Biology Laboratory, The University of York; York, YO10 5DD, UK.

[7]Department of Chemistry, York Structural Biology Laboratory; York, YO10 5DD, UK.

*Corresponding author. Email: luke.mackinder@york.ac.uk

Supplementary Figures

CSI2_123000008654

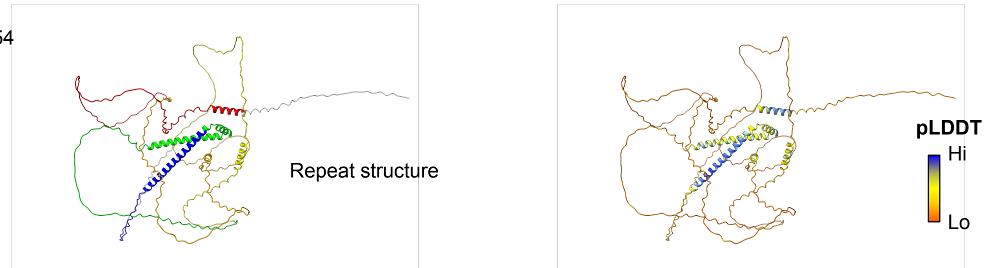

MSQCTIQHTEADAGLLSLAAPTSDAEGWLVPVAQPASRQERLSEQASLFMANLLAQAAPGTSNWLSAEPADPFAPFQQQTAAPPATSAQQRRQLTSEGSEVQPLPCVSLAGQPPRSQRRPSSVTAPQ
PRPGSALPRPASSFPRPTPKPAASASRPAMPAQAAASQAASPKPLWRPSGSAQPSPTTPRHSTPGTPTRQSLAGTPPAVRTAAAPRGLASTSQQAQQAQRPASAAAKQAPRAEELSPSTKVAQWLFS
PAHPAAPAGRLTVKRPASSAAAAAAAAASLASPSASSSEASWRIAAATGTGSTLSSPAGTPAAAAAAAAAASGFRRQASGSRIPRPGTAASAAAPAEPLPAEGTSPSARAAAPASLPTRRSELALKQ
LAQRALAAGSTSPPAPCPLHVRRPSSLRGAAQAASVAAAPASTSLPSSQPTSPFQQTFVSARSADAASVSSYLSAAESPAGETTPRRAHRLSSSSSSQPASSQPGSPAALPLSPRPGSAGSQLRQAQ
AAAVGSLAAAQAGARAAAEEVALLSTASPGAWAAAQAVAAVSPASDTSLLDAQLALLDRLSRDLSDASSQIAEVCILDTLANSRRASSSMPTARRLSLQLHSPGASSAGASSAGAAGSPPRPRSLAG
SLHGQHHAM

CSI2_123000010295

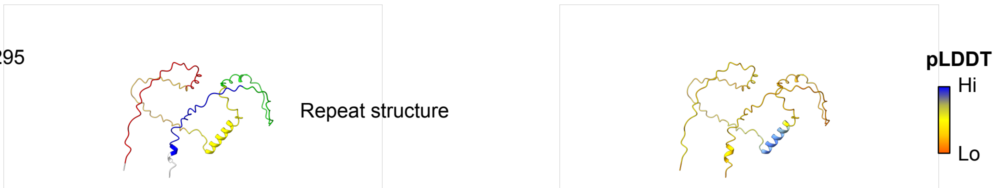

MARPRGKGSKPSTTQTAAAPSSRKPKGGRPRPASKAASGGGKPGGRPSGSSKPAQLAGAAHRIVEFAPPPGPATLAERFALLEKQRQQLGAGRQRAAAPALGVASGGVAKKAKQQRRRKGKASSGE
AGGAGASGKPAAAKAQGAAAAGASSGSGKRRRRGGKRSQAAKMDTK

CSI2_123000010558

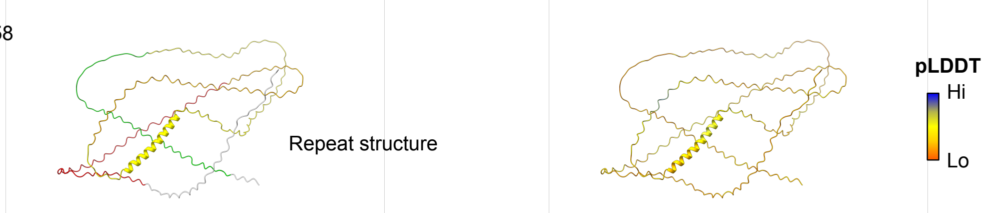

MREVAALANTSSPAREWAAAKTASPSSAAAGPPAAKRRLQDDAPQDFVIAAISGSAAQAVAPIEPAAPSQAAADAPVPPAAATPAAPAPTIPAPAAEEQSPPAVVEALFSDAASPAGTAAAAEAPGA
PPAQPARAASLNRSPAVSHAVRSLGPVAESSPAVPLPPLPTLAPVxxVAGSATSPAPARSISLTRQLSRKLSRGMSRLRASIRRRLRRADSPPVPRSRDLSGRISPTSGTAHLPAIPEGAPASAAAA
AAGSGGLGGQVELATLAPPHPAATAAVERAASVAAAPSPAAAAAPGAETAAAAGSQASRASLVEVDLSEQRPAPSSSPSRRRGLACACFA

CSI2_123000012064 (**CsLinker**)

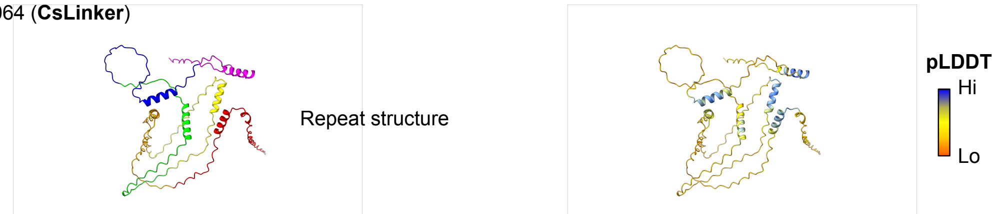

MQALSARVTLGRPATARVAKKQQRPLTVAMATAQLSAEQKAFLERKARESRSPAPVETRGRSAPRPRSASPVGRPSSRTPAPASYSAPAASYSSAPANGLSAEQREFMERKARESRSPAPVETRGRS
APRPRSASPRPTSRTPAPASYSAPAASYTAPAAAPAGGLSAEQREFLERKARESRSPAPVETRGRSAPRPRSASPRPTSRTPAPASYSAPAASYSAPAAAPAGGLSAEQREFMERKARESRSFSAPV
ETRGRSASARPSARPSSRTPTPVSNSGVRSAMSSGSSAGLSAEQREFLERKARESRSPAPAAASQSSSYGSAASGLSAEQREFLERKARESRSFSAPAPTRGRSSTPRRR

CSI2_123000002244

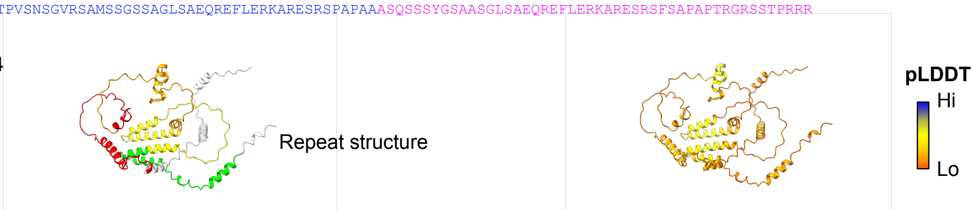

MASALRPSAACIARRQASDWNKTRVVPKSAFATALHAKASNARALPHSASSRIEAVHVEAALVELAAPAAFPPLPGTTTTRPATLAGAWAARQQAAASCAAVPAARGVSEDAEDVAPRLPAAYLPPA
RREGATATILAGCSLAYPAPAQPAQRPAAYVPPHSRTAEQQAAAAEACRPGRICAWPYLTAGPLPQPAAEAPLPLYRLALLDIDDSTEGNARMRLAGLLMLAFATMMHFLGSPQAPFLYLFAWMAGV
LGPSRAGTSEPHAPQPPPPLPPAPSDIADSSEGNAHMRLAGLLMLAFAMMMHFLGSPQAPFLYLFAWITHPQDEHVAHRCCAPEPERAAQRRHARAARPTAAAATAAGAQ

CSI2_123000002308

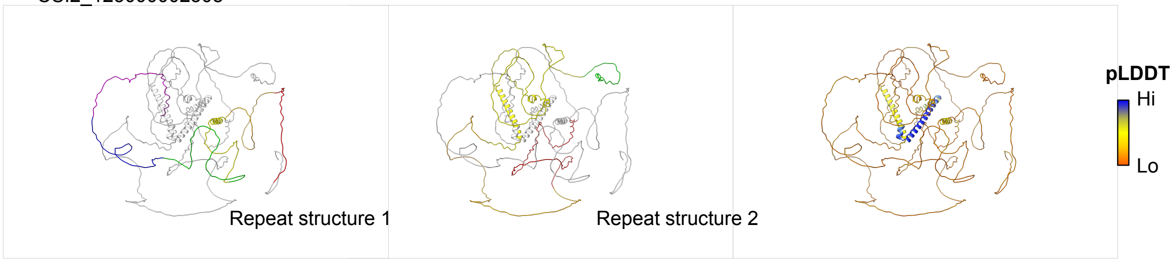

MQRGAPPRDCPEQQGGVAALAASPGARPAGPGAGTPCCPPSSPLQPIAEAGPAPAAPGCPAPALDAGAGVMRPPPPKCAPLSGSLATSSQREALTAQHSKLLSSEAATEERDEYLMRLLCKGAPPAG
VPPAPPAAQVPQGAPPPALQQQAVQAQAAQAVEAPRFRAPGPVSRSVPPSAPAPGSATPPAPSPSGLLPRATSTTLASPSSTVGAVTGTACATVPSLPLPAPSATATTTDQAVPSSGGAAAAPTTAA
PPPRAAPPLPPSARAAAAGAAAAVTALPAPQLVAVPLVAVPPELAATFDQTRRMLGDEIALSVRDTMIRQQVMFLEQLYDLHRAIAIQKLLMQNCPEVMGEAMRLMSSVGSQHGSGSGRGSKRQRTT
PNGGATVPGSGSGDPSSERPDSQFAAVPQLPGEAQTRDDEDDDGGSGDANGSGSAGANGSGGGSTSPQQLVPSLPPPPPLPYMYLAHTAVATPSGASAAPVGWQWGMPPLVQGQVQQLQQAVGLPPV
PRGPPQMAPWVGQDAGLAAVGINTHTPQHPKHAAGAAGSKPSKTATAATAPPAPRATPPRPAAAQPSAAAAAAPAAPAPQPKQQQPVAQPQPQQAPLALAQAPAAPAMAQPAPASLAMPPPGMPPPM
PFGPPPPHxxxAxHGPPGLPCPAPGAADPMAWWYQNYYGSQQQMGGMSLATAQAAAAAAAAFMQQAAQQPGGTGCTTAGPLGTAGTAGGTGTGGPAVTKWWHDPQLTFGPPVDTEMLARRPAGGPAA
LLASAREGPEVQSAPAGQLKRPADSRRRSSMPYSDGPRMRLKRKATADADEASSGTTGHTHDPDGGAGTTAGGPFKKHESLRARSARTSPQGPAAADDPMGGGGQDENAAQLLLAIGKQ

CSI2_123000011700

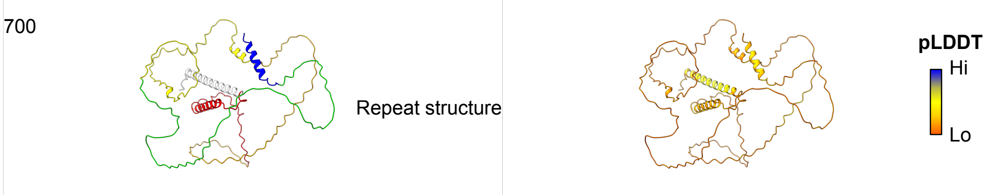

MAAPVTSSDATRNITLACIVAGTVLLVVFVAWKMRRRVRCSCSCWSLADPAAAERRRVLASAAVLYQAAAANPDFKLRLQPGPGRWPQPSPAASVYTSPTLAPPDPGSTLGTPASWRSRSPTGSYQA
ELAPVPAAPPLRSRTSPSGRPPLPPAPPRGPAQPMASPGTSPASSQQQRRSPPQRRSVRELVRAWSGGGSQNGRERSPPAVTAAAAAVAAADLEGQQLSATHEAQAFTWSQLGLSPAASPAASRRVS
ENAAAPYRLPLPPSQQGEVHSNPVFDWTAAGVAATDGGTLGPAAGSFSQQVKRTASRSRLSRGVSFAEPAGEGSAHGGREGGGHSRRGSFVTPEQGSRREAGGQMSSRPSEQALLQLHCELEGIQE

CSI2_123000004449

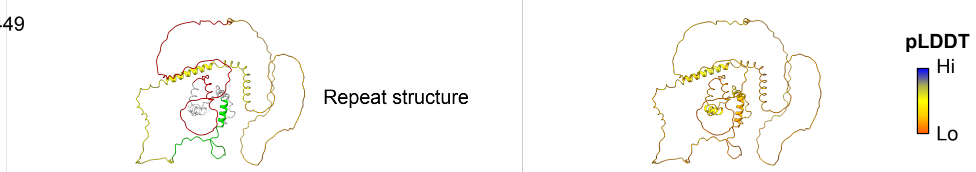

MASGRLALPPRQGGSAEQRKRVSDEAIADAVEKLSGAPVGAVERLAGAPPALTDLAAAAEAAAAGGSTGKKRKAEEASPPAAAAADGAATGERQTTGGRSRRSNAGKKPLRAEFDDDGDSWDDESEE
DSPPRGKRRGKAKADSDFEDFSEPESASESEEDSEDEFASEDEDSPPKCGKKAPARGKGAAGKSGAAAGKKSGAAAKKSGAAAAKKSAAAAKPPAKAPAAKKSAAAAKAAAPPKPAAKSGVGKKAAG
AAAAAAAAAAAAAEEAEQVPEEQQQLEQELPVQDTAQLAAAAAQQAEEEAAAEEEASAAVPPPAKKAKVPAPKPAAAPKPAAAPKPAAAPKPAAAAGGSKPVGLKAAGGGVRKPLAPAAQAKAPSPA
GGGPSPAGGGGLMAAAARVVQGAA

CSI2_123000004744

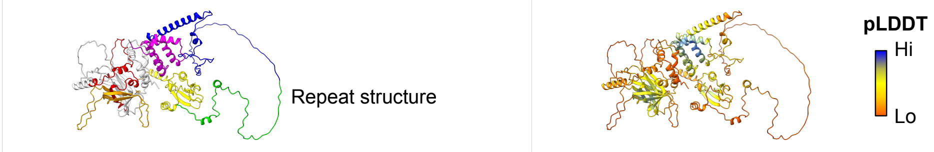

MVQPAAAAEALALQLQATELENILDARDKGRTPTILRRNLSYVDRSRRRALQQGHGAALQLQLELAGGPGPAGGPMQTSSAAAGSSSPDRATRHVLVAAVCIVAPQNGTQAYVPVSARTVRLPPERH
ARLSLCLPLGAASPAAGLAVVLLHPFAGDARWRYRYRLSNEEQRRWNAAQAAAAEGQHMQPPECGQLALLASLAPALHRRSRSSAASSGSGGGSGDEGRLLEPSITAAAAFTLLPSGSQVGYSVISL
DGLLGGRPKAEGFIQRQHGVLTTHVPQLGASAGGAERGLASPWFQLDSRGLGLHQAAAGRSSSGSGAASGGDSHPPFRYTATLVSGSGGATQQRGGQAPPAVQQQQQQQQQQQQPEPQITYFFCSFD
GSCSVRSVANGFRCPLASCRGLRCHSYAALQQHIGAMHGYHASYFSGLLPDGGLEVYLRCKPAWLTPKLEFLPRQLGRVEEHPLLDLLSMSHNRYPFYFHCPRWQRRRRLGPWGAAPRDAREEELER
QAEANIAVRLGYAPPQPSSSAPAASSMYSVETLSDSGTGTELTAAEEAGPTAASGQRAAKPAGGRGRGRAGGGASGRGRGRAAAAVAGHHPASGLALLNGEGRPKFYHSRTCVAMTKEELFAEAGQA
GGDASDSEGDLSEWQASWWCLFVCRDCRERLAEHKGLCPEERAFMFEWNLFLHRRPLHADGDMPAALAAFAAGHREELGRDGPFRRCMVAHLLNLWRFRLLTPSQLHQLTRELPRVAPL

CSI2_123000000046

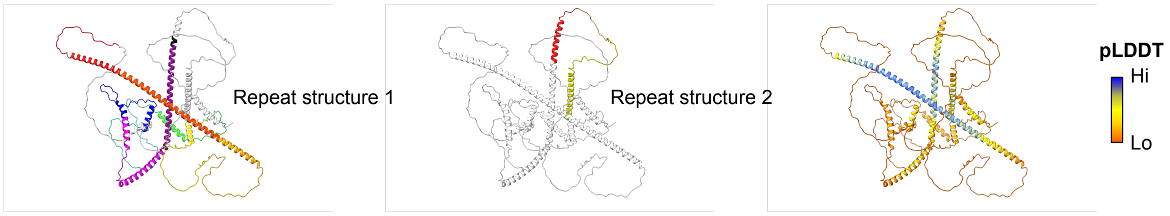

MPGTGVWDSCQQFLEAVERQQQRASSPCPQPSCPQQPPLSPQHPPQQQPQQQLGEQRPPSRTSSQPQQWPLPMASPFAAPNVQQAAPEPSGSAGAAGGEAKMVSPFGAAYLQEQAFSTGSLPAPSAP
LPDAKTLPSAEGPLHQPTRSGGESPPPLAQLQPQPAQQQGGAAAGQQQQRRSPSPDQWAAQQAQLEQQEAQEQEQQLEQHRAQAHQAALLAQQQRQKQAVAMGRLIEQQQQQQSALLEKQLAAQQA
ALEQQRSEQQQWCTAAMRLIEQQQGQQAQQGQQGQRAQQMDWAAQRTQQAPPPPACPGCRQPVLPAVACACDGPLSLRRAGTANRSVLMVEASSLSRAGSSPGPSRAPNHISQASIAGAKVEADEDQ
QMQEVLSIARELGVFPTCPADLEAQPSWVPPSLGAPPPPPPLPRADQRRSEESAQSQLQLLQQQLLQPRSHAPGLTRGHSHPPPASTQQQQQQLLQPAPQLRREEWSAGGGAAPPALLAQPGMQQQP
AGRQLSADQLFELAHLLQRAKAAGIQLPQLASWPTQAPGPQAQPPVQPQLPPQQLHAAAQQQQLHGLVHQQQQQLAQPQPQPRRAETATFEHRSAMLAGLRRQISADAIHAHHVQQAQHAHRVQQAP
QVQQAQQAAMHRQQLMLLEAAQAEQRKQLVQAQHAELLQLEQARQARQARQAQQAQQAQHTWLAADARLAAPPAPISQHTLQFAGPQQQRSAYAAPPAPLQRLPTAQQQLQPPPLQQGAASLYAQQQ
ALLAQQRAEQQRWADAALSLLSQATR

CSI2_123000000713

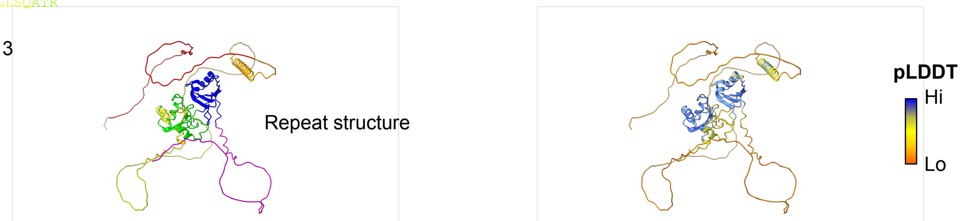

MSLFAGLFAAPKQADSPGGATLFSSSSKYAAQPLAAPAAAAAAAPEPGGGKKKKRKQEAAEAPAAPAAAAAEPSSKKARKAPQQRRQQGQEERQQQADKAAAAAAAAAAPAEGEKKANKKRKQQEGA
QQEQQGAAAPAAAAPAAAADGRGAKASSKPGKRDQASKPKPAAPAAEAAAEEGRSKAPGKAGGKATEPAAASKGKGGKAEAPADERLPRTVFVGNLPATVKTKKIARLFAAAGCGAVESVRLRSLPL
KQEPESKLPRKNAIAAGKIDSTHSAHAYVVFEEEAGVDKALALNMTEFEGHHIRVDRAAPASAKGAVAFDHMRTLFVGNLPHDCEDEELIRHFGAQAVEAVRIVRDGKTGAGKGVAFVLFRSQEASK
AALRARGSGELRGRKLRLTVAKKQAAAGGKPAAWQQGKDSGGKGGGKSGGKSGKPAAAQRKAGGKRPAVAARKAAAKMAAKGGVQKKRAA

CSI2_123000000775

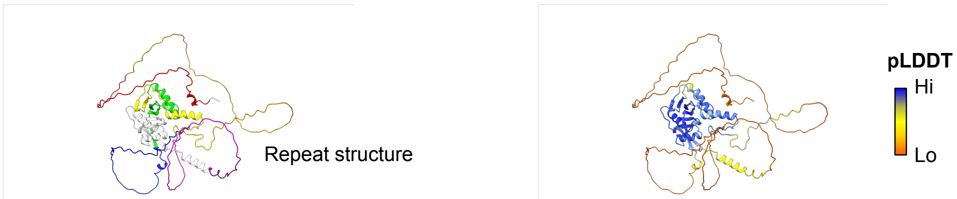

MQPPPGSIRAFFKPASKLAGAKRASGGSAAAAPAAEAPSDAIAAASEPAAAAADQLPPPPAVFARKRRRPSGGGSAGAAAEPTPAAAAAAAAGGDGAPAAAAGPSSAPATAAAAVEGGAAAGSRTRQ
LTQMYLDVGQRHFHAYRCPACGMLYARGTDADERLHAAFHASATQGPRYQGWAGERVVQLDGSKGRVLLFGSADQGRPRKQLADLCSFLEEQMGLSKGWLLQAPLTVLLYVSAARRVQGFVAAEPIK
EAYCARPSLPPAAAQGMLSCGSAVQPASGSGAAHAADAAATVAPFEGHPTAAAAAAQAHVATSGRSLLSVTLSTGAAAAECSSSAAAEGAGGGAPAAAAPAAAEAGDGEVAAAAGCTPGLPTSLQQQ
QQAQPEAQQAQQQLQQTQQPPVLPPLELDRSRRVRAVCGVRLMWVSLEARRRGLASRLLDCCRSHFMPGYVLSRHELAFSAPTDDGRAFIEQYAGSRKFLVYE

CSI2_123000001971

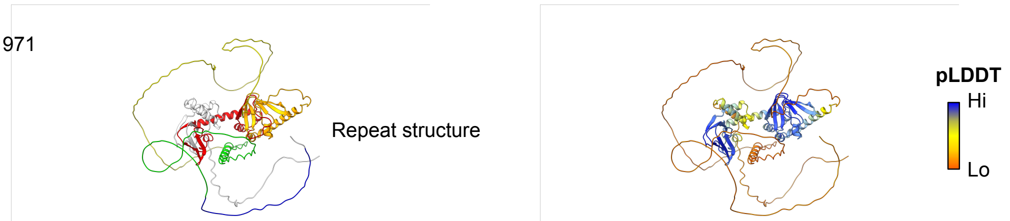

MGCGFSKGGGLRRGAVAPAPEEPAQAVGAQAGPPGGPTKRRGSVSAEPSGVSRSLSQAAGRQSVANQKALGTRERIRAIVKSTLLFKGLDEEQQGRMIDAMEERKVAAGEEIIKQGEEGDNFYVVES
GRFRATKSGAEVAAYDGAGSFGELALMYNCPRAATVTALSDGVLWALDRATFRALVVGSMAERAQRYEASLRRIPILQHLSQEQLAAIADCLEQERFEPGEYIVWEGAPLDDGAKFYMIESGRVDCF
RTFEGQKRAVKGLRAGDVFGEVALLTKAPRQADCVAATPCKCLTLSRAAFERLMGPVEQVLAQQIAQYQASNAQVTAGIDTTTASSSLPSLREDAAAAGNRGSEGAADDSKGDAGGSSQPPTSAGAG
KPPARRGSSPRSPAAGGGGSPAAGGSAAALLPGSPRPSMLSEGSAGSGSFSRKRSSPRATSNSLGARSARSTTNSDSAPAGEGDCPLGAAATGSGSGSGSGSGGAAELAVAMEEAAALSEQEPVCHL
PPIAEEPAEGATPRTSNASTQGSLQGQPLASSKERRSTAGGSTKGAAGGLSSGRRTSSGSGGKGKGPGSRPGAVKPAGSKPKAGSSKKK

Supplementary Figure 1 | *In silico* characterization of FLIPPer candidates. Top ranked AlphaFold 2 structural predictions of the 13 candidate sequences identified by FLIPPer, and their accompanying full-length protein sequences below. Models on the left are colored according to the repeat sequences identified by XSTREAM, consistent with the coloring in the protein sequence. Gray sequence was not represented in the repeats. Models on the right are colored according to pLDDT score.

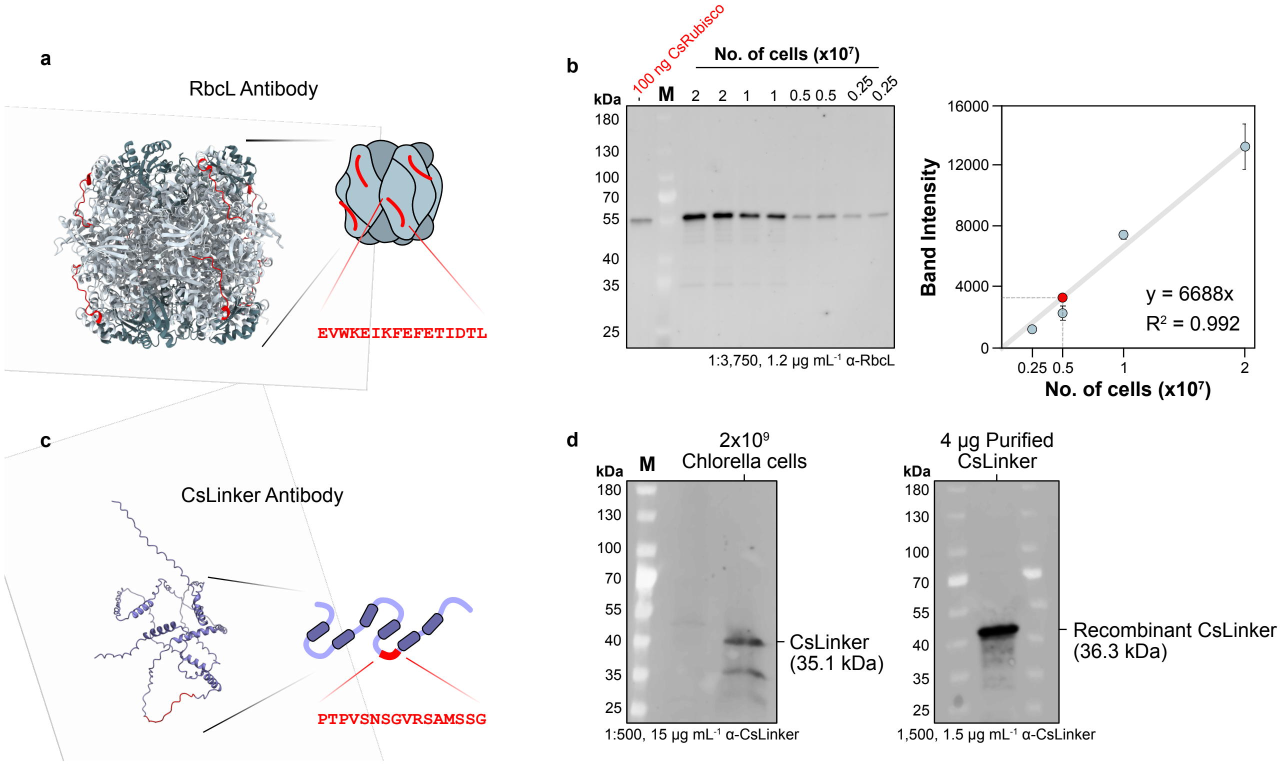

Supplementary Figure 2 | Antibodies raised to Rubisco large subunit (RbcL) and CsLinker. a, Structure of *Chlamydomonas* Rubisco (PDB: 1EJ7[40]) with the region of the RbcL that the antibody was raised to colored red (left). Schematic representation of the location and sequence the peptide was raised to (right). **b**, Quantification of CsRubisco by western blot from lysate compared to purified CsRubisco. Band intensity was quantified and used to determine the amount of CsRubisco per cell. Error bars represent standard deviation of the mean of the two biological replicates in panel b. The results of this experiment were from a single, non-repeated observation of two biological replicate samples. **c**, AlphaFold 2 structural prediction of CsLinker with the antibody peptide colored red and shown schematically adjacent. **d**, Western blot validation of the CsLinker antibody against lysate (left) and recombinantly produced CsLinker (right). Recombinant CsLinker has a higher molecular weight due to the presence of scar amino acids introduced by TEV cleavage sites. The observation of CsLinker molecular weight was also observed in a separate experiment (see Extended Data Fig. 1b). Blotting of the purified CsLinker was from a single, non-repeated experiment.

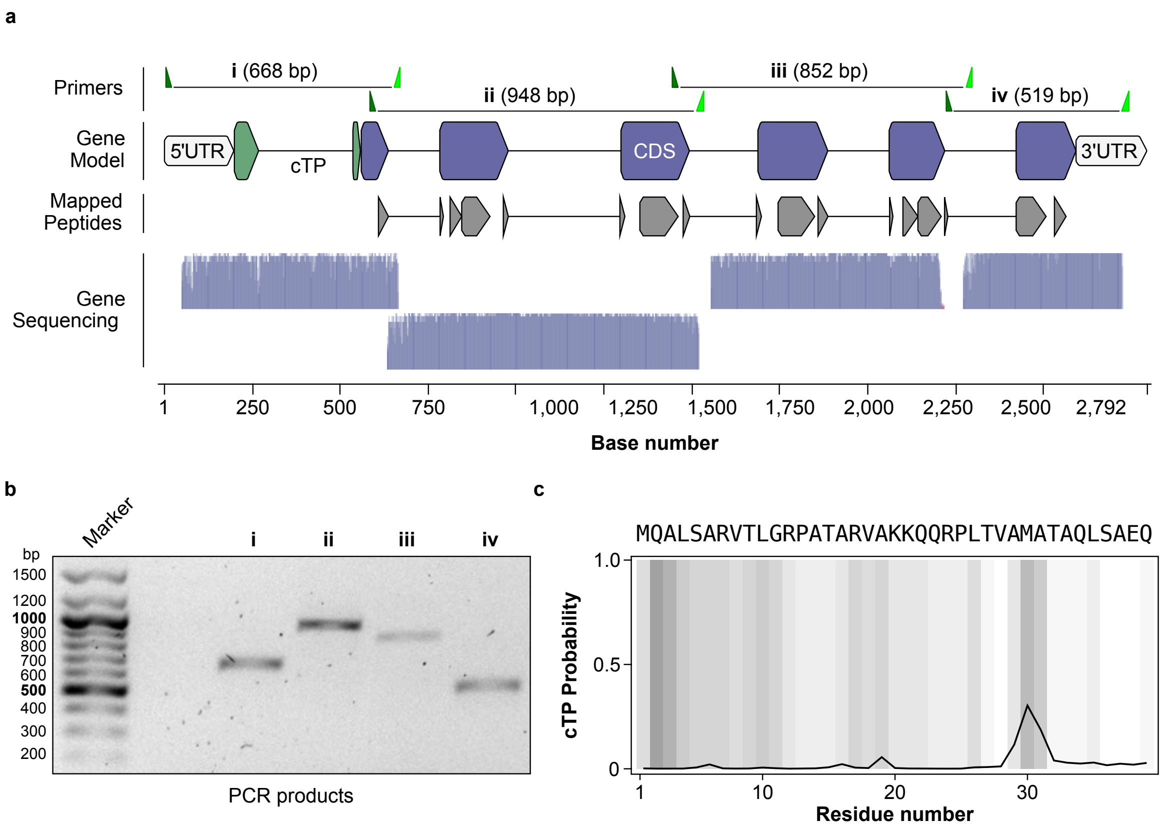

Supplementary Figure 3 | Validation of CsLinker annotation. a, Schematic summary of the predicted gene model of CSI2_123000012064-RA, CsLinker. The primers used to amplify the sequences in b and their expected lengths (top) are shown relative to the gene model (below). In the gene model, exons are represented by blocks, connected by lines representing introns. The predicted chloroplast transit peptide (cTP) from c is shown in green. A selection of the mapped peptides from mass spectrometry experiments are shown below the gene model where the mapped peptide sequence is represented by blocks aligned to the relative position of the corresponding amino acids in the gene sequence. Peptides that span introns are represented with a line between adjacent blocks. Sequencing results, represented as quality chromatograms, mapped to the gene are shown at the bottom of the figure. **b**, DNA gel electrophoresis of PCR products corresponding to the mapped products in a. The same products were sequenced. The PCR and sequencing results were from a single, non-repeated experiment. **c**, TargetP 2.0 prediction of the chloroplast transit peptide location in the first 39 residues. The predicted cleavage site is after the Met30 residue.

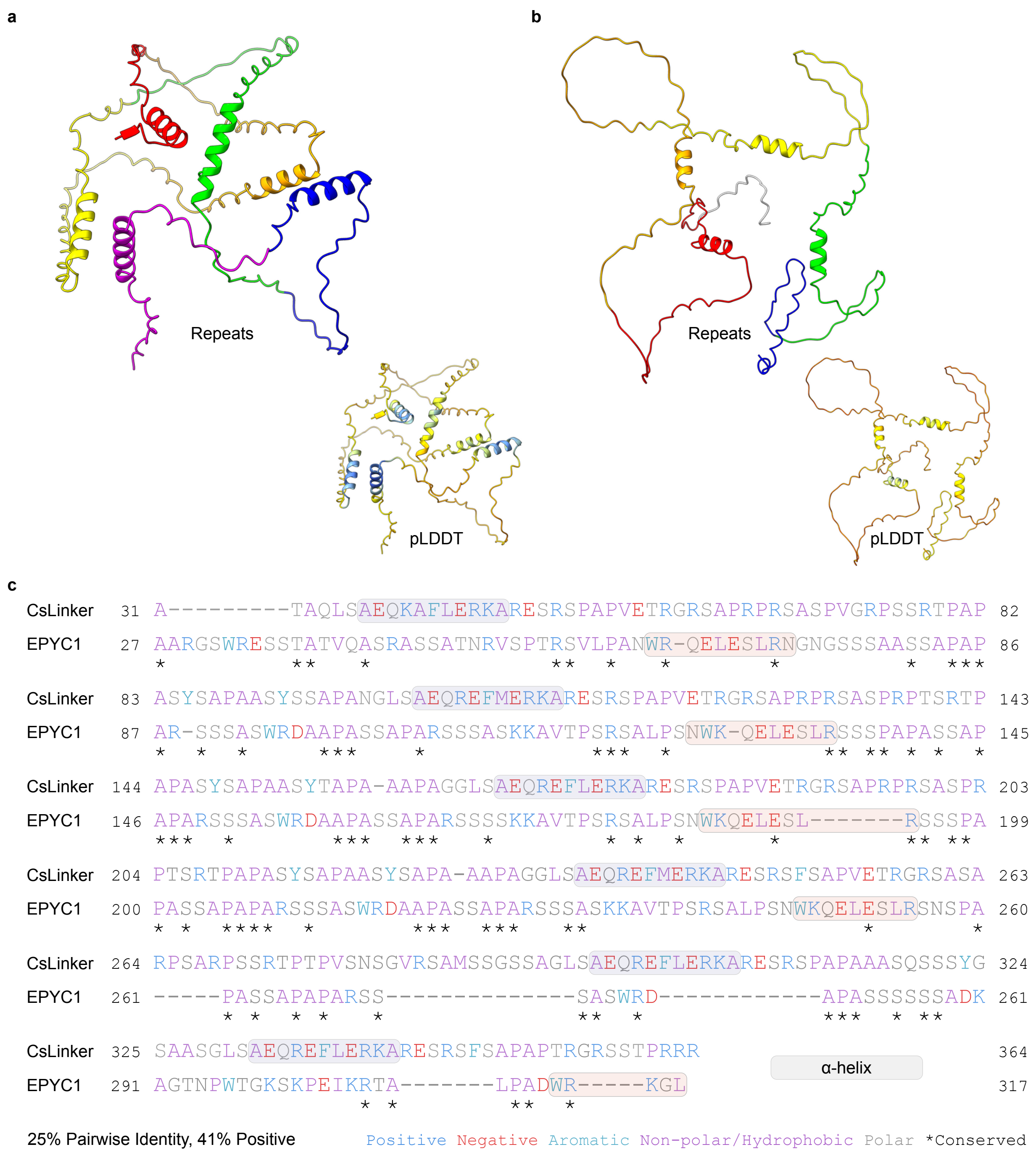

Supplementary Figure 4 | Comparison of CsLinker and EPYC1. a, Top ranked AlphaFold 2 structural prediction of CsLinker displayed without the predicted chloroplast transit peptide (cTP) - colored by XSTREAM-identified repeat structure (top) and pLDDT (bottom). **b,** Top ranked AlphaFold 2 structural prediction of EPYC1 without cTP. **c,** Alignment of CsLinker and EPYC1 sequences after removal of the cTPs. Residues are colored by property; conserved residues are indicated (asterisks) and the predicted α-helices are shown. Alignment was completed with MAFFT v7.49[75] using a BLOSUM62 scoring matrix with a gap open penalty of 1.53 and an offset value of 0.123.

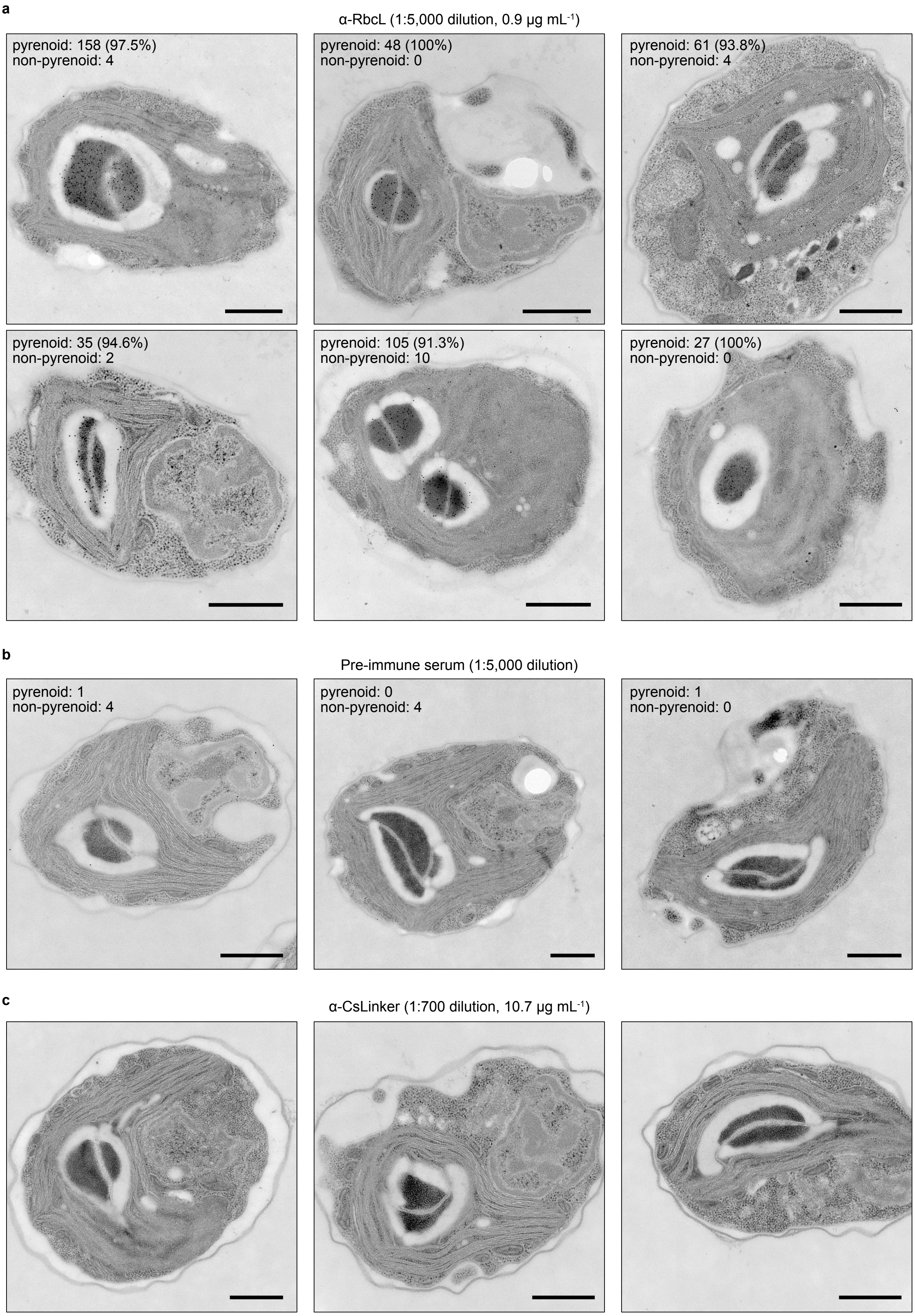
a
α-RbcL (1:5,000 dilution, 0.9 μg mL-1)
pyrenoid: 158 (97.5%)
non-pyrenoid: 4
pyrenoid: 48 (100%)
non-pyrenoid: 0
pyrenoid: 61 (93.8%)
non-pyrenoid: 4
pyrenoid: 35 (94.6%)
non-pyrenoid: 2
pyrenoid: 105 (91.3%)
non-pyrenoid: 10
pyrenoid: 27 (100%)
non-pyrenoid: 0
b
Pre-immune serum (1:5,000 dilution)
pyrenoid: 1
non-pyrenoid: 4
pyrenoid: 0
non-pyrenoid: 4
pyrenoid: 1
non-pyrenoid: 0
c
α-CsLinker (1:700 dilution, 10.7 μg mL-1)

Supplementary Figure 5 | Immunogold localization of Rubisco in the pyrenoid. a, Transmission electron micrographs of Chlorella cells that were immunogold labelled following primary incubation with RbcL antibody at 0.09 μg mL^{-1}. **b**, TEM images following incubation with a 1:5,000 dilution of the pre-immune serum from the same rabbit used to raise the RbcL antibody. **c**, TEM images following incubation with the CsLinker antibody at 1.07 μg mL^{-1} (1:700 dilution). Scale bar in all panels = 500 nm. All results were from a single non-repeated experiment of a single biological replicate in each case.

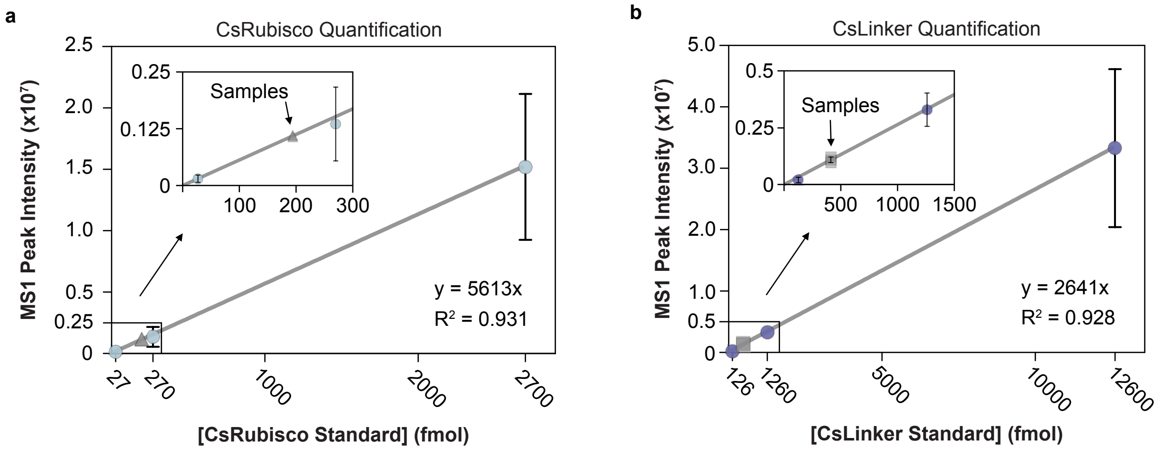

Supplementary Fig. 6 | Absolute quantification of CsRubisco and CsLinker. a, Standard curve obtained using purified CsRubisco (blue circles), used to quantify CsRubisco abundance *in vivo* (grey triangles). **b**, Standard curve used to quantify CsLinker abundance *in vivo* (grey squares). In both panels, for the purified protein standards error bars represent S.D. of the mean of two technical replicate injections. The zoomed inset shows the position of the *in vivo* samples in which the error bars represent S.D. of the mean of three biological replicates for which each point was derived from the mean of two technical replicate injections (see methods).

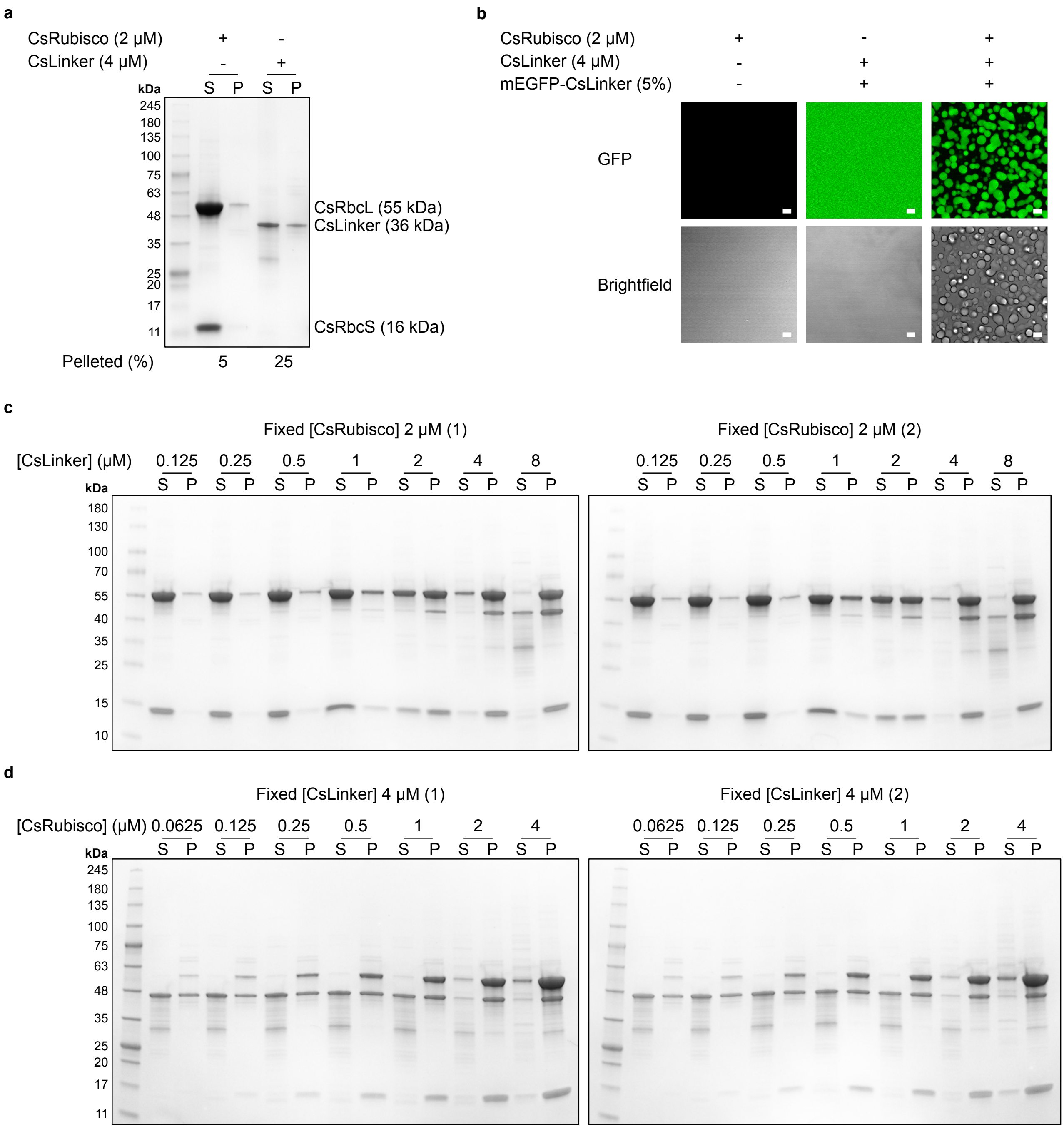

Supplementary Figure 7 | Characterization of Chlorella pyrenoid reconstitution *in vivo*. a, Control droplet sedimentation assays in which CsRubisco and CsLinker were incubated alone and analyzed by SDS-PAGE. **b**, Confocal fluorescence microscopy images of control droplet assays. Scale bar = 5 µm. Results across panels a and b were from single, non-repeated experiments, excluding droplet formation in panel b, which was observed multiple times across separate experiments (see Fig. 2, Fig. 4, Fig. 5 and Extended Data Fig. 8). **c**, SDS-PAGE analysis of droplet sedimentation assays with CsRubisco fixed at 2 µM and CsLinker titrated as indicated. **d**, Droplet sedimentation assays with CsLinker fixed at 4 µM and CsLinker titrated. All experiments were performed in a 50 mM Tris-HCl pH 8.0, 50 mM NaCl buffer. Where relevant, Rubisco was always added prior to the addition of CsLinker, followed by aspiration of the solution. The results in panels c and d are from 2 separate experiments that were completed concurrently.

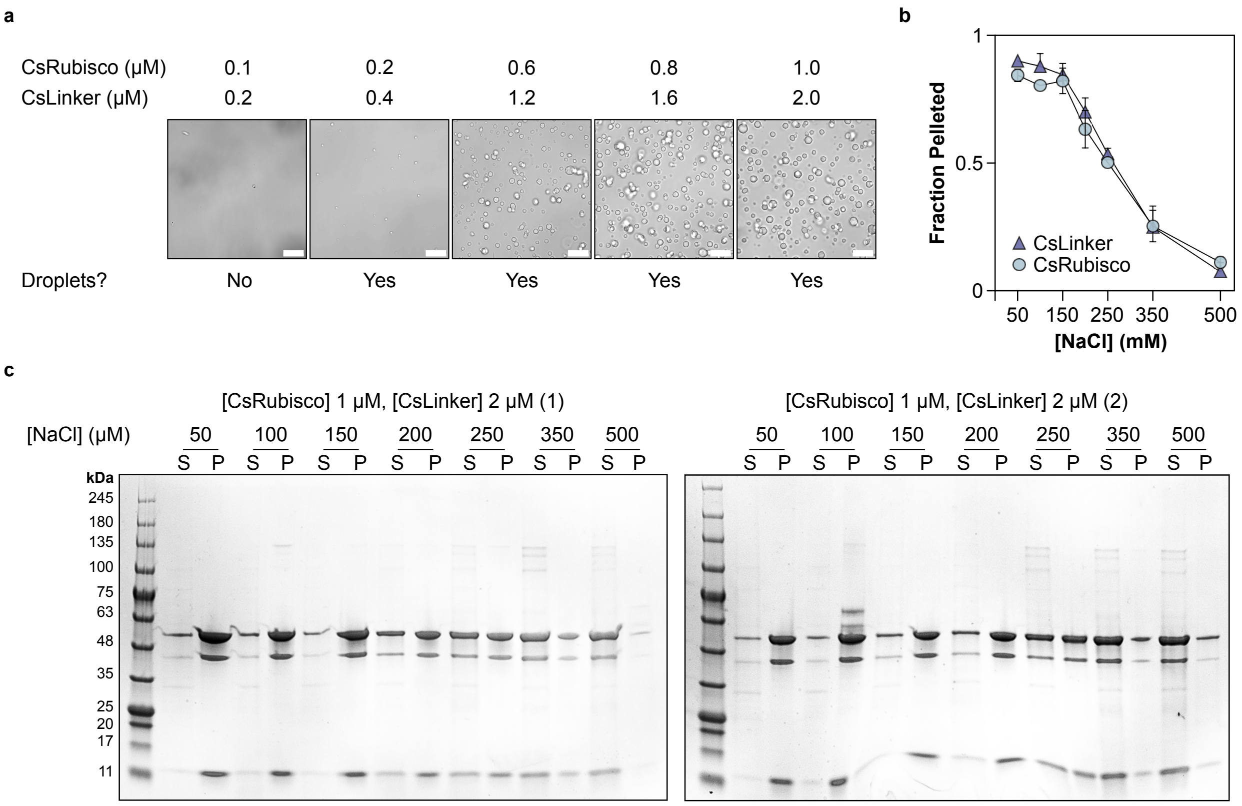

Supplementary Figure 8 | Indicators of droplet formation by LLPS. a, Brightfield images of droplet assays performed at increasing global concentrations of CsLinker and CsRubisco to determine a critical concentration for LLPS. Scale bar = 5 μm. **b**, Quantification of CsLinker and CsRubisco pelleting in salt-dependency droplet sedimentation assays. Error bars represent S.D. of the two technical replicates completed concurrently in panel c. **c**, SDS-PAGE analysis of salt-dependency droplet sedimentation assays. The results in panel c are from two separate experiments completed concurrently.

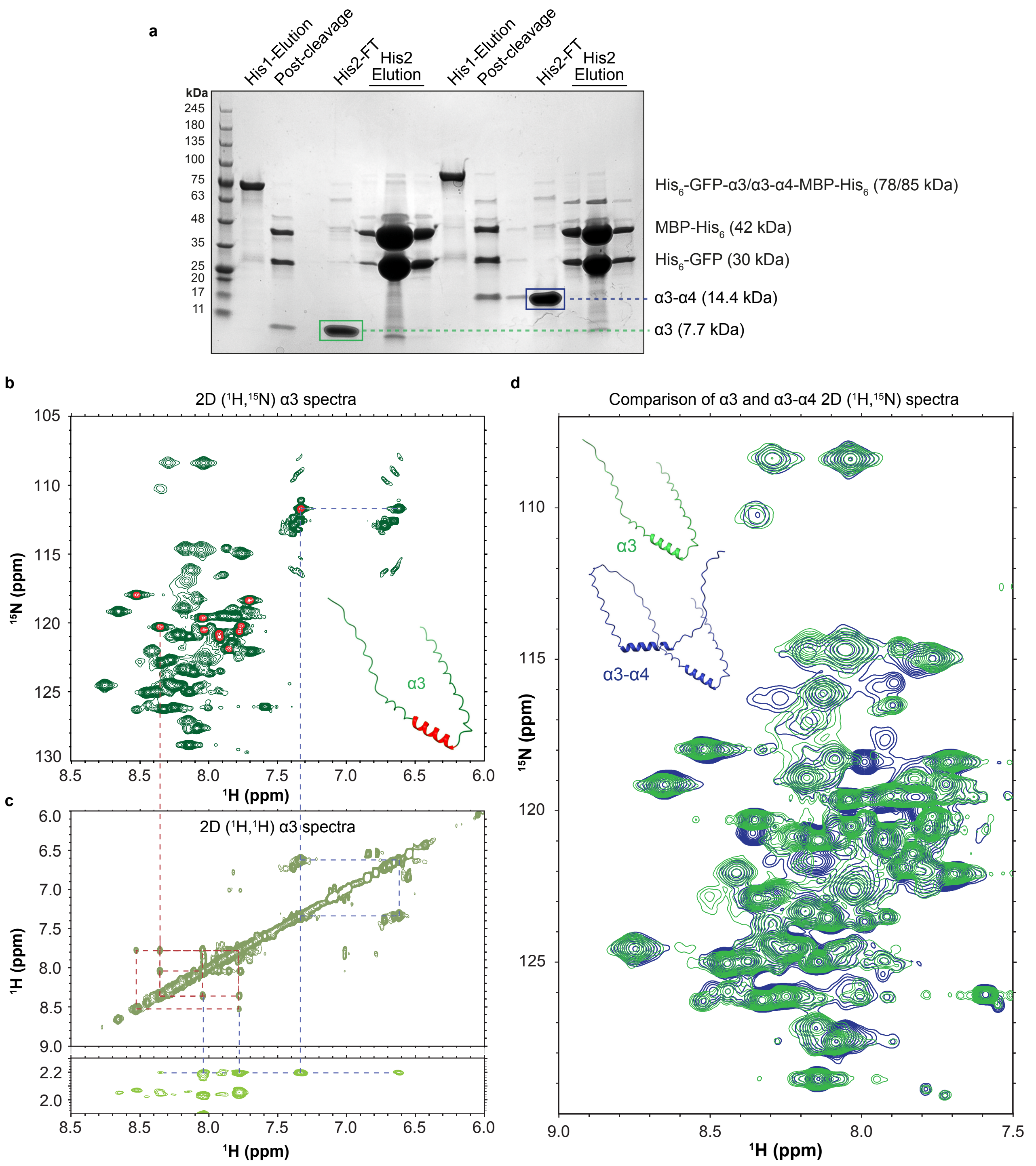

Supplementary Figure 9 | 2D NMR spectroscopy confirms the presence of pre-formed α-helices in CsLinker. a, Purification of the α3 and α3-α4 CsLinker fragments. SDS-PAGE analysis of the first nickel affinity product (His1), the samples following overnight cleavage, the flow through from the second nickel affinity purification (His2) and the elution. Annotations show the expected migration distances of the various species. The purification was completed once. **b**, 2D (^{1}H,^{15}N) SOFAST HMQC spectrum of the α3 fragment in solution. The low ^{1}H spectra dispersion of the amide-proton resonances (~1.5 ppm) is typical of proteins with significant intrinsic disorder. Approximately 60 of the 62 expected cross peaks are visible in the 2D spectrum (the 73 residue α3 construct has 11 prolines). **c**, Regions of a 2D (^{1}H, ^{1}H) NOESY spectrum of the same fragment in a, showing approximately 10 NOE cross-peaks in the amide-proton region (red dashed lines), which is consistent with the formation of a stable α-helix, as per the predicted structure. Consistent with the primary sequence of α3, the α-helical region contains at least one glutamine residue, as evidenced by the observation of NOEs between backbone amide and side chain amide protons and aliphatic protons (blue dashed lines). **d**, Overlay of the 2D (^{1}H, ^{15}N) SOFAST HMQC spectra of α3 (green) and α3-α4 (blue) showing significant overlap in the position of the cross-peaks. The small number of non-overlapping cross peaks observed is consistent with the small sequence differences between the α3 and α3-α4 repeat regions.

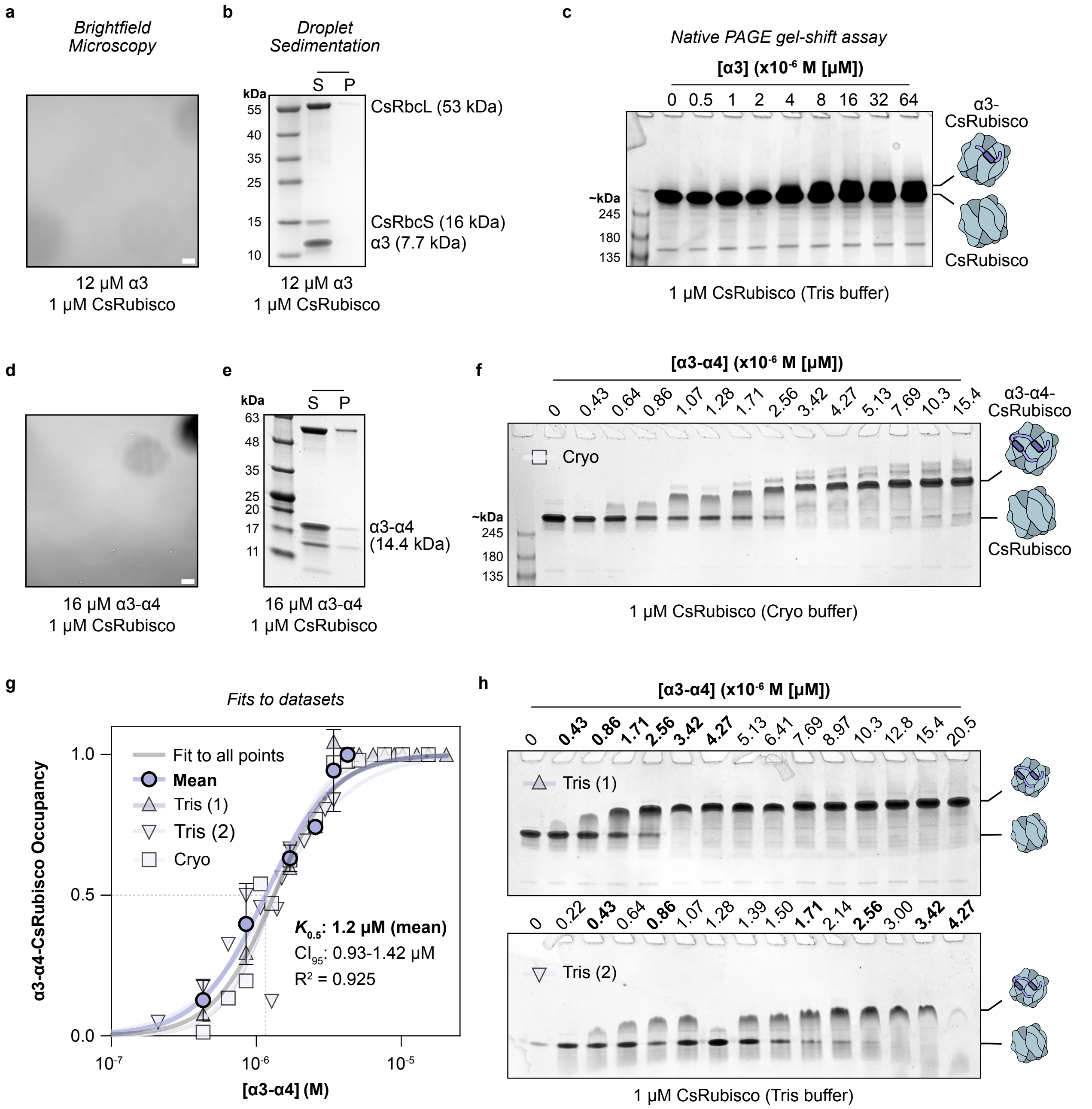

Supplementary Figure 10 | α3 and α3-α4 CsLinker fragments do not phase separate CsRubisco. a, Brightfield microscopy image of α3-CsRubisco mix, scale bar 5 μm. **b**, Droplets sedimentation assay of α3-CsRubisco mix. **c**, Native PAGE gel-shift assay of CsRubisco incubated with increasing concentrations of α3 CsLinker fragment in Tris buffer of low ionic strength (50 mM Tris-HCl, pH 8.0, 50 mM NaCl). **d**, Brightfield microscopy of α3-α4-CsRubisco mix. **e**, Droplet sedimentation of α3-α4-CsRubisco mix. **f**, Native PAGE gel-shift assay of CsRubisco incubated with increasing concentrations of α3-α4 in cryo buffer (200 mM sorbitol, 50 mM HEPES, 50 mM KOAc, 2 mM $Mg(OAc)_2 \cdot 4H_2O$ and 1 mM $CaCl_2$ at pH 6.8). **g**, Quantification of α3-α4-CsRubisco complex occupancy from native PAGE gel shift assays in f and h, derived from the inverse occupancy of the unbound CsRubisco state. Datasets were fitted separately as well as in a fit to all points using a Hill coefficient model ($Y = x^h / (K_{0.5}{}^h + x^h)$), where h is the Hill coefficient. The 'mean' dataset was derived from the bolded replicate values in the Tris (1) and Tris (2) experiments in h. A fit to this dataset was used to derive the $K_{0.5}$ of the α3-α4-CsRubisco complex as presented in Fig. 2a. **h**, Native PAGE gel-shift assays completed with CsRubisco and : α3-α4 in Tris buffer. Bolded concentrations were used to derive the 'Mean' dataset in g.

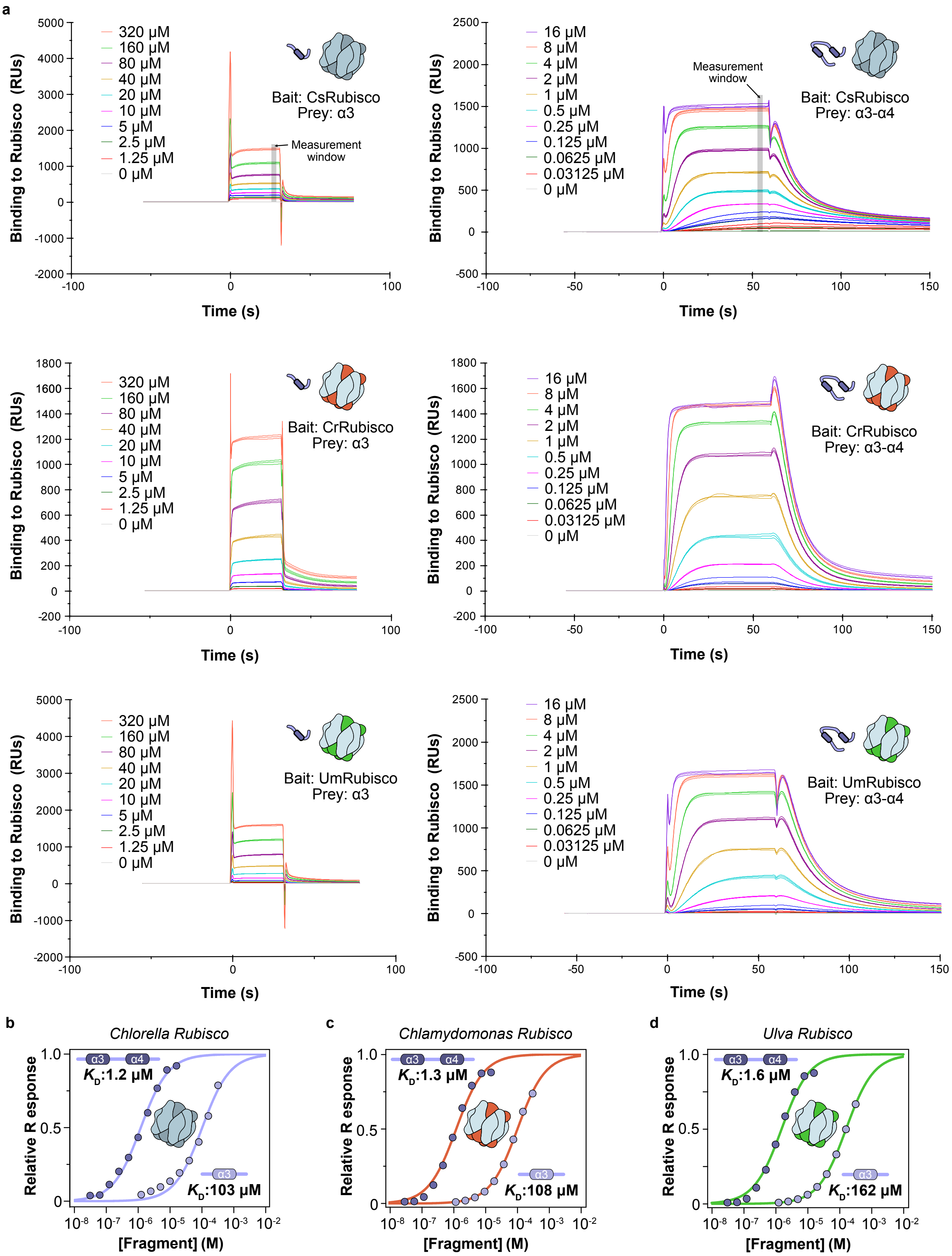
a
Binding to Rubisco (RUs)
Time (s)
320 µM
160 µM
80 µM
40 µM
20 µM
10 µM
5 µM
2.5 µM
1.25 µM
0 µM
Measurement window
Bait: CsRubisco
Prey: α3
16 µM
8 µM
4 µM
2 µM
1 µM
0.5 µM
0.25 µM
0.125 µM
0.0625 µM
0.03125 µM
0 µM
Bait: CsRubisco
Prey: α3-α4
Bait: CrRubisco
Prey: α3
Bait: CrRubisco
Prey: α3-α4
Bait: UmRubisco
Prey: α3
Bait: UmRubisco
Prey: α3-α4
b
Chlorella Rubisco
Relative Response
[Fragment] (M)
α3 α4
K_D:1.2 µM
α3
K_D:103 µM
c
Chlamydomonas Rubisco
K_D:1.3 µM
K_D:108 µM
d
Ulva Rubisco
K_D:1.6 µM
K_D:162 µM

Supplementary Figure 11 | Sensorgrams from SPR experiments. a, Raw sensorgrams from surface plasmon resonance experiments with the α3 and α3-α4 fragments at the indicated concentrations against Chlorella (Cs), *Chlamydomonas* (Cr) and *Ulva* (Um) Rubiscos. In the top panels, the window in which the SPR response was measured is indicated. **b**, Relative SPR response curves for α3 and α3-α4 fragments against CsRubisco as presented in Fig. 3b. SPR response is normalized to the fitted B_{max} value obtained from fit of the raw data. n=3, error bars = S.D. **c**, SPR response curve against *Chlamydomonas* Rubisco. **d**, SPR response curve against *Ulva* Rubisco.

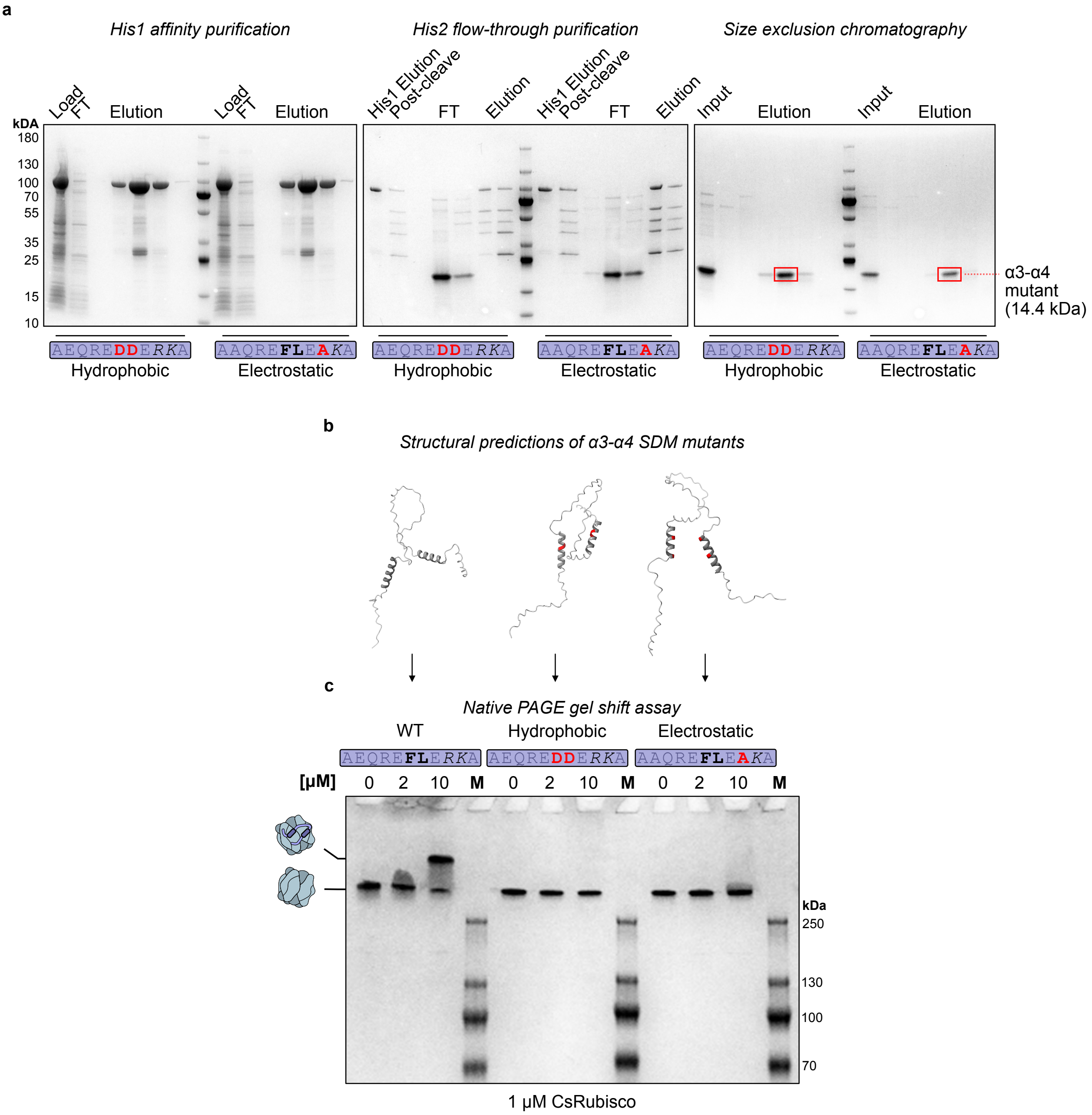

Supplementary Figure 12 | Purification of α3-α4 SDM variants and native PAGE analysis. a, Purification strategy for the hydrophobic and electrostatic α3-α4 mutants produced by site-directed mutagenesis. The purification was completed once and not repeated. (**b**, AlphaFold 2 structural predictions of the WT and mutant α3-α4 fragments, with the mutated residues shown in red, according to the schematics in a. **c**, Native PAGE gel shift assay completed with the mutant fragments compared to WT.

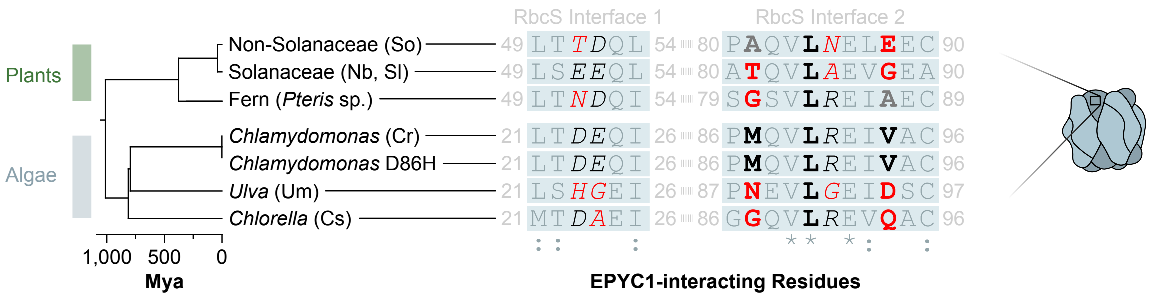

Supplementary Figure 13 | Comparison of EPYC1-interacting interfaces in green lineage RbcS sequences. Residues shown to interact with EPYC1 in a previous study[32] are colored black, with hydrophobic residues in bold and electrostatic residues in italics. Residues that do not share similar properties in other species are shown in red and stylized according to the corresponding residues in the *Chlamydomonas* sequence. No sequence was available for the *Adiantum* RbcS, so a *Pteris* fern RbcS sequence is presented instead.

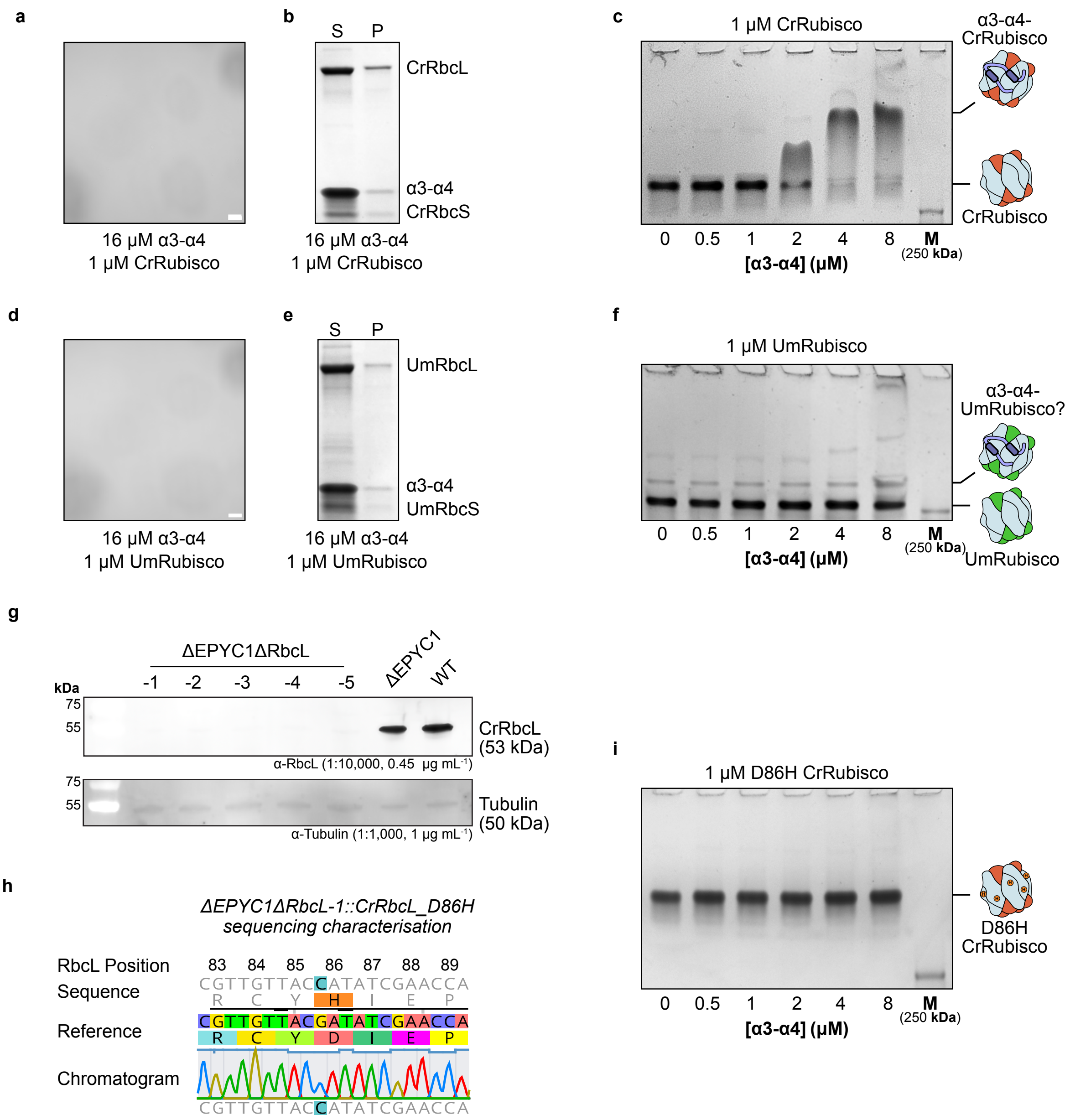

Supplementary Figure 14 | Cross-reactivity of α3-α4 and D86H RbcL mutation. a, Brightfield microscopy of α3-α4-CrRubisco solution at the indicated concentrations, scale bar = 5 μm. **b,** Droplet sedimentation assay of the same solution as in a. **c,** Native PAGE gel shift assay of 1 μm CrRubisco incubated with increasing concentrations of α3-α4 demonstrating shift to a higher order α3-α4-CrRubsico complex. **d,** Microscopy of α3-α4-UmRubisco solution. **e,** Droplet sedimentation assay of the same solution as in d. **f,** Native PAGE gel shift assay of α3-α4-UmRubisco solutions demonstrating possible complex formation at higher concentrations of α3-α4. **g,** Western blot analysis of RbcL protein presence in ΔEPYC1ΔRbcL knockout lines compared to the parental ΔEPYC1 and WT lines. Tubulin is used as a loading control. **h,** Sequencing of the ΔEPYC1ΔRbcL::CrRbcL_D86H strain that was complemented with a point mutated version of CrRbcL to introduce the D86H substitution. **i,** Native PAGE gel shift assay with α3-α4 and D86H mutated CrRubisco indicating lack of complex formation.

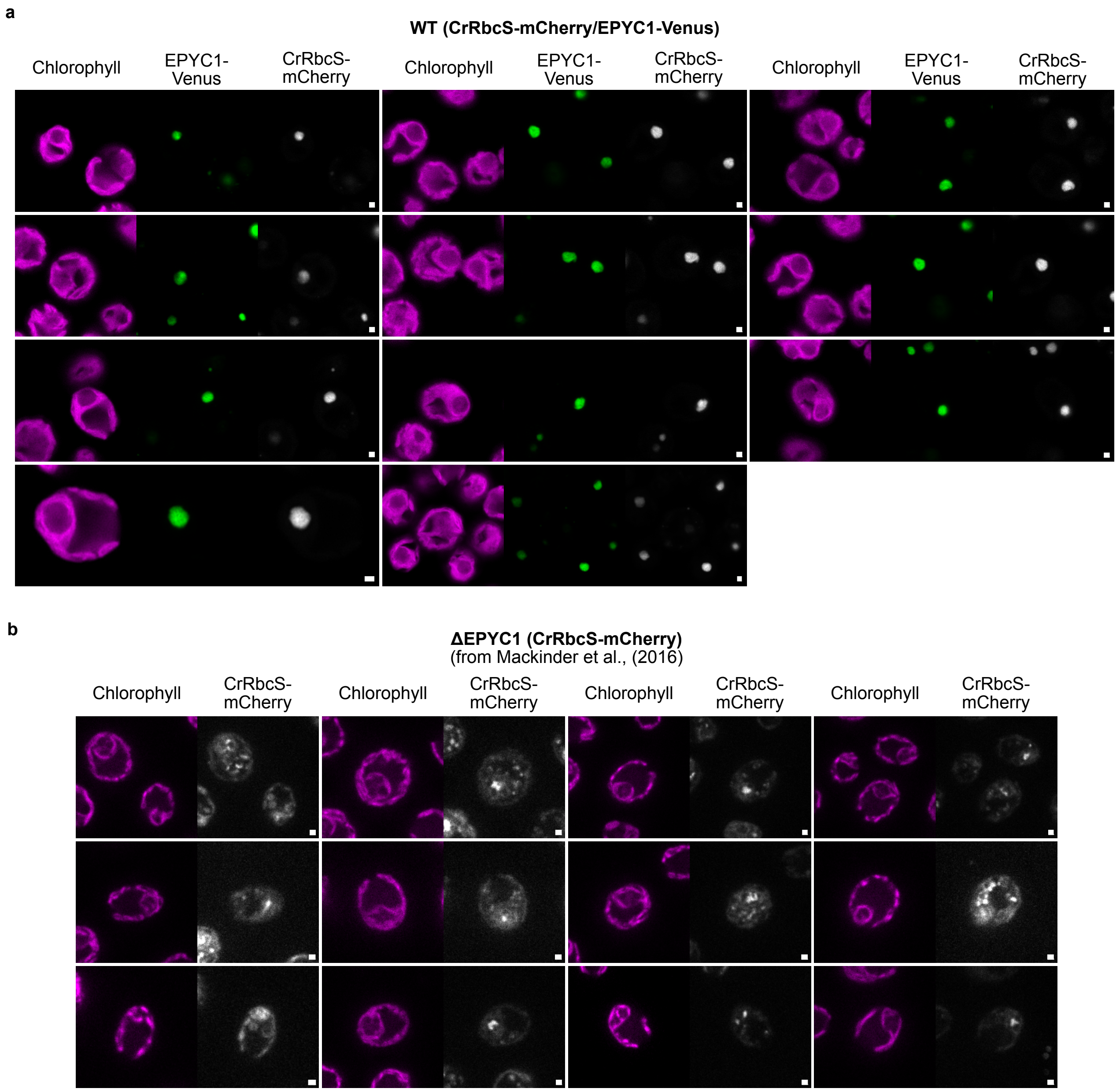

Supplementary Figure 15 | Rubisco localization in WT and ΔEPYC1 *Chlamydomonas*. a, Confocal fluorescence microscopy images of tagged RbcS and EPYC1 in the WT background strain. **b,** Images of tagged RbcS in the ΔEPYC1 background strain. Scale bars in a and b = 1 μm**.** These images were used for quantification in Extended Data Fig. 6.

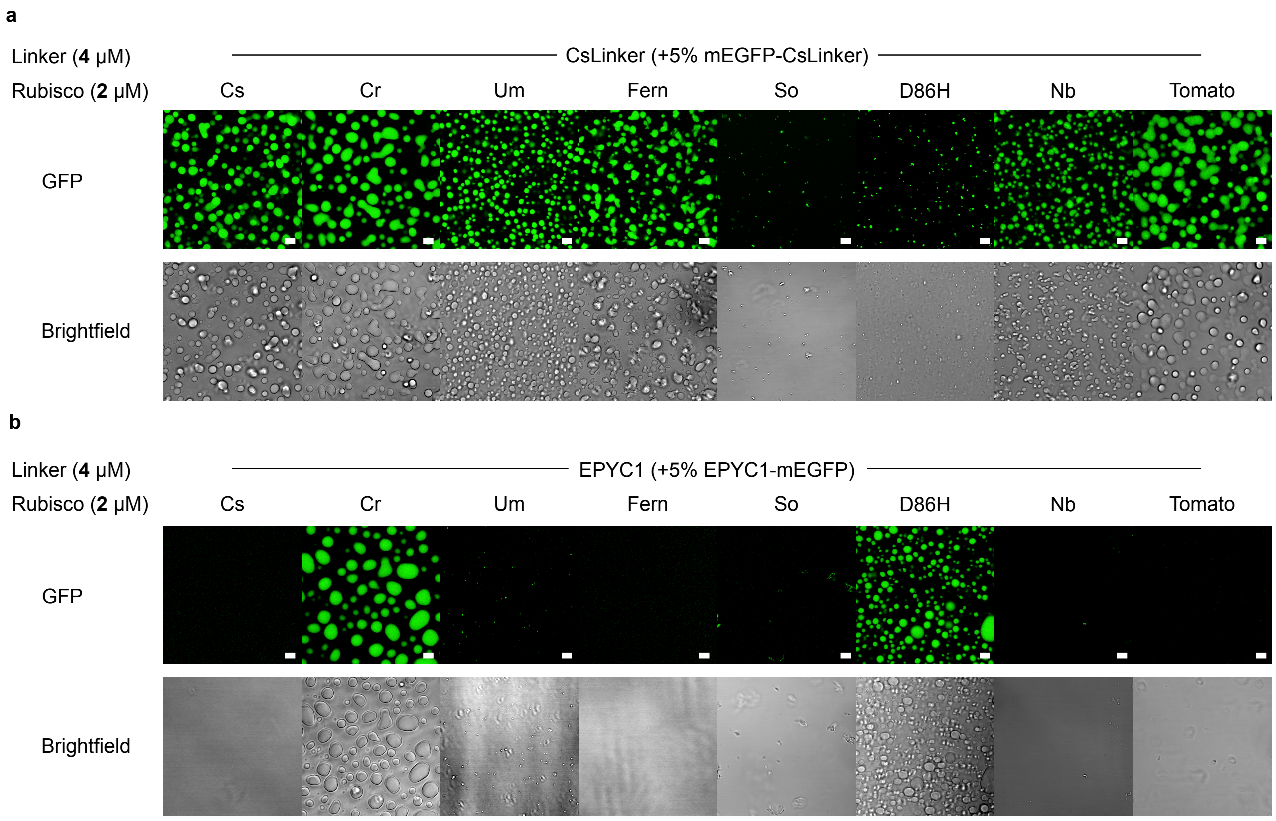

Supplementary Figure 16 | Brightfield images of droplets. a, Confocal fluorescence microscopy and accompanying brightfield images of CsLinker and different Rubiscos as shown in Figure 5. **b**, Confocal fluorescence microscopy and accompanying brightfield images of EPYC1 and different Rubiscos as shown in Figure 5. Scale bars in a and b = 5 μm.

Supplementary Tables

Supplementary Table 1 | CsLinker and RbcL co-immunoprecipitation results.
See associated excel worksheet.

Supplementary Table 2 | CO_2 response differential gene expression analysis.
See associated excel worksheet.

Supplementary Table 3 | BLAST results of CsLinker in the NCBI non-redundant database. Presence of sequence conserved Rubisco binding motifs (RBMs) is indicated. In *Notodromas monachal* and *Hypsibius exemplaris* no RBMs are present and sequence similarity is through the low complexity spacer regions.

Scientific Name	Query Cover	E value	Per. ident	Accession	RBMs present
Chlorella sorokiniana	100%	2.00E-112	65.71	PRW44308.1	Y
Chlorella ohadii	100%	5.00E-71	66.2	KAI7840023.1	Y
Chlorella sorokiniana	90%	9.00E-57	63.35	PRW50772.1	Y
Chlorella vulgaris	99%	6.00E-47	50.69	KAI3429224.1	Y
Chlorella variabilis	99%	2.00E-46	48.64	XP_005851975.1	Y
Micractinium conductrix	93%	5.00E-39	52.56	PSC68468.1	Y
Micractinium conductrix	93%	2.00E-35	51.88	PSC68430.1	Y
Chlorella vulgaris	97%	4.00E-32	52.73	KAI3429225.1	Y
Chlorella vulgaris	99%	4.00E-32	48.77	KAI3425152.1	Y
Micractinium conductrix	79%	1.00E-25	46.04	PSC72476.1	Y
Micractinium conductrix	79%	2.00E-25	46.04	PSC72477.1	Y
Micractinium conductrix	98%	8.00E-22	40.97	PSC69993.1	Y
Micractinium conductrix	98%	1.00E-21	40.97	PSC69992.1	Y
Chlorella variabilis	89%	3.00E-14	42.21	XP_005844057.1	Y
Chlorella vulgaris	90%	3.00E-13	43.01	KAI3430698.1	Y
Chlorella variabilis	90%	1.00E-10	38.06	XP_005850249.1	Y
Notodromas monacha	44%	0.019	39.77	CAD7276740.1	N
Chlorella vulgaris	55%	0.022	42.44	KAI3427970.1	Y
Hypsibius exemplaris	46%	0.041	42.2	OWA53275.1	N

Supplementary Table 4 | Quantification of immunogold-labelled RbcL in the pyrenoid.

Image number	Pyrenoid	Non-pyrenoid	% Pyrenoid	
1	35	2	94.6	
2	48	0	100.0	
3	105	10	91.3	
4	27	0	100.0	
7	158	4	97.5	
8	61	4	93.8	
			96.2	**Average**
			3.5	**S.D.**

Supplementary Table 5 | Standard curve values from absolute quantification experiments.

Purified Protein Standards					
**Sample ID**	**[Input] (pM)**	**MS1 Peak Area**	**Sample ID**	**[Input] (pM)**	**MS1 Peak Area**
CsLinker 1	126000	2.86E+05	CsRbcL 1	27000	9.31E+04
CsLinker 2	126000	1.14E+05	CsRbcL 2	27000	2.07E+05
CsLinker 1	1260000	2.77E+06	CsRbcL 1	270000	7.80E+05
CsLinker 2	1260000	3.81E+06	CsRbcL 2	270000	1.93E+06
CsLinker 1	12600000	4.24E+07	CsRbcL 1	2700000	1.94E+07
CsLinker 2	12600000	2.42E+07	CsRbcL 2	2700000	1.10E+07

Supplementary Table 6 | Measured values from whole cell lysate in absolute quantification.

Chlorella cell analysis (from 6E+6 cells)				
**Sample ID**	**Analyte**	**MS1 Peak Area**	**Analyte**	**MS1 Peak Area**
Chlorella 1a	CsLinker	1.11E+06	CsRbcL	9.57E+05
Chlorella 1b	CsLinker	1.34E+06	CsRbcL	1.26E+06
Chlorella 2a	CsLinker	9.45E+05	CsRbcL	9.60E+05
Chlorella 2b	CsLinker	1.19E+06	CsRbcL	1.23E+06
Chlorella 3a	CsLinker	8.92E+05	CsRbcL	1.03E+06
Chlorella 3b	CsLinker	1.06E+06	CsRbcL	1.10E+06

Supplementary Table 7 | Calculation of CsRubisco chloroplast concentration from absolute quantification data.

CsRubisco MS1 intensity (rep 1)	1110011.8	fmol (/5613)	197.8
CsRubisco MS1 intensity (rep 2)	1095989.3	fmol (/5613)	195.3
CsRubisco MS1 intensity (rep 3)	1067985	fmol (/5613)	190.3
		average fmol per sample	194.4
		average mol per sample	1.94E-13
		mol / cell (/6E+6)	3.24E-20
		[chloroplast] (M) (/1.1E-14)	2.95E-6
		**[chloroplast] (μM)**	**2.95** ± 0.06 S.D.
		molecules / cell (av. mol*NA)	1.95E+04

Supplementary Table 8 | Calculation of CsLinker chloroplast concentration from absolute quantification data.

CsLinker MS1 intensity (rep 1)	1224976	fmol (/2641)	463.8
CsLinker MS1 intensity (rep 2)	1064847	fmol (/2641)	403.2
CsLinker MS1 intensity (rep 3)	975129.7	fmol (/2641)	369.2
		average fmol per sample	412.1
		average mol per sample	4.12E-13
		mol / cell (/6E+6)	6.87E-20
		[chloroplast] (M) (/1.1E-14)	6.24E-6
		**[chloroplast] (μM)**	**6.24** ± 0.73 S.D.
		molecules / cell (av. mol*NA)	4.13E+04

Supplementary Table 9 | Calculation of CsRubisco chloroplast concentration from western blot data.

Intensity of 100 ng band	3349.175
Intersect no. of cells (/6688)	5.008E+06
ng per cell (/5.008E+06)	1.997E-05
pg per cell (*1000)	1.997E-02
mg per cell (/1E-19)	1.997E-11
volume chloroplast (mL)	1.100E-11
[chloroplast] (mg mL^{-1})	1.82
**[chloroplast] (μM) (/0.55)**	3.30

Supplementary Table 10 | Fluorescence recovery after photobleaching (FRAP) analysis.
See associated excel workbook.

Supplementary Table 11 | Hill model fit parameters for α3-α4 native PAGE gel-shift assay data.

	Experiment				
	Tris (1)	Tris (2)	Cryo	'Mean'	All data
$K_{0.5}$ (μM)	1.323	1.319	1.292	1.161	1.327
CI_{95} ($K_{0.5}$)	1.138-1.528	1.009-1.656	1.163-1.438	0.9311-1.422	1.206-1.454
Hill coefficient	2.465	1.567	2.47	1.876	2.075
CI_{95} (hill coeff.)	1.968-3.172	0.9824-2.449	1.971-3.161	1.374-2.568	1.747-2.428
R^2	0.9809	0.8488	0.9791	0.925	0.9441

Supplementary Table 12 | Fit parameters for SPR data.

	Experiment					
	**α3-CsRubisco**	**α3-CrRubisco**	**α3-UmRubisco**	**α3-α4-CsRubisco**	**α3-α4-CrRubisco**	**α3-α4-UmRubisco**
K_D	102.5	108.3	162.3	1.213	1.300	1.556
CI_{95} (K_D)	79.88-132.5	102.3-114.8	157.6-167.2	1.107-1.329	1.157-1.460	1.404-1.723
Fitted B_{max}	1860	1648	2378	1634	1683	1886
CI_{95} (B_{max})	1684-2075	1608-1689	2345-2413	1593-1677	1626-1742	1827-1946
R^2	0.9797	0.9988	0.9998	0.9955	0.9922	0.9943

Supplementary Table 13 | Collection, refinement, and validation of cryo-EM models.

**Data Collection and Processing**		
Magnification	240,000	
Voltage (kV)	200	
Electron fluence (e-/Å^2)	50	
Pixel Size (Å^2)	0.574	
Symmetry	D4	C1
Figure for map	Ext. Data Fig. 4g	Ext. Data Fig. 5a
Initial Particles	237,035	591,696 (8x expanded)
Final Particles	73,962	133,171
Map resolution (Å)	2.39	2.77
FSC Threshold	0.143	0.143
Map resolution range (Å)	8.05-2.23	9.42-2.29
**Refinement**	**CsRubisco**	**α3-α4-CsRubisco**
Map sharpening *B* factor (Å^2)	50	45
Model Composition		
Non-H atoms	35979	35836
Residues	4424	4440
Water	1267	1011
Mean B factors (Å^2)		
Protein	44.00	44.10
Water	42.40	47.97
RMS deviations		
Bond length (Å)	0.008	0.009
Bond angles (°)	1.164	1.171
**Validation**		
MolProbity Score	1.30	1.30
Clashscore	2.09	2.20
Poor rotamers %	0	0
Ramachandran plot		
Favored %	95.41	95.61
Allowed %	4.59	4.39
Outliers	0	0
PDB Code	8Q04	8Q05

Supplementary Table 14 | PDBePISA analysis of α3-α4-CsRubisco complex interface.

**Salt bridges**			
α3-α4 residues (atom)	CsRbcL residue (atom)	Distance (Å)	Secondary structure
**Arg176 (NE)**	**Glu51 (OE2)**	**3.04**	**αB**
Arg176 (NE)	Glu51 (OE1)	3.93	αB
Arg176 (NH2)	Glu51 (OE2)	2.96	αB
**Lys177 (NZ)**	**Asp86 (OD1)**	**3.56**	**βC**
**Hydrogen bonds**			
Gly165 (O)	Gly92 (N)	2.66	CD loop
Gln170 (NE2)	***Glu93 (O)***	***3.29***	***CD loop***
Gln170 (NE2)	***Gln96 (O)***	***3.30***	***CD loop***
Arg176 (NE)	Glu51 (OE2)	3.04	αB
Arg176 (NH2)	Glu51 (OE2)	2.96	αB
Lys177 (NZ)	Ile87 (O)	2.68	βC
Lys177 (NZ	Asp86 (OD1)	3.56	βC
**α3-α4 Interface**			
α3-α4 residue	Accessible surface area (Å^2)	Buried surface area (Å^2)	% Buried
Gly164	121.72	28.64	23.5
Gly165	84.64	31.20	36.9
**Leu166**	**105.80**	**36.00**	**34.0**
Ser167	63.01	15.15	24.0
Ala168	87.15	0.00	0.0
Glu169	73.76	23.02	31.2
Gln170	***95.46***	***95.46***	***100.0***
Arg171	46.76	0.00	0.0
Glu172	48.95	0.00	0.0
**Phe173**	**90.09**	**90.09**	**100.0**
**Leu174**	**82.49**	**12.39**	**15.0**
Glu175	63.88	0.00	0.00
Arg176	158.68	62.48	39.4
Lys177	149.89	64.24	42.9
Ala178	89.52	0.00	0.0
Arg179	124.39	0.00	0.0

Supplementary Table 15 | Amino acid sequences.

Sequence of interest
Fluorescent protein
TEV cleavage site
Flexible linker
Scar amino acids
Transit peptide
Mutated residue
Affinity tag

**mEGFP-CsLinker** from: *His-mEGFP-TEV-CsLinker*	MGSS*HHHHHH*GSSVSKGEELFTGVVPILVELDGDVNGHKFSVRGEGEG<br>DATNGKLTLKFICTTGKLPVPWPTLVTTLTYGVQCFSRYPDHMKQHDF<br>FKSAMPEGYVQERTISFKDDGTYKTRAEVKFEGDTLVNRIELKGIDFK<br>EDGNILGHKLEYNFNSHNVYITADKQKNGIKANFKIRHNVEDGSVQLA<br>DHYQQNTPIGDGPVLLPDNHYLSTQSKLSKDPNEKRDHMVLLEFVTAA<br>GITLGMDELYKGIEENLYFQSNAMATAQLSAEQKAFLERKARESRSPA<br>PVETRGRSAPRPRSASPVGRSSRTPAPASYSAPAASYSSAPANGLSAE<br>QREFMERKARESRSPAPVETRGRSAPRPRSASPRPTSRTPAPASYSAP<br>AASYTAPAAAPAGGLSAEQREFLERKARESRSPAPVETRGRSAPRPRS<br>ASPRPTSRTPAPASYSAPAASYSAPAAAPAGGLSAEQREFMERKARES<br>RSFSAPVETRGRSASARPSARPSSRTPTPVSNSGVRSAMSSGSSAGLS<br>AEQREFLERKARESRSPAPAAASQSSSYGSAASGLSAEQREFLERKAR<br>ESRSFSAPAPTRGRSSTPRRR*
**CsLinker** from: *His-mEGFP-TEV-CsLinker-TEV-MBP-His*	SNAMATAQLSAEQKAFLERKARESRSPAPVETRGRSAPRPRSASPVGR<br>PSSRTPAPASYSAPAASYSSAPANGLSAEQREFMERKARESRSPAPVE<br>TRGRSAPRPRSASPRPTSRTPAPASYSAPAASYTAPAAAPAGGLSAEQ<br>REFLERKARESRSPAPVETRGRSAPRPRSASPRPTSRTPAPASYSAPA<br>ASYSAPAAAPAGGLSAEQREFMERKARESRSFSAPVETRGRSASARPS<br>ARPSSRTPTPVSNSGVRSAMSSGSSAGLSAEQREFLERKARESRSPAP<br>AAASQSSSYGSAASGLSAEQREFLERKARESRSFSAPAPTRGRSSTPR<br>RRENLYFQ
**α3** from: *His-mEGFP-TEV-α3-TEV-MBP-His*	STPAPASYSAPAASYTAPAAAPAGGLSAEQREFLERKARESRSPAPVE<br>TRGRSAPRPRSASPRPTSRENLYFQ
**α3-α4** from: *His-mEGFP-TEV-α3-α4-TEV-MBP-His*	STPAPASYSAPAASYTAPAAAPAGGLSAEQREFLERKARESRSPAPVE<br>TRGRSAPRPRSASPRPTSRTPAPASYSAPAASYSAPAAAPAGGLSAEQ<br>REFMERKARESRSFSAPVETRGRSASARPSARPSSRENLYFQ
**α3(F173DL174D)-α4(F239DM240D)** from: *His-mEGFP-TEV-α3(F173DL174D)-α4(F239DM240D)-TEV-MBP-His*	STPAPASYSAPAASYTAPAAAPAGGLSAEQRE**DD**ERKARESRSPAPVE<br>TRGRSAPRPRSASPRPTSRTPAPASYSAPAASYSAPAAAPAGGLSAEQ<br>RE**DD**ERKARESRSFSAPVETRGRSASARPSARPSSRENLYFQ
**α3(E169AR176A)-α4(E235AR242A)** from: His-mEGFP-TEV-α3(E169AR176A)-α4(E235AR242A)-TEV-MBP-His	STPAPASYSAPAASYTAPAAAPAGGLSA**A**QREFLE**A**KARESRSPAPVE<br>TRGRSAPRPRSASPRPTSRTPAPASYSAPAASYSAPAAAPAGGLSA**A**Q<br>REFME**A**KARESRSFSAPVETRGRSASARPSARPSSRENLYFQ
**mVenus-CsLinker** from: mVenus-CsLinker chloroplast expression vector	MVSKGEELFTGVVPILVELDGDVNGHKFSVSGEGEGDATYGKLTLKLI<br>CTTGKLPVPWPTLVTTLGYGLQCFARYPDHMKQHDFFKSAMPEGYVQE<br>RTIFFKDDGNYKTRAEVKFEGDTLVNRIELKGIDFKEDGNILGHKLEY<br>NYNSHNVYITADKQKNGIKANFKIRHNIEDGGVQLADHYQQNTPIGDG<br>PVLLPDNHYLSYQSKLSKDPNEKRDHMVLLEFVTAAGITLGMDELYKG

	SAMATAQLSAEQKAFLERKARESRSPAPVETRGRSAPRPRSASPVGRP<br>SSRTPAPASYSAPAASYSSAPANGLSAEQREFMERKARESRSPAPVET<br>RGRSAPRPRSASPRPTSRTPAPASYSAPAASYTAPAAAPAGGLSAEQR<br>EFLERKARESRSPAPVETRGRSAPRPRSASPRPTSRTPAPASYSAPAA<br>SYSAPAAAPAGGLSAEQREFMERKARESRSFSAPVETRGRSASARPSA<br>RPSSRTPTPVSNSGVRSAMSSGSSAGLSAEQREFLERKARESRSPAPA<br>AASQSSSYGSAASGLSAEQREFLERKARESRSFSAPAPTRGRSSTPRR<br>R*
**CsLinker** from: CsLinker chloroplast expression vector	MAMATAQLSAEQKAFLERKARESRSPAPVETRGRSAPRPRSASPVGRP<br>SSRTPAPASYSAPAASYSSAPANGLSAEQREFMERKARESRSPAPVET<br>RGRSAPRPRSASPRPTSRTPAPASYSAPAASYTAPAAAPAGGLSAEQR<br>EFLERKARESRSPAPVETRGRSAPRPRSASPRPTSRTPAPASYSAPAA<br>SYSAPAAAPAGGLSAEQREFMERKARESRSFSAPVETRGRSASARPSA<br>RPSSRTPTPVSNSGVRSAMSSGSSAGLSAEQREFLERKARESRSPAPA<br>AASQSSSYGSAASGLSAEQREFLERKARESRSFSAPAPTRGRSSTPRR<br>R*
**CrRbcL (D86H)** from: P-67 D86H RbcL vector	MVPQTETKAGAGFKAGVKDYRLTYYTPDYVVRDTDILAAFRMTPQPGV<br>PPEECGAAVAAESSTGTWTTVWTDGLTSLDRYKGRCY**H**IEPVPGEDNQ<br>YIAYVAYPIDLFEEGSVTNMFTSIVGNVFGFKALRALRLEDLRIPPAY<br>VKTFVGPPHGIQVERDKLNKYGRGLLGCTIKPKLGLSAKNYGRAVYEC<br>LRGGLDFTKDDENVNSQPFMRWRDRFLFVAEAIYKAQAETGEVKGHYL<br>NATAGTCEEMMKRAVCAKELGVPIIMHDYLTGGFTANTSLAIYCRDNG<br>LLLHIHRAMHAVIDRQRNHGIHFRVLAKALRMSGGDHLHSGTVVGKLE<br>GEREVTLGFVDLMRDDYVEKDRSRGIYFTQDWCSMPGVMPVASGGIHV<br>WHMPALVEIFGDDACLQFGGGTLGHPWGNAPGAAANRVALEACTQARN<br>EGRDLAREGGDVIRSACKWSPELAAACEVWKEIKFEFDTIDKL*
**NbRbcS-mCherry** from: NbRbcS-mCherry *Nicotiana* transient expression vector	MASSMLSSATMVASPAQATMVAPFNGLKSSAAFPATRKANNDITSITS<br>NGGRVNCMASSVLSSAAVATRSNVAQANMVAPFTGLKSAASFPVSRKQ<br>NLDITSIASNGGRVQCMQVWPPINKKKYETLSYLPDLSVEQLLSEIEY<br>LLKNGWVPCLEFETERGFVYREHHKSPGYYDGRYWTMWKLPMFGCTDA<br>TQVLAEVEEAKKAYPQAWIRIIGFDNVRQVQCISFIAYKPEGYGSGSA<br>GSAAGSGEFMVSKGEEDNMAIIKEFMRFKVHMEGSVNGHEFEIEGEGE<br>GRPYEGTQTAKLKVTKGGPLPFAWDILSPQFMYGSKAYVKHPADIPDY<br>LKLSFPEGFKWERVMNFEDGGVVTVTQDSSLQDGEFIYKVKLRGTNFP<br>SDGPVMQKKTMGWEASSERMYPEDGALKGEIKQRLKLKDGGHYDAEVK<br>TTYKAKKPVQLPGAYNVNIKLDITSHNEDYTIVEQYERAEGRHSTGGM<br>DELYK*
**CsLinker-tGFP** from: CsLinker-tGFP *Nicotiana* transient expression vector	MASSMLSSATMVASPAQATMVAPFNGLKSSAAFPATRKANNDITSITS<br>NGGRVNCGGAMATAQLSAEQKAFLERKARESRSPAPVETRGRSAPRPR<br>SASPVGRPSSRTPAPASYSAPAASYSSAPANGLSAEQREFMERKARES<br>RSPAPVETRGRSAPRPRSASPRPTSRTPAPASYSAPAASYTAPAAAPA<br>GGLSAEQREFLERKARESRSPAPVETRGRSAPRPRSASPRPTSRTPAP<br>ASYSAPAASYSAPAAAPAGGLSAEQREFMERKARESRSFSAPVETRGR<br>SASARPSARPSSRTPTPVSNSGVRSAMSSGSSAGLSAEQREFLERKAR<br>ESRSPAPAAASQSSSYGSAASGLSAEQREFLERKARESRSFSAPAPTR<br>GRSSTPRRRGSRGSGSESDESGLPAMEIECRITGTLNGVEFELVGGGE<br>GTPEQGRMTNKMKSTKGALTFSPYLLSHVMGYGFYHFGTYPSGYENPF<br>LHAINNGGYTNTRIEKYEDGGVLHVSFSYRYEAGRVIGDFKVMGTGFP<br>EDSVIFTDKIIRSNATVEHLHPMGDNDLDGSFTRTFSLRDGGYYSSVV<br>DSHMHFKSAIHPSILQNGGPMFAFRRVEEDHSNTELGIVEYQHAFKTP<br>DADAGEE*
**CsLinker-mNeonGreen** from: CsLinker-	MASSMLSSATMVASPAQATMVAPFNGLKSSAAFPATRKANNDITSITS<br>NGGRVNCGGAMATAQLSAEQKAFLERKARESRSPAPVETRGRSAPRPR

mNeonGreen *Nicotiana* transient expression vector	SASPVGRPSSRTPAPASYSAPAASYSSAPANGLSAEQREFMERKARES<br>RSPAPVETRGRSAPRPRSASPRPTSRTPAPASYSAPAASYTAPAAAPA<br>GGLSAEQREFLERKARESRSPAPVETRGRSAPRPRSASPRPTSRTPAP<br>ASYSAPAASYSAPAAAPAGGLSAEQREFMERKARESRSFSAPVETRGR<br>SASARPSARPSSRTPTPVSNSGVRSAMSSGSSAGLSAEQREFLERKAR<br>ESRSPAPAAASQSSSYGSAASGLSAEQREFLERKARESRSFSAPAPTR<br>GRSSTPRRRGSGSAGSAAGSGEFMVSKGEEDNMASLPATHELHIFGSI<br>NGVDFDMVGQGTGNPNDGYEELNLKSTKGDLQFSPWILVPHIGYGFHQ<br>YLPYPDGMSPFQAAMVDGSGYQVHRTMQFEDGASLTVNYRYTYEGSHI<br>KGEAQVKGTGFPADGPVMTNSLTAADWCRSKKTYPNDKTIISTFKWSY<br>TTGNGKRYRSTARTTYTFAKPMAANYLKNQPMYVFRKTELKHSKTELN<br>FKEWQKAFTDVMGMDELYK*

Supplementary Table 16 | Oligonucleotides sequences.
See associated excel worksheet

Supplementary Table 17 | Reagent catalog numbers and availability.
See associated excel worksheet
