# Supplementary material for: A promiscuous mechanism to phase separate eukaryotic carbon fixation in the green lineage
Source: Nat Plants. 2024 Oct 9;10(11):1801–13. doi: 10.1038/s41477-024-01812-x (PMC11570498; doi:10.1038/s41477-024-01812-x)
Supplement: Supplementary file 2 — Reporting Summary [file 41477_2024_1812_MOESM2_ESM.pdf]

nature portfolio

Corresponding author(s): Luke Mackinder

Last updated by author(s): Jul 23, 2024

Reporting Summary

Nature Portfolio wishes to improve the reproducibility of the work that we publish. This form provides structure for consistency and transparency in reporting. For further information on Nature Portfolio policies, see our Editorial Policies and the Editorial Policy Checklist.

Statistics

For all statistical analyses, confirm that the following items are present in the figure legend, table legend, main text, or Methods section.

n/a	Confirmed	
☐	☒	The exact sample size (*n*) for each experimental group/condition, given as a discrete number and unit of measurement
☐	☒	A statement on whether measurements were taken from distinct samples or whether the same sample was measured repeatedly
☐	☒	The statistical test(s) used AND whether they are one- or two-sided<br>*Only common tests should be described solely by name; describe more complex techniques in the Methods section.*
☒	☐	A description of all covariates tested
☒	☐	A description of any assumptions or corrections, such as tests of normality and adjustment for multiple comparisons
☐	☒	A full description of the statistical parameters including central tendency (e.g. means) or other basic estimates (e.g. regression coefficient) AND variation (e.g. standard deviation) or associated estimates of uncertainty (e.g. confidence intervals)
☐	☒	For null hypothesis testing, the test statistic (e.g. *F*, *t*, *r*) with confidence intervals, effect sizes, degrees of freedom and *P* value noted<br>*Give P values as exact values whenever suitable.*
☒	☐	For Bayesian analysis, information on the choice of priors and Markov chain Monte Carlo settings
☒	☐	For hierarchical and complex designs, identification of the appropriate level for tests and full reporting of outcomes
☒	☐	Estimates of effect sizes (e.g. Cohen's *d*, Pearson's *r*), indicating how they were calculated

Our web collection on statistics for biologists contains articles on many of the points above.

Software and code

Policy information about availability of computer code

Data collection	for SPR: Biacore T200 control software v3.2.1 (Cytiva);<br>for single-particle cryo-electron microscopy: EPU version 3.7 (ThermoFisher);<br>for confocal microscopy using Zeiss LSM880: ZEN black 2.3 SP1 FP3 (Zeiss);<br>for confocal microscopy using Zeiss LSM980: ZEN blue 3.8 (Zeiss)
Data analysis	for NMR: TopSpin 4 and CCPN analysis v3.2;<br>for gel quantification, confocal image processing and quantification: Fiji v1.5;<br>for image stabilization in Fiji: Image Stabilizer plugin v1;<br>for SPR: BIAevaluation v4.1;<br>for cloning and genetic analysis: Geneious Prime 2023;<br>for single-particle cryo-electron microscopy: Relion v3.1.2, MotionCor2, CTFFIND4, UCSF Chimera v1.16, UCSF ChimeraX v1.6, Coot v0.9.8, Phenix v1.2, Molprobity v4.5, PDBePISA v1.5;<br>for sequence alignments: MAFFT v7.4 run in Geneious Prime 2023;<br>for mVenus sequence optimisation: Codon Usage Optimizer v0.92;<br>for mass spectrometry: Progenesis QI v4.2, Mascot v2.8, DIA-NN v1.88, FragPipe-Analyst v21;<br>for structural prediction: AlphaFold 2 as part of a ColabFold v1.5 colab notebook;<br>for chloroplast target peptide prediction: TargetP 2.0;<br>for repeat detection (as part of FLIPPer): XSTREAM 1.73;<br>for disorder prediction (as part of FLIPPer v1): metapredict V2;<br>for disorder prediction (prior to FLIPPer v1): IUPred2A;<br>for code of FLIPPer v1: https://github.com/james-r-barrett/FLIPPer

for differential gene expression analysis of RNA-seq data: Salmon v1.10;
for statistical analyses: Prism 10;
for curve fitting and data plotting: Python3 utilizing packages matplotlib, scipy;
for BLAST analysis: NCBI BLASTp web tool (https://blast.ncbi.nlm.nih.gov/Blast.cgi#)

For manuscripts utilizing custom algorithms or software that are central to the research but not yet described in published literature, software must be made available to editors and reviewers. We strongly encourage code deposition in a community repository (e.g. GitHub). See the Nature Portfolio guidelines for submitting code & software for further information.

Data

Policy information about availability of data

All manuscripts must include a data availability statement. This statement should provide the following information, where applicable:

- Accession codes, unique identifiers, or web links for publicly available datasets
- A description of any restrictions on data availability
- For clinical datasets or third party data, please ensure that the statement adheres to our policy

Proteomics data were deposited in MassIVE, with ProteomeXchange identifier PXD044179.
Electron density maps were deposited in EMDB with accession codes EMD-18049 (D4) and EMD-18050 (C1), and their corresponding coordinates in the PDB with accession codes 8Q04 and 8Q05 respectively.
Raw confocal imaging data associated with figures 2, 4 and 5, and extended data figures 8, 9, 10, 12, 19, 21 and 23 are available in a Zenodo repository (10.5281/zenodo.11492220).
Differential gene expression analysis was completed using publicly available dataset PRJNA343632 and processed data is available as a supplementary table.
Processed co-immunoprecipitation data are also included as a supplementary table.
All other associated source data, along with associated explanations and analyses are available in the associated Zenodo repository (10.5281/zenodo.11492220).

Research involving human participants, their data, or biological material

Policy information about studies with human participants or human data. See also policy information about sex, gender (identity/presentation), and sexual orientation and race, ethnicity and racism.

Reporting on sex and gender	n/a
Reporting on race, ethnicity, or other socially relevant groupings	n/a
Population characteristics	n/a
Recruitment	n/a
Ethics oversight	n/a

Note that full information on the approval of the study protocol must also be provided in the manuscript.

Field-specific reporting

Please select the one below that is the best fit for your research. If you are not sure, read the appropriate sections before making your selection.

☒ Life sciences ☐ Behavioural & social sciences ☐ Ecological, evolutionary & environmental sciences

For a reference copy of the document with all sections, see nature.com/documents/nr-reporting-summary-flat.pdf

Life sciences study design

All studies must disclose on these points even when the disclosure is negative.

Sample size	For analysis of Rubisco partitioning between the stroma and pyrenoid in Chlamydomonas, sample size selection was guided by the sample sizes used in a previous study (Mackinder et al., 2016, PNAS) that used the same approach.<br>For cryo-EM, the number of collected micrographs was limited by the time available on the instrument, but was maximized accordingly.
Data exclusions	No data were excluded from analysis.
Replication	The micrograph of the Chlamydomonas pyrenoid presented in figure 1a was from a single collection but is representative of collections under the same conditions (TP, low CO2) of the same strain (for examples, see: https://doi.org/10.1038/s41477-020-00811-y).<br><br>The co-IP experiments presented in figure 1e represent the results of 3 biological replicates (cultures grown separately) that were completed in parallel and submitted under the same LC-MS experiment.<br><br>The micrograph of the Chlorella pyrenoid immunogold labelled in figure 2a and associated data in Supplementary figure 5 were from a single

collection across biological all conditions.

Absolute quantification data presented in figure 2b represents measurement of three biological replicate samples (grown in separate cultures) completed concurrently.

The representative image of the droplet reconstitution in figure 2c is representative of multiple independent collections such as those presented in figure 2h, i, j and k, though this instance was the only experiment in which both components were labelled. The associated droplet sedimentation assay is repeated several times throughout (e.g. figure S6c,d and S7c).

The CsLinker and CsRubisco droplet sedimentation titrations in figure 2d/e were completed twice as separate replicate experiments concurrently (see supplementary fig. 7).

The FRAP curves presented in figure 2f/g represent the indicated number of replicate measurements (n = 24, 26 respectively) from experiments completed using at least 2 different droplet formations in experiments performed on different days. For more information, see the image dates in Supplementary Table 10 and the associated Zenodo repository.

The timeline snapshots presented in figures 2h-k are representative examples taken from experiments that were completed as above for 2f/g. Other (non-presented) examples can be seen throughout the unprocessed images provided in the Zendo repository.

The native PAGE band shift presented in figure 3a was only completed once in this format, but is representative of repeat experiments performed over wider concentration ranges as presented in supplementary figure 10. For the adjacent quantification, replicate measurements were taken from 2 independent experiments that were completed over slightly different dilution regimes, as presented in supplementary figure 10h. The characteristic shift in these experiments was highly reproducible, as evidenced by the overlaid fits in supplementary figure 10g.

The SPR response curves presented in figure 3b are representative of 3 replicate experiments that were completed using the same SPR chip and reagents, and are considered technical replicates.

The native PAGE experiments of the SDM fragments presented in figure 3g are representative of a single non-repeated experiment. In the uncropped version of this figure shown in supplementary figure 12c, the WT fragment was run alongside the SDM fragment to ensure the characteristic shift was still observed.
The droplet assays presented in figures 4b and 4h were completed on multiple independent occasions (see extended data Fig. 8 for more examples). The control experiment in which no linker was added was only imaged on one occasion.

The Chlamydomonas images presented in figures 4c, f and i are representative of multiple cell images (see extended data fig. 6 and supplementary fig. 15). For the ΔEPYC1 (CrRbcS-mCherry/mVenus-CsLinker) line, 3 independently produced lines were imaged under the same conditions. For the other lines, only a single biological replicate was imaged.

The spot test growth assays presented in figures 4d, g and j are cropped from extended data fig. 7. In these experiments, multiple independently produced ΔEPYC1::CsLinker biological replicate samples were assayed under two conditions (pH 8.0 and pH 8.2) that were completed on separate occasions.

The droplet images presented in figures 5b and c are representative of single non-repeated experiments at the indicated conditions for Ulva, Fern, Spinach and Cr(D86H) Rubiscos, though the results are agreeable with similar experiments completed at lower concentrations (see Extended data fig. 8). For Chlorella Rubisco, the same experiment, completed independently is presented throughout.

The in planta localisations are representative of experiments that were completed independently in separate infiltrations, of which more examples are presented in extended data fig. 10.

The CO2 response western blots in extended data figure 1b represent a single experiment that was not repeated, in which a single culture was analysed from each growth condition. The abundance of the CsLinker under low CO2 conditions was confirmed in a separate experiment (supplementary fig. 2d).

The pyrenoid enrichment, blotting and immunofluorescence experiments presented in extended data fig. 2 were all completed using a single pyrenoid enriched fraction from a single biological replicate. The protocol was optimised over other (non-presented) enrichment trial experiments with highly similar results.

As for fig. 2d-k, the FRAP experiment in extended data fig. 3 are representative of at least two separate droplet formations and measurement sessions, as indicated by dates of images in supplementary table 10.

All cryo-EM results presented in extended data figures 4 and 5 were from a single, non-repeated data collection at the indicated conditions.

As outlined for figures 4c, f and i, the images of the ΔEPYC1 (CrRbcS-mCherry/mVenus-CsLinker) in extended data fig. 6 are from 3 independently produced lines with equivalent genotypes. See the raw images in the associated zenodo repository for more information. All the images were captured in the same imaging session under the same conditions, alongside those of the single WT and ΔEPYC1 (CrRbcS-mCherry) lines.

The blots of CsLinker presence in extended data figs. 6f and g were completed once without repetition.

The spot test growth assays presented in extended data fig. 7 were completed at the two different pHs in separate experiments on different calendar weeks. In both experiments, at least 4 independently produced lines for ΔEPYC1::CsLinker were analysed.

Unless stated, all the droplet assays completed in extended data fig. 8 represent single, non-repeated observations.

The in planta imaging in extended figs. 10a, b and d are representative of single non-repeated infiltrations and imaging experiments. The images in extended data fig. 10c is representative of 2 independent infiltration and imaging experiments.

	Throughout the study, no findings could not be reproduced.
Randomization	No randomization approaches were required in this study as no experiments were affected by known covariates that could be controlled.
Blinding	Blinding approaches were not used throughout the study as most experiments were observational in nature. Though relevant, blinding was not used for the analysis of RbcL immungold localisation relative to the control experiment. This did not affect the reported outcomes however, due to the almost total lack of immunogold particles in the control experiments.

Reporting for specific materials, systems and methods

We require information from authors about some types of materials, experimental systems and methods used in many studies. Here, indicate whether each material, system or method listed is relevant to your study. If you are not sure if a list item applies to your research, read the appropriate section before selecting a response.

Materials & experimental systems

n/a	Involved in the study	
☐	☒	Antibodies
☐	☒	Eukaryotic cell lines
☒	☐	Palaeontology and archaeology
☒	☐	Animals and other organisms
☒	☐	Clinical data
☒	☐	Dual use research of concern
☐	☒	Plants

Methods

n/a	Involved in the study	
☒	☐	ChIP-seq
☒	☐	Flow cytometry
☒	☐	MRI-based neuroimaging

Antibodies

Antibodies used	Custom primary antibodies were raised in rabbit to a C-terminal Rubisco Large subunit peptide (EVWKEIKFEFETIDTL) and a region of CsLinker (PTPVSNSGVRSAMSSG) by Yenzym antibodies LLC, USA. Anti-Tubulin antibody raised in mouse (T6074) was purchased from Sigma-Aldrich.<br><br>Secondary antibodies used for detection:<br>- Gold conjugate goat anti-rabbit antibody for immunoelectron microscopy (G7402, Merck)<br>- Alexa Fluor Plus 488 conjugate goat anti-rabbit antibody for western blot and immunofluorescence (A-11008, ThermoFisher Scientific)<br>- Alexa Fluor 555 conjugate goat anti-mouse antibody for immunofluorescence (A-21422, ThermoFisher Scientific)
Validation	Both custom primary antibodies were validated by western blotting against purified proteins and on whole cell lysates. For both antibodies, a specific band at the expected molecular weight was observed.<br><br>The anti-tubulin primary antibody was not validated in Chlorella sorokiniana using purified protein but has been validated by Sigma-Aldrich in Chlamydomonas, which shares high sequence similarity with Chlorella. Use of the antibody in our study resulted in a single band of ~55 kDa in both Chlorella and Chlamydomonas, in line with the expected molecular weight of the proteins. The specificty of T6074 has also previously been validated in Chlamydomonas (see e.g.: https://doi.org/10.1105/tpc.17.00149).

Eukaryotic cell lines

Policy information about cell lines and Sex and Gender in Research

Cell line source(s)	Chlorella sorokiniana UTEX1230 (SAG211-8k) was used.<br><br>Chlamydomonas reinhardtii strains used:<br>- WT (CC-4533)<br>- ΔEPYC1 (CC-5360)
Authentication	Cell lines were not authenticated as part of this study, but have been authenticated since their retrieval in the lab.
Mycoplasma contamination	n/a
Commonly misidentified lines (See ICLAC register)	n/a

Plants

Seed stocks	Nicotiana material seed was a gift of Prof Alison Smith of the John Innes Centre.<br>Tomato material was grown from seed of variant VTO325.<br>Spinach material was acquired from the Co-operative food supermarket, Hull Road, York and was sold as 'Baby Spinach'.<br>Fern material was acquired from Dean's Garden Centre, York and denoted as 'Adiantum raddianum' Fragrans variant.
Novel plant genotypes	Genes were transiently expressed in Nicotiana benthamiana by agrobacterium-mediated transformation, as described in the text.
Authentication	No authentication of novel genotypes was completed, other than confocal imaging of transgene expression.
