# Supplementary figures and images for: A promiscuous mechanism to phase separate eukaryotic carbon fixation in the green lineage
Source: Nat Plants. 2024 Oct 9;10(11):1801–13. doi: 10.1038/s41477-024-01812-x (PMC11570498; doi:10.1038/s41477-024-01812-x)

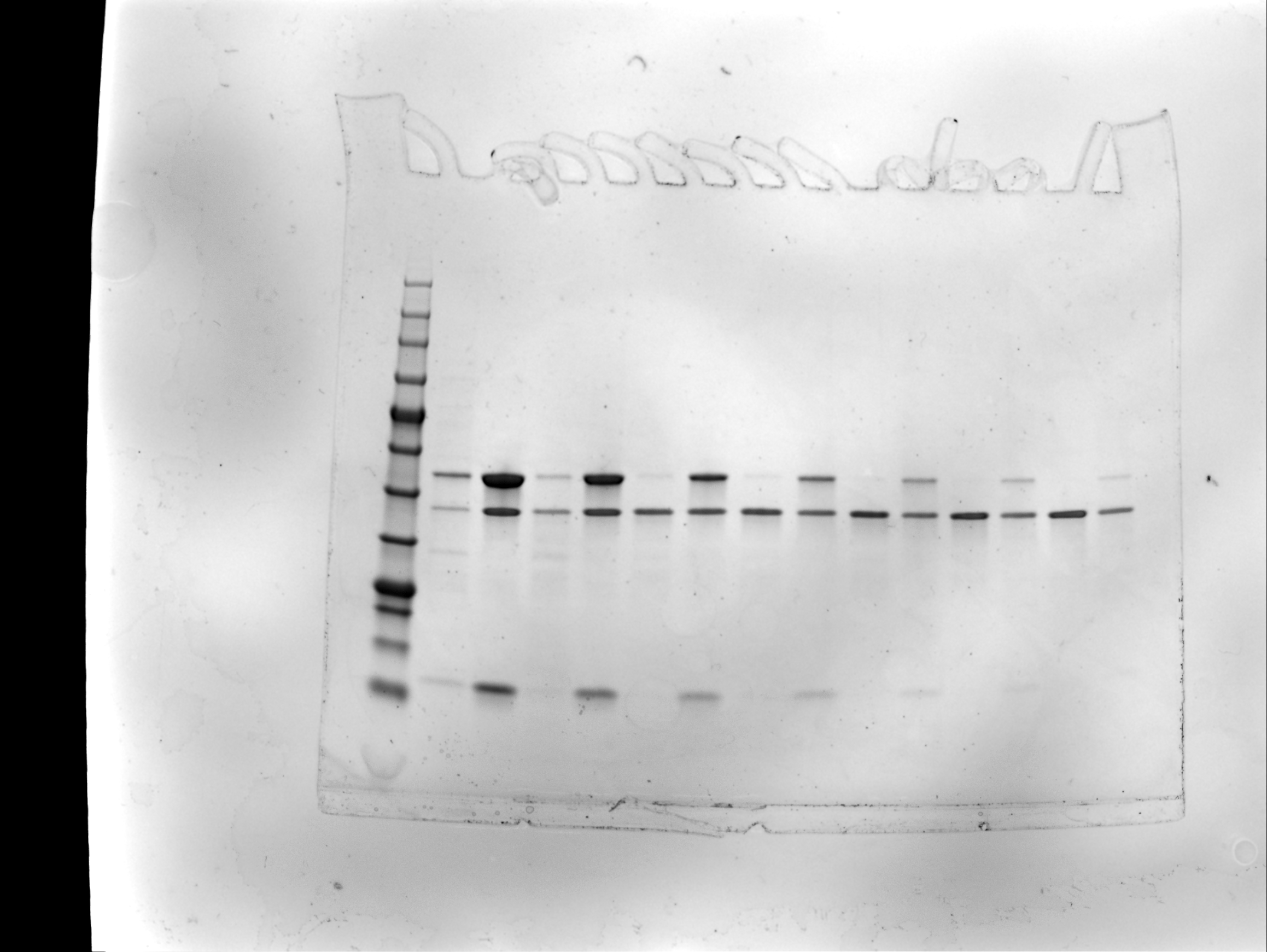

Supplement: Supplementary file 5 — Unprocessed SDS-PAGE gel for Fig. 2c. [file 41477_2024_1812_MOESM5_ESM.tif]

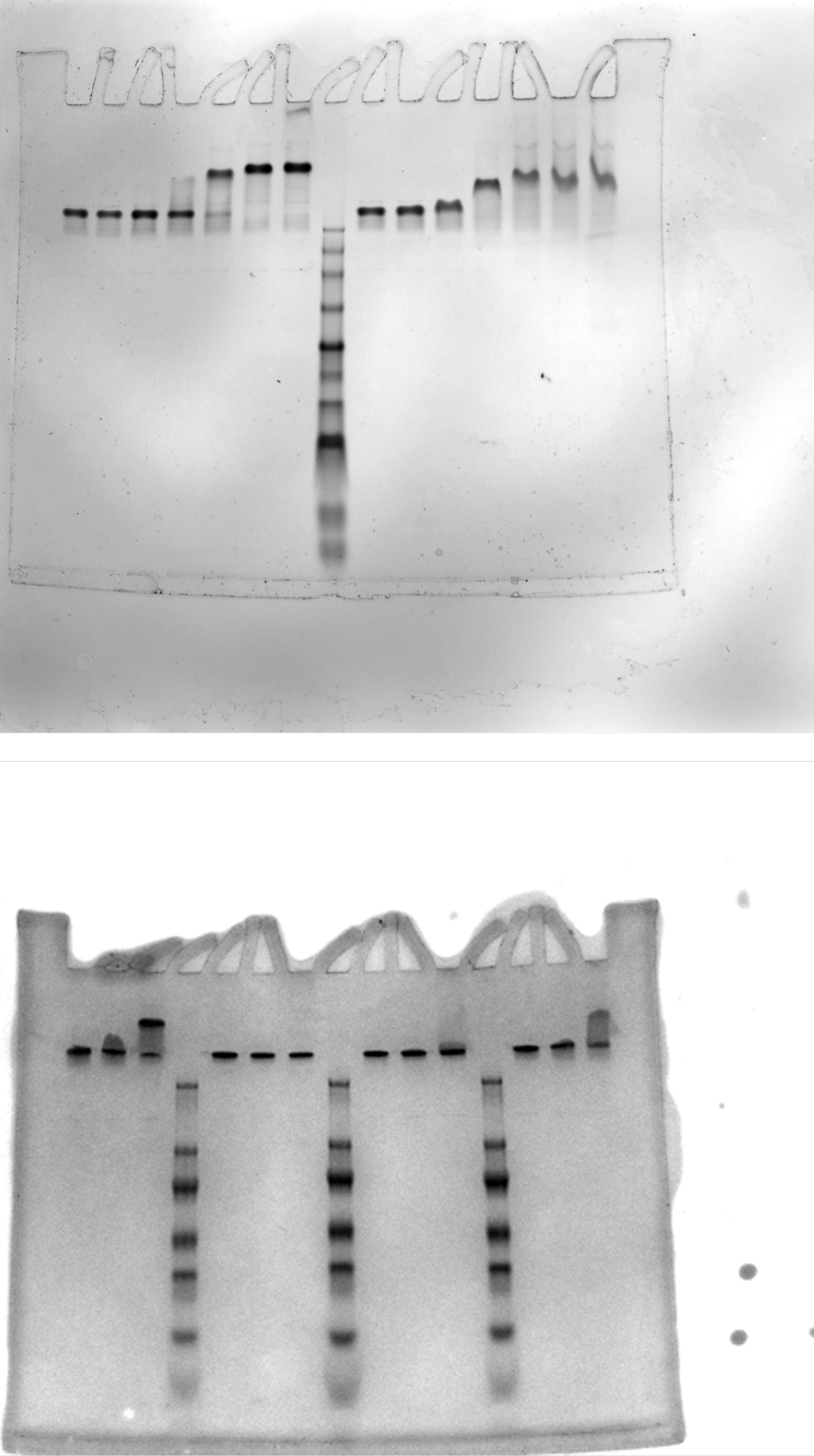

Supplement: Supplementary file 6 — Unprocessed native PAGE gel for Fig. 3a,g. [file 41477_2024_1812_MOESM6_ESM.tiff]
